# Joint host-pathogen genomic analysis identifies hepatitis B virus mutations associated with human *NTCP* and HLA class I variation

**DOI:** 10.1016/j.ajhg.2024.04.013

**Published:** 2024-05-14

**Authors:** Zhi Ming Xu, Gnimah Eva Gnouamozi, Sina Rüeger, Patrick R. Shea, Maria Buti, Henry LY. Chan, Patrick Marcellin, Dylan Lawless, Olivier Naret, Matthias Zeller, Arne Schneuing, Andreas Scheck, Thomas Junier, Darius Moradpour, Ondrej Podlaha, Vithika Suri, Anuj Gaggar, Mani Subramanian, Bruno Correia, David Gfeller, Stephan Urban, Jacques Fellay

**Affiliations:** 1 School of Life Sciences, École Polytechnique Fédérale de Lausanne, Lausanne, Switzerland; 2 Swiss Institute of Bioinformatics, Lausanne, Switzerland; 3 Department of Infectious Diseases, Molecular Virology, University Hospital Heidelberg, Heidelberg, Germany; 4 Institute for Genomic Medicine, Columbia University, New York, NY, USA; 5 Liver Unit, Hospital Universitario Vall d’Hebron and CIBEREHD del Instituto Carlos III, Barcelona, Spain; 6 The Chinese University of Hong Kong, Hong Kong, China; 7 Service d’Hépatologie, Hôpital Beaujon, Clichy, France; 8 Division of Gastroenterology and Hepatology, Lausanne University Hospital and University of Lausanne, Lausanne, Switzerland; 9 Gilead Sciences Inc, Foster City, CA, USA; 10 Department of Oncology UNIL-CHUV, Lausanne University Hospital, Ludwig Institute for Cancer Research, University of Lausanne, Lausanne, Switzerland; 11 German Center for Infection Research (DZIF), Partner Site Heidelberg, Heidelberg, Germany; 12 Precision Medicine Unit, Biomedical Data Science Center, Lausanne University Hospital and University of Lausanne, Lausanne, Switzerland

**Keywords:** host-pathogen genomics, hepatitis B virus, selection, immunogenetics, HBV entry, evolutionary genomics, escape variants, viral restriction

## Abstract

Evolutionary changes in the hepatitis B virus (HBV) genome could reflect its adaptation to host-induced selective pressure. Leveraging paired human exome and ultra-deep HBV genome-sequencing data from 567 affected individuals with chronic hepatitis B, we comprehensively searched for the signatures of this evolutionary process by conducting “genome-to-genome” association tests between all human genetic variants and viral mutations. We identified significant associations between an East Asian-specific missense variant in the gene encoding the HBV entry receptor NTCP (rs2296651, NTCP S267F) and mutations within the receptor-binding region of HBV preS1. Through *in silico* modeling and *in vitro* preS1-NTCP binding assays, we observed that the associated HBV mutations are in proximity to the NTCP variant when bound and together partially increase binding affinity to NTCP S267F. Furthermore, we identified significant associations between HLA-A variation and viral mutations in HLA-A-restricted T cell epitopes. We used *in silico* binding prediction tools to evaluate the impact of the associated HBV mutations on HLA presentation and observed that mutations that result in weaker binding affinities to their cognate HLA alleles were enriched. Overall, our results suggest the emergence of HBV escape mutations that might alter the interaction between HBV PreS1 and its cellular receptor NTCP during viral entry into hepatocytes and confirm the role of HLA class I restriction in inducing HBV epitope variations.

## Graphical abstract

**Figure:**
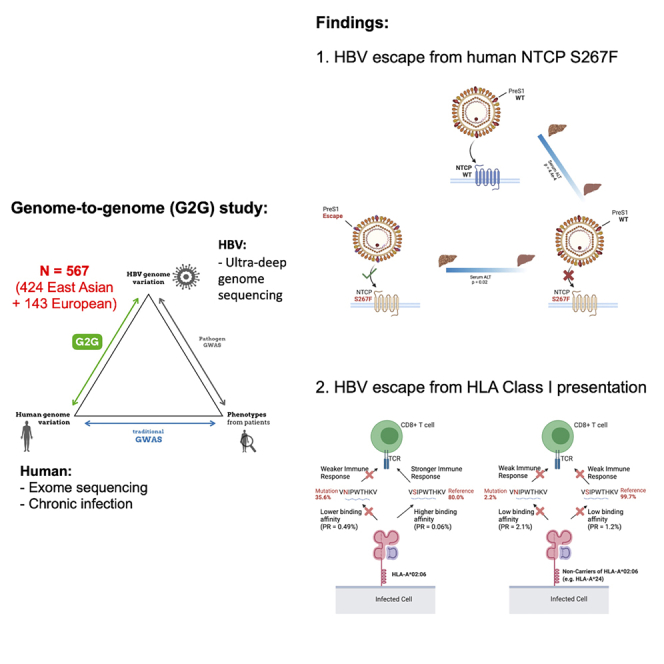

## Teaser

Host-induced selective pressure can shape viral evolution during chronic infections. By analyzing paired human and hepatitis B virus (HBV) genomic data, we pinpoint viral mutations associated with human NTCP and HLA-A variation, shedding light on HBV-evasion strategies that influence viral entry and HLA presentation.

## Introduction

Hepatitis B virus (HBV) infection is a major public health concern, with approximately 296 million chronically infected individuals as of 2019.^1^ Chronic hepatitis B (MIM: 610424) leads to an increased risk of developing cirrhosis and hepatocellular carcinoma (HCC; MIM: 114550).^2^ In 2019, 820,000 people died from HBV infection-related causes, mostly from cirrhosis and HCC.^1^

The human genetic basis of the susceptibility to HBV infection and the progression to or prognosis of chronic hepatitis B have been explored in numerous studies. Genome-wide association studies (GWASs) and candidate gene studies have had success in pinpointing specific loci associated with outcomes of HBV infection.^3^ Many of the identified variants map to immune-related genes, including human leukocyte antigen (HLA) class I and II,^4^^,^^5^ cytokines,^6^^,^^7^^,^^8^ and Toll-like receptors.^9^^,^^10^ Variants within non-immune-related genes have also been identified. In particular, an Asian-specific single-nucleotide polymorphism (SNP) (rs2296651), which encodes an amino-acid change (S267F) in the sodium taurocholate co-transporting polypeptide (NTCP; MIM: 182396), the entry receptor of HBV, has been associated with reduced susceptibility to HBV infection and more favorable clinical outcomes.^11^^,^^12^^,^^13^^,^^14^^,^^15^^,^^16^^,^^17^^,^^18^^,^^19^

A limitation of using GWASs to study infectious disease is the failure to consider the genetic variability of the pathogen. Because host and pathogen genetic determinants jointly determine the fitness of the pathogen and the prognosis of the disease, evolutionary changes occur on both genomes as a result of an “arms-race”-like evolutionary process.^20^ Selective pressure originating from host polymorphisms can induce escape mutations on the pathogen genomes, and on a much longer timescale, host polymorphisms that counteract these escape mutations could undergo positive selection at a population level. For viruses with a high mutation rate, intra-host evolution resulting from host genetic pressure has been shown to occur during chronic infections. For example, during HIV (MIM: 609423) infection, longitudinal studies have provided direct evidence of immune evasion in the form of escape mutations in HIV epitopes targeted by cytotoxic T lymphocytes (CTLs).^21^^,^^22^^,^^23^^,^^24^^,^^25^ Although HBV is a DNA virus, it has a mutation rate that is closer to that of RNA viruses than to other DNA viruses^26^ due to an error-prone reverse-transcription step in its replication cycle. As such, evidence of intra-host evolution and its role in the development of HCC has been observed for HBV.^27^ Cross-sectional studies have also supported the presence of CTL escape during HBV infection.^28^^,^^29^^,^^30^^,^^31^

In this study, we leveraged paired human exome and ultra-deep HBV genome-sequencing data from a cohort of 567 affected individuals with chronic hepatitis B. We utilized a “genome-to-genome” (G2G) approach^32^^,^^33^^,^^34^ where we performed a genomes-wide search for associations between all pairs of HBV amino-acid mutations and human SNPs while correcting for confounding factors including human and viral population structures. We identified HBV mutations that are associated with human genetic variations, reflecting host-induced selective pressure on the virus. Our results support the role of HLA class I variation in inducing HBV epitope variation and pinpoint potential escape mutations within HBV preS1 that alter preS1-NTCP interaction, which is a prerequisite for HBV internalization.

## Material and methods

### Study description

Paired human blood and pre-treatment HBV DNA samples were obtained from affected individuals with chronic hepatitis B enrolled in four phase three clinical trials that evaluated tenofovir-based antiviral regimens (GS-US-283-1062,^35^ GS-US-320-0108,^36^ GS-US-320-0110,^37^ and GS-US-330-1401^38^) conducted by Gilead Sciences. Individuals with East Asian ancestry were recruited from Australia (AUS) (*N* = 6), Canada (CAN) (*N* = 80), Spain (ESP) (*N* = 1), France (FRA) (*N* = 1), Great Britain (GBR) (*N* = 2), Hong Kong (HKG) (*N* = 94), Italy (ITA) (*N* = 1), Japan (JPN) (*N* = 32), South Korea (KOR) (*N* = 117), New Zealand (NZL) (*N* = 15), Russia (RUS) (*N* = 2), Singapore (SGP) (*N* = 4), and the United States (USA) (*N* = 69). Individuals with European ancestry were recruited from AUS (*N* = 2), CAN (*N* = 1), ESP (*N* = 1), GBR (*N* = 3), ITA (*N* = 22), NZL (*N* = 1), Poland (POL) (*N* = 20), Romania (ROU) (*N* = 34), RUS (*N* = 44), Turkey (TUR) (*N* = 11), and USA (*N* = 4). The trials excluded individuals with evidence of HCC, cirrhosis/hepatic decompensation, and co-infections with hepatitis C virus (HCV), hepatitis D virus (HDV), or HIV. Biomarkers of HBV infection including HBV viral load, hepatitis B surface-antigen (HBsAg) level, hepatitis B e antigen (HBeAg) status, and serum alanine aminotransferase (ALT) level were measured at baseline. Across the four trials, both HBeAg-positive and HBeAg-negative individuals were included. All study participants signed an informed consent form prior to screening and in accordance with local regulatory and ethics committee requirements. A summary of the demographical details of each study can be found in Table S1. Complete details can be found at https://clinicaltrials.gov/ (ClinicalTrials: NCT02579382, NCT01940341, NCT01940471, and NCT02174276).

### Exome sequences

High-quality genomic DNA was extracted from frozen peripheral blood samples using the Beckman Biomek FXp liquid handling system. Enrichment of the exonic regions of the genome was performed using Roche or IDT Exome Research Panel V1 capture kits (Integrated DNA Technologies, Coralville, IA USA). Short-read DNA sequencing was performed using Illumina HiSeq2500 or NovaSeq 6000 sequencers according to the manufacturer’s instructions. Initial quality-control filtering of the exome-sequencing reads, adapter masking, and conversion of BCL files to fastq format was performed using CASAVA software version 1.8 (https://support.illumina.com/sequencing/sequencing_software/bcl2fastq-conversion-software.html).

Sequencing reads were aligned to the GRCh37.87 (hs37d5.fa; https://ftp.1000genomes.ebi.ac.uk/vol1/ftp/technical/reference/phase2_reference_assembly_sequence/hs37d5.fa.gz) reference genome using the DRAGEN Bio-IT platform (https://support.illumina.com/sequencing/sequencing_software/dragen-bio-it-platform.html) v.2.0.1. Duplicate sequencing reads were removed using Picard (https://broadinstitute.github.io/picard/) v.2.2.1. Variant calling was performed with the Genome Analysis Tool Kit (GATK) HaplotypeCaller program (https://gatk.broadinstitute.org/hc/en-us) v.v3.6-0-g89b7209 according to GATK Best Practice guidelines^39^ using dbSNP v138 (https://www.ncbi.nlm.nih.gov/snp/). Local re-alignment of all insertion/deletions variants was performed using Smith-Waterman, and GATK Base Quality Score Recalibration was applied to single nucleotide variants (SNVs).

### Quality control

Poor-quality samples and variant calls were excluded using PLINK (https://www.cog-genomics.org/plink/) v.1.9^40^ by applying a sample-based and variant-based missingness threshold of 0.1. Variants that deviate from Hardy Weinberg equilibrium were also excluded by applying a filtering threshold of p $<1\times 10^{-6}$. Related individuals up to first-degree relatives were excluded using the KING software (https://www.kingrelatedness.com/) v.2.2.2.^41^

### Human genetic principal components

To obtain genetically determined ancestry of study participants, principal-component analysis (PCA) was first applied to the 1000 Genomes (1KG) reference samples (https://www.internationalgenome.org/data).^42^ Using the GCTA software v.1.91.7,^43^ PCA loadings were extracted from the 1KG samples and then applied to the genotypes of our study participants. We then compared self-reported race and genetically determined ancestry. We observed that most of those who self-report as White were most genetically similar to the European-ancestry group, while most of those who self-report as Asian were most genetically similar to the East Asian-ancestry group (Figure S1). To assign genetically determined ancestry labels, hierarchical clustering was applied using the Wald method based on Euclidean distances in the PC space. The dendrogram was cut such that study participants were clustered into two major ancestry groups (East Asian and European) and a separate group representing mostly South Asian ancestry. Participants assigned to the East Asian- and European-ancestry group, with self-reported race of Asian and White, respectively, were retained. Participants in other ancestry groups were excluded given the limited sample size with constrained statistical power to detect associations. PCs were then re-calculated for the retained participants to be used in subsequent analyses. We refer to participants within these two ancestry groups as the East Asian and European cohort, respectively.

### Imputation of HLA alleles and amino acid variants

The HLA-LA^44^ software (https://github.com/DiltheyLab/HLA-LA) was used to impute HLA alleles at the four-digit levels based on BAM files that included unaligned reads. A quality threshold (Q1 > 0.7) was applied to retain high-quality HLA allele inferences. To encode amino-acid variants of HLA, the PyHLA^45^ software (https://github.com/felixfan/PyHLA) was used to identify amino-acid changes associated with each HLA allele. HLA alleles and HLA amino-acid variants were then encoded as dosages (presence or absence), and Variant Call Format (VCF) files were constructed for subsequent analyses.

### Viral genome sequences

#### Viral genome sequencing and mapping

DNA isolation was performed using Qiagen MinElute kit for serum samples with viral load <100,000 IU/mL and Roche MagNA Pure robot 32 for samples with viral loads >100,000 IU/mL. The amplification of HBV whole genome was performed by DDL Diagnostics Laboratory (Rijswijk, Netherlands) following the modified protocol described in Gunther et al.^46^ Whole-genome HBV amplicons were sequenced on Illumina MiSeq with 150 bp paired-end reads. Low-quality bases (Q < 20) at 5′ and 3′ of each read were trimmed with Trimmomatic software (https://github.com/usadellab/Trimmomatic) v.0.35,^47^ and reads shorter than 50 bp were removed. Subsequently, paired reads were merged based on overlapping regions, and sequencing error correction was performed using PEAR software (https://cme.h-its.org/exelixis/web/software/pear/) v.0.9.6. Unmerged reads were discarded. Read mapping was performed with BWA software (https://bio-bwa.sourceforge.net/) v.0.7.9a, and the reference genome for each sample was chosen from HBVdb.ibcp.fr (https://hbvdb.lyon.inserm.fr/HBVdb/HBVdbIndex)^48^ given the patient’s genotype, which was determined via laboratory genotyping assay. NCBI accession numbers (https://www.ncbi.nlm.nih.gov/nuccore) are as follows: genotype A, NCBI: EU054331; genotype B, NCBI: AB219428; genotype C, NCBI: GQ924620; genotype D, NCBI: FJ904433; genotype F, NCBI: AY090458; and genotype H, NCBI: FJ356716. On average, a genome-wide coverage of approximately 6,800× was achieved per sample: 337 samples exceeded 7,000×, 202 samples between 5,000× and 7,000×, and 28 samples between 2,000× and 5,000×.

#### Viral mutations

Viral genome positions were normalized to the HBV genotype C (NCBI: GQ924620). At a given genome position, a nucleotide was treated as present if it exceeded the minimal intra-host frequency of 15%. In cases where multiple nucleotides were detected above minimal frequency, International Union of Pure and Applied Chemistry (IUPAC) coding was implemented. Minimum sequencing coverage of 100 reads was required to record a nucleotide call at each genome position.

Amino-acid mutation matrix was generated using an analogous strategy requiring a minimum sequencing coverage spanning the entire codon length. Because the HBV genome contains overlapping reading frames, each open reading frame was covered independently of others.

#### Phylogenetic tree

The Woolly Monkey HBV (GenBank: NC_028129.1) nucleotide sequence was used as an outgroup for the construction of the phylogenetic tree. The Woolly Monkey sequences were aligned to the HMM profile built by the multiple-sequence alignment of HBV nucleotide sequences from our study using hmmbuild of HMMER software (http://hmmer.org/) v.3.2.1. The maximum-likelihood-based phylogenetic tree was inferred using the IQ-TREE software (http://www.iqtree.org/) v.1.16.12,^49^ where the best model within the general time-reversible (GTR) model family was selected as the nucleotide substitution model. Branch support bootstrap values were calculated using UFBoot^50^ with 1,000 replicates. In general, HBV genotypes were monophyletic, and the tree topology corresponded to our current understanding of evolutionary relationships between HBV genotypes,^51^ suggesting the validity of the inferred phylogeny (Figure S2).

#### Phylogenetic PCs

To capture HBV amino-acid variations that are correlated with phylogenetic structure and to summarize such variation as synthetic variables, phylogenetic PCA (pPCA) was applied to HBV amino-acid dosages (presence or absence). Similar to PCA, pPCA is a dimensional reduction technique where orthogonal synthetic variables that capture most of the variance are constructed. However, unlike standard PCs, pPCs applied to amino acids are constrained such that variations that are correlated with the phylogenetic structures are captured. The ppca function within the *adephylo* package (https://cran.r-project.org/web/packages/adephylo/index.html) v.1.1–11 in R, which implements the pPCA formulation as described by Jombart et al.,^52^ was utilized. Abouheif’s proximity was selected as the phylogenetic proximity metric. Because pPCs are constrained by phylogenetic structures, they were able to accurately discern HBV genotypes as expected (Figure S3).

### Association studies

For G2G association studies, our analyses were restricted to common human SNPs (minor allele frequency >0.05) and common HBV amino-acid mutations (minor allele count >20) for which the model was able to reach convergence. Analyses were conducted separately in the East Asian and European cohort to avoid spurious associations driven by population stratification given that HBV genotypes and variation are highly correlated with patient ancestry (Table 1). This resulted in the inclusion of 164,204 human SNPs with 442 HBV amino-acid mutations in the East Asian cohort and 196,545 human SNPs with 88 HBV amino-acid mutations in the European cohort. HBV amino-acid mutations with perfect correlation (r^2^ = 1), which include mutations at the same amino-acid position or mutations in overlapping reading frames driven by the same nucleotide mutations, were pruned. The number of effective tests ($N_{eff}$) was determined to be 433 and 88 in the East Asian and European cohort, respectively. To account for multiple testing, the Bonferroni correction threshold was derived by dividing the genome-wide significance threshold (5× $10^{-8}$) by the total number of effective tests ($N_{eff}=521$), resulting in a threshold of 9.6× $10^{-11}$.

**Table 1:** Baseline characteristics

	**East Asian (*N* = 424)**	**European (*N* = 143)**
**Age**		

Median (range)	42 (18–69)	38 (18–73)

**Sex**		

Female	157 (37%)	47 (32.9%)
Male	267 (63%)	96 (67.1%)

**HBV genotype**		

A	7 (1.65%)	24 (16.8%)
C	310 (73.1%)	3 (2.1%)
D	5 (1.18%)	111 (77.6%)
F	0 (0%)	3 (2.1%)
H	0 (0%)	2 (1.4%)
B	102 (24.1%)	0 (0%)

**HBeAg**		

Negative	170 (40.1%)	73 (51%)
Positive	254 (59.9%)	70 (49%)

Baseline characteristics and HBV genotypes of study participants, grouped according to genetically determined ancestry.

Each retained HBV amino-acid mutation was encoded as binary outcomes, representing either presence above 15% in the intra-host population or absence. A GWAS was conducted for each HBV amino-acid variant as the outcome using the generalized linear mixed model implemented by SAIGE (v.0.35.8)^53^ in R. Age, sex, the top four human PCs (8.3% of variance explained), and the top six pathogen pPCs (87.8% of variance explained) were included as covariates. *p* values based on the saddle point approximation (SPA) test implemented by SAIGE software (https://saigegit.github.io/SAIGE-doc/) v.0.35.8^53^ are reported. For associations involving rs2296651, due to the unreliability of effect-size estimates based on the SPA test for variants with low minor allele counts, odds ratios (ORs) were calculated using Firth logistic regression implemented by PLINK v.1.9.

To confirm the absence of genomic inflation in the GWAS with significant genome-wide association(s), we constructed quantile-quantile (QQ) plots and calculated genomic inflation factors (λ), which demonstrated the absence of inflation (Figures S4 and S5).

For G2G associations involving HLA amino-acid mutations, which could be multiallelic, the GWASs were based on omnibus tests where the --hap-snps and --chap flag of PLINK (https://zzz.bwh.harvard.edu/plink/) v.1.07 was used to group amino-acid residues at the same position. For associations involving HLA alleles, the SPA test implemented by SAIGE was used to calculate the *p* values and ORs under a dominant model. For the conditional analyses, the top association (either SNP, amino acid, or allele) was conditioned on using the --cond command of PLINK v.1.9.

Inverse normal transformations were applied to biomarkers of HBV infection (HBsAg levels, serum ALT levels, and viral load). HBV nucleotide mutations associated with biomarkers of HBV infection were identified using linear regression, adjusting for HBeAg status, age, sex, the top four human PCs, and the top six pathogen pPCs. HBV nucleotide mutations associated with HBeAg status were identified using logistic regression, adjusting for age, sex, the top four human PCs, and the top six pathogen pPCs.

For stratified analyses involving rs2296651 genotype and associated HBV preS1 escape haplotypes, individuals were separated into groups according to human genotype and the presence or absence of HBV escape haplotypes. Pairwise comparisons were made between groups. Using multiple linear regression, each biomarker of HBV infection was regressed against the group indicator (binary 0 or 1), HBeAg status, age, sex, the top four human PCs, the top six pathogen pPCs, and all nucleotide mutations significantly associated with each marker of infection (Figure S6). Nucleotide mutations significantly associated with each marker of infection were clumped (r^2^ < 0.8) based on the strength of association, such that only independent mutations were incorporated in the regression model.

### *In silico* model of preS1-NTCP binding

To construct an *in silico* model of preS1-NTCP binding, the ColabFold^54^ (https://colab.research.google.com/github/sokrypton/ColabFold/blob/main/AlphaFold2.ipynb) implementation of AlphaFold-Multimer was used. The developers of AlphaFold-Multimer have shown that the method has the ability to predict protein-peptide complexes when the protein and peptide are encoded as separate chains.^55^ Here, we encoded the preS1-derived peptide (amino acids 2–60 of preS1) and NTCP protein as separate chains. We relied on an *in silico* approach because existing cryoelectron microscopy (cryo-EM) data^56^ did not offer sufficient resolution to model the interface. Nevertheless, the cryo-EM structure of NTCP in the presence of preS1 (PDB: 7VAG) was used as a template to improve the *in silico* predictions. Default settings of ColabFold were used, and the number of recycles was set at three. Images of predicted structures were produced using the UCSF Chimera package (https://www.cgl.ucsf.edu/chimera/)^57^ from the Resource for Biocomputing, Visualization, and Informatics at the University of California, San Francisco.

The amino-acid sequence of NTCP that was used was identical to that of the structure (PDB: 7VAG) excluding missing residues. The preS1 sequence was based on the genotype C consensus sequences derived from the cohort, stratified based on NTCP genotype (NTCP wild type [WT] or S267F).

### PreS1 haplotype calling and intra-host nucleotide diversity

To assemble sequencing reads into intra-host preS1 haplotypes, the shorah software (https://github.com/cbg-ethz/shorah)^58^ was used. The shotgun-based local analysis was selected because the region of interest (first 177 bp of preS1) was slightly longer than the average read length (150 bp). Low-confidence haplotypes (posterior <0.95 or supporting reads <10) were filtered out. Nucleotide haplotypes were translated into amino-acid sequences using the seqinr package (https://cran.r-project.org/web/packages/seqinr/index.html)^59^ in R. A multiple-sequence alignment with 1,000 sequences was constructed for each patient by up-sampling each haplotype based on its intra-host proportion. Sequence logos that represent the overall intra-host composition across all samples were then constructed based on merging multiple-sequence alignment across all samples and using the ggseqlogo package (https://omarwagih.github.io/ggseqlogo/)^60^ in R.

To estimate the magnitude and infer the type of intra-host selection occurring within preS1, we calculated the ratio of non-synonymous to the synonymous nucleotide diversity ($\pi_{N}/\;\pi_{S}$). The $\pi_{N}/\;\pi_{S}$ ratio is analogous to the $d_{N}/\;d_{S}$ ratio but is weighted according to the composition of intra-host viral population.^61^ To incorporate the effect of overlapping reading frames, the mean pairwise number of substitutions per site that are non-synonymous in both frames ($\pi_{NN}$) and that are synonymous in preS1 but non-synonymous in polymerase ($\pi_{SN}$) were calculated using OLGenie (https://github.com/chasewnelson/OLGenie)^62^ based on the up-sampled multiple-sequence alignment. Non-synonymous ($\pi_{N}$) and synonymous nucleotide ($\pi_{S}$) diversity ignoring the overlapping reading frame was also calculated using the same approach.

### Population frequency of HBV preS1 amino-acid mutations

To estimate the population frequencies of HBV preS1 amino-acid mutations, amino-acid multiple-sequence alignments for the large surface protein were downloaded separately for each HBV genotype (A, B, C, D) from HBVdb.ibcp.fr.^48^ Positions were normalized to genotype C (NCBI: GQ924620). The Entrez batch query tool (https://www.ncbi.nlm.nih.gov/sites/batchentrez) was used to extract the “country” field from the metadata for each HBV sequence hosted on the NCBI Nucleotide Database (https://www.ncbi.nlm.nih.gov/nucleotide/). Countries were subsequently grouped into geographical regions.

### In vitro preS1-NTCP binding assays

We synthesized the 59-amino-acid myristoylated peptides derived from the most common genotype C preS1 haplotypes observed in either NTCP WT carriers or NTCP S267F carriers:

NTCP S267F (Myr-ARP):

Myr-MGGWSSKPRKGMGTNL**A**VPNPLGFFPDHQLDLAF**R**ANSNNPDWDFNPNKD**P**WPEANQVG

NTCP WT (Myr-WT):

Myr-MGGWSSKPRQGMGTNL**S**VPNPLGFFPDHQLDPAF**G**ANSNNPDWDFNPNKD**H**WPEANQVG

The variable amino acids at positions 17, 35, and 51 of the preS1 region are highlighted in bold. For binding assay experiments, HepG2, HepG2 HA-NTCP WT, and HepG2 HA-NTCP S267F were aliquoted at a concentration of 2 × 10^5^ per Eppendorf tube. The cells were pelleted for 3 min at 100 × g, and the pellet was resuspended in appropriate peptide dilution. Peptides were applied at 0, 15, 50, 200, and 1,000 nM, and Bulevirtide (Myrcludex B) was added as side binding control. Cells were incubated at room temperature for 30 min in the dark and then pelleted by centrifugation for 3 min at 100 × g and washed five times with PBS. The washed cells were resuspended in 500 μL of FACS buffer (0.5% BSA and 0.02% sodium azide in 1× PBS), and fluorescence was measured by flow cytometry using Cell Sorter BD FACS Celesta. For immunofluorescence readout of the binding assay, cells were seeded in 24-well plate (3 × 10^5^/well) and 24 h later incubated with peptides as performed for fluorescence-activated cell sorting (FACS) analysis. After incubation, cells were washed with 2% BSA/PBS and fixed with 1.25% paraformaldehyde. Atto 565 signal was detected at fluorescence microscope. Further details can be found in Supplementary Methods S2.

### HLA peptide-binding predictions

To predict peptides restricted by specific HLA class I alleles and their respective binding affinities or elution probabilities, the NetMHCpan4.0 (http://tools.iedb.org/mhci/download/)^63^ and MixMHCpred2.2 (https://github.com/GfellerLab/MixMHCpred)^64^ software were used.

For each HBV amino-acid position associated with a human HLA allele, we obtained flanking sequences based on the HBV genotype C reference sequence (NCBI: GQ924620). Next, for each HBV amino-acid position and HLA-allele pair, we used a sliding window approach where we predicted the binding affinities for all possible 9- to 14-mers peptides to identify the most likely peptide restricted by the HLA allele. We chose to include all 9–14 mers as they are the most likely peptide lengths for the HLA class I alleles of interest (Figure S7). For each HLA allele and HBV variant pair, the peptide with the lowest NetMHCpan predicted elution percentile rank was chosen as the putative peptide.

## Results

### Study description

Table 1 summarizes the baseline characteristics of the study participants. Based on the consensus between self-reported and genetically determined ancestry (Figure S1), we separated our study into an East Asian cohort (*N* = 424) and a European cohort (*N* = 143). Individuals within the East Asian cohort were predominantly infected with HBV genotype B (*N* = 310; 73%) and C (*N* = 102; 24%), while those within European cohort were predominantly infected with HBV genotype A (*N* = 111; 78%) and D (*N* = 24; 17%). Both HBeAg-positive (*N* = 243; 43%) and HBeAg-negative (*N* = 324; 57%) participants were included in the study.

### Association study between human and HBV genetic variation

To identify associations between human SNPs and HBV amino-acid mutations, which we refer to as G2G associations, we conducted multiple GWASs where each HBV amino-acid variant was encoded as a binary outcome (presence above 15% in the intra-host viral population or absence). The GWASs were conducted separately in two cohorts, including participants with East Asian and European ancestry, respectively. Human and pPCs were included as covariates to correct for population stratification.

**Figure 1:**
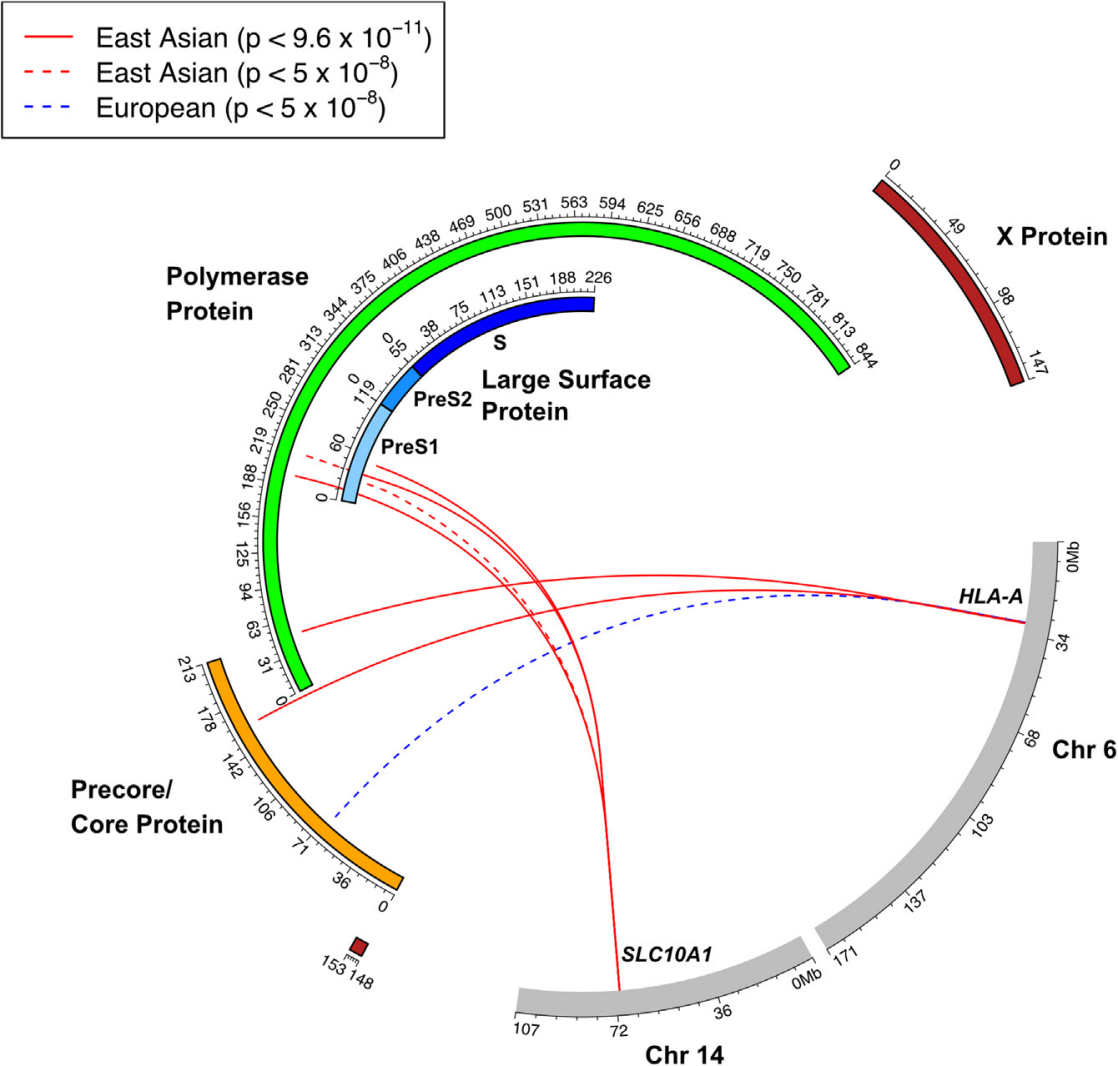
Genome-to-genome analysis Associations between human SNPs and HBV amino-acid mutations. Gray panels represent human chromosomes and respective nucleotide positions. Colored panels represent HBV proteins and respective amino-acid positions. Solid lines represent significant associations under Bonferroni correction (p < $9.6\times 10^{-11}$), and dashed lines represent genome-wide significant associations (p < $5.0\times 10^{-8}$). Red lines represent associations within the East Asian cohort, and blue lines represent associations within the European cohort.

We found that variants mapping to two regions of the human genome were significantly associated with HBV amino-acid variation: the *SLC10A1* gene region on chromosome 14 and the HLA class I region on chromosome 6. (Figure 1).

On chromosome 14, an SNP in the *SLC10A1* (MIM: 182396) gene (rs2296651), which encodes for an amino-acid change (S267F) on the HBV entry receptor NTCP, showed significant association with three amino-acid mutations within the preS1 domain of the HBV large-surface protein (Table 2). According the gnomAD, rs2296651 is a variant that is prevalent in East Asian populations but virtually absent in all others. Correspondingly, the associations were only observed in the East Asian cohort. The strongest association was between rs2296651 and position 17 of preS1 (associated amino acid: A; p = $1.7\times 10^{-12}$; OR = 179.2), along with other significant associations with position 35 (associated amino acid: R; p = $3.8\times 10^{-11}$ ; OR = 36.5) and position 51 (associated amino acid: P; p = $1.9\times 10^{-12}$; OR = 88.9). A suggestive association was observed between rs2296651 and the position 32 of preS1 (associated amino acid: L; p = $1.5\times 10^{-10}$; OR = 126.7). To confirm that the significant associations were not genotype specific, we conducted a stratified analysis within HBV genotypes B and C in the East Asian cohort (Table 2). Genotypes A and D were excluded given their low prevalence in the East Asian cohort. The direction of associations was identical across genotypes, and effect sizes were similar across genotypes for position 17 and position 35 while less strong for position 51 in genotype B. Due to the overlapping reading frames of HBV, rs2296651 also showed significant association with position 197 of the polymerase protein (associated amino acid: C; p = $6.9\times 10^{-12}$; OR = 181.8) and suggestive association with position 215 of the polymerase protein (associated amino acid = Q; p = $2.8\times 10^{-9}$ ; OR = 27.5). The associated polymerase mutations reflect the same variation at the nucleotide level as the associated preS1 mutations.

**Table 2:** Association between rs2296651 and HBV preS1 mutations in the East Asian cohort

**preS1** **Position**	**Cases: associated amino acid (%)**	**Controls: ALT** **amino acid**	**rs2296651 MAF (controls:cases)**	**p**	**OR**	**OR 95% CI**
**All HBV genotypes**						

17	A (5%)	S	0.031:0.48	1.69 × 10^−12^	179.2	30.4–1055.4
35	R (9.9%)	E, G, K	0.025:0.31	3.83 × 10^−^^11^	36.5	12.4–107.3
51	P (6.6%)	H, K, N, Q, R, T	0.025:0.45	1.87 × 10^−^^12^	88.9	23.4–336.9

**HBV genotype C**						

17	A (5.1%)	S	0.029:0.47	5.94 × 10^−^^8^	118.6	16.7–842.3
35	R (11.9%)	E, G, K	0.016:0.31	5.61 × 10^−^^10^	28.9	9.1–91
51	P (8.7%)	H, Q	0.014:0.44	6.74 × 10^−13^	78.7	18.8–329.4

**HBV genotype B**						

17	A (4.9%)	S	0.031:0.5	2.12 × 10^−3^	53.0	5.1–551.4
35	R (2.9%)	K	0.04:0.5	NA	25.5	2.6–249.9
51	P (1%)	K, N, T	0.05:0.5	NA	5.1	0.5–48.1

Samples with the presence (exceeding intra-host frequency of 15%) and absence of the associated HBV amino acid were encoded as cases and controls, respectively. Minor-allele frequency (MAF) ratio represents the MAF of rs2296651 in the cases compared to controls. Odds ratio (OR) is based on firth logistic regression assuming an additive model based on the minor allele (A) of rs2296651. *p* value is based on the SPA test, with NA indicating that the algorithm failed to converge due to low minor allele count.

**Figure 2:**
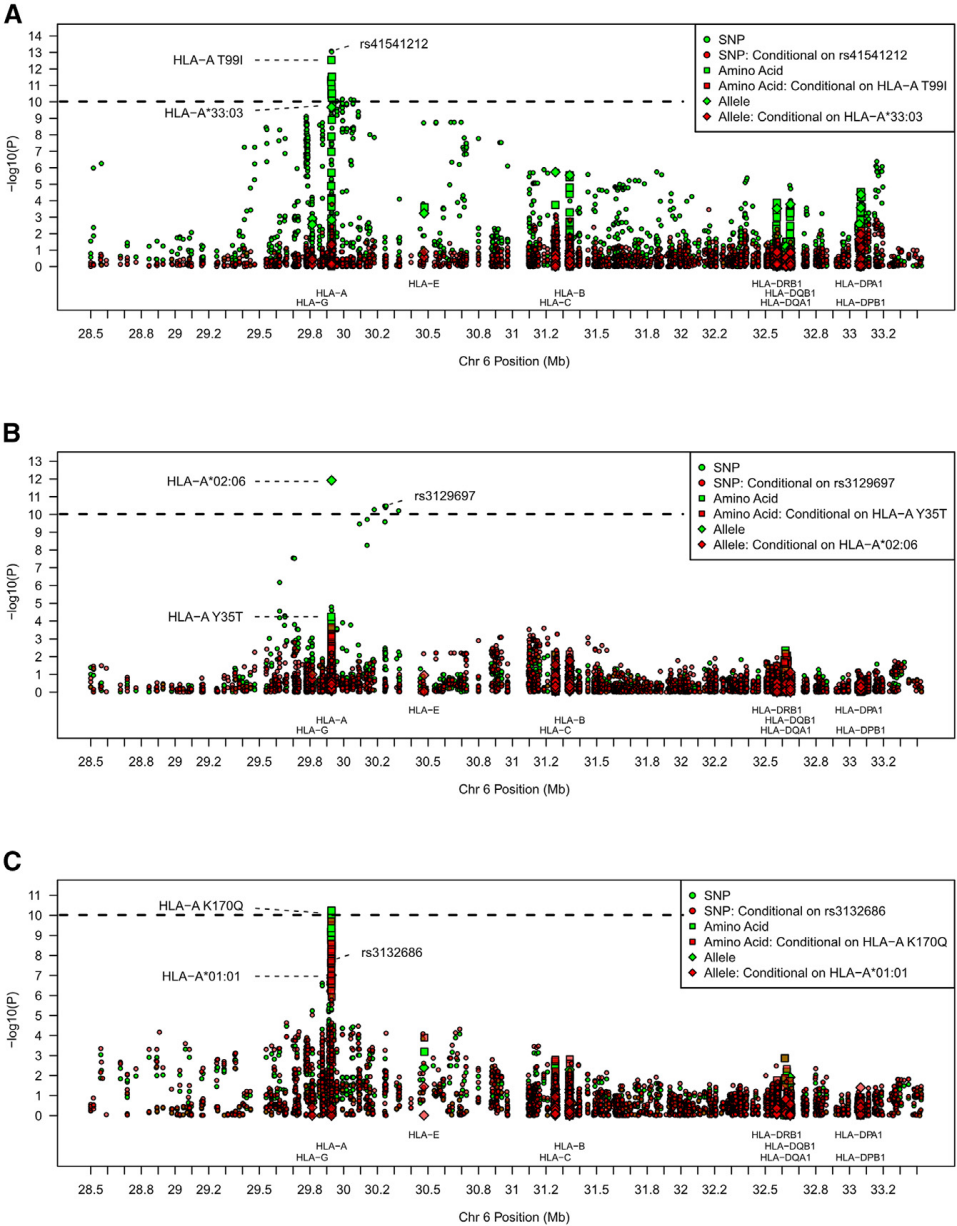
Associations between human variants within the HLA region and HBV amino-acid mutations Shapes represent the type of human variant (SNP, amino-acid substitution, or HLA allele). Red points represent conditional associations based on the strongest association. Dashed lines represent Bonferroni corrected threshold. (A) Association with position 160 (associated amino acid: A) of HBV precore/core protein in the East Asian cohort. (B) Association with position 49 (associated amino acid: N) of HBV polymerase protein in the East Asian cohort. (C) Association with position 67 (associated amino acid: Y) of HBV precore/core protein in the European cohort.

On chromosome 6, SNPs mapped to the *HLA-A* (MIM: 142800) gene within the HLA class I region showed significant associations (top association: *p* = $8.4\times 10^{-14}$) with two HBV amino-acid mutations in the East Asian cohort (Figures 2A and 2B) and suggestive associations (top association: *p* = $1.9\times 10^{-8}$) with an HBV amino-acid variant in the European cohort (Figure 2C). Because the associated SNPs could be tagging functional variants at the HLA amino-acid or allele level, we imputed the four-digit HLA alleles and HLA amino acids, which we used in additional association analyses (Figure 2; Tables S2–S5). In the East Asian cohort, HLA-A T99I (*p* = $2.9\times 10^{-13}$ ; OR = 0.07) and HLA-A^∗^33:03 (*p* = $2.1\times 10^{-10}$; OR = 0.08) were associated with position 160 of the HBV precore/core protein (associated residue: A). In addition, HLA-A Y35T (*p* = $5.8\times 10^{-5}$; OR = 0.33) and HLA-A^∗^02:06 (*p* = $1.3\times 10^{-12}$; OR = 84.2) were associated with position 49 of the HBV Pol (associated residue: N). In the European cohort, HLA-A K170Q (*p* = $5.9\times 10^{-11}$; OR = 2.95) and HLA-A^∗^01:01 (*p* = $9.7\times 10^{-8}$; OR = 0.07) were associated with position 67 of HBV precore/core protein (associated residue: Y). To evaluate whether a separate signal might be present outside of the *HLA-A* gene, we conducted conditional analyses based on the top association within *HLA-A* (Figure 2). We did not observe any significant associations outside of *HLA-A* in these conditional analyses. Given that HLA-allele frequencies are highly variable among human populations, we also investigated whether the associations were restricted to certain ancestry subgroups within the East Asian and European cohorts. PCA revealed that the HLA alleles and the associated HBV mutations were present in multiple ancestry subgroups within the East Asian or European cohort (Figures S8 and S9), and their prevalences were not strongly correlated with any human or phylogenetic PCs (r^2^ < 0.05) (Figure S10).

### NTCP and HBV preS1 variations

Because it has been established that the interaction between the receptor-binding region of the HBV preS1 and NTCP is essential for viral entry,^65^^,^^66^ we used AlphaFold-Multimer^55^ to construct *in silico* models of binding that allowed us to evaluate the proximity between the human NTCP variant (rs2296651, NTCP S267F) and associated HBV preS1 mutations when bound. We first constructed a model based on the human WT NTCP and a preS1 peptide (amino acids 2–60) derived from the HBV genotype C consensus sequence. Consistent with cryo-EM densities, the top-ranking model indicated binding of the preS1 peptide to the extracellular opening of the NTCP tunnel that is also responsible for substrate transport (Figures S11A and S11B). According to the *in silico* model, the NTCP S267 variant is located within the tunnel and is in proximity to S17 and G35 of preS1 when bound (Figure S11A). S17 of preS1 is the most proximal residue to S267 of NTCP at 9.5 Å. G35 of preS1 is 16.2 Å to S267 of NTCP. H51 of preS1 is 9.4 Å to patch 1 of NTCP (84–87), which has been reported to include residues essential for preS1 binding.^67^ To study the impact of the NTCP S267F on the binding of preS1 peptides that encode or do not encode the mutations associated with the human NTCP variant, we also constructed separate models for every combination (Supplementary Methods S1, Table S6). Given that the prediction confidence for both the preS1-NTCP interface (Figure S12A) and the preS1 peptide structure itself (Figure S12B) was relatively low, conclusions that could be drawn from this *in silico* analysis were limited.

**Figure 3:**
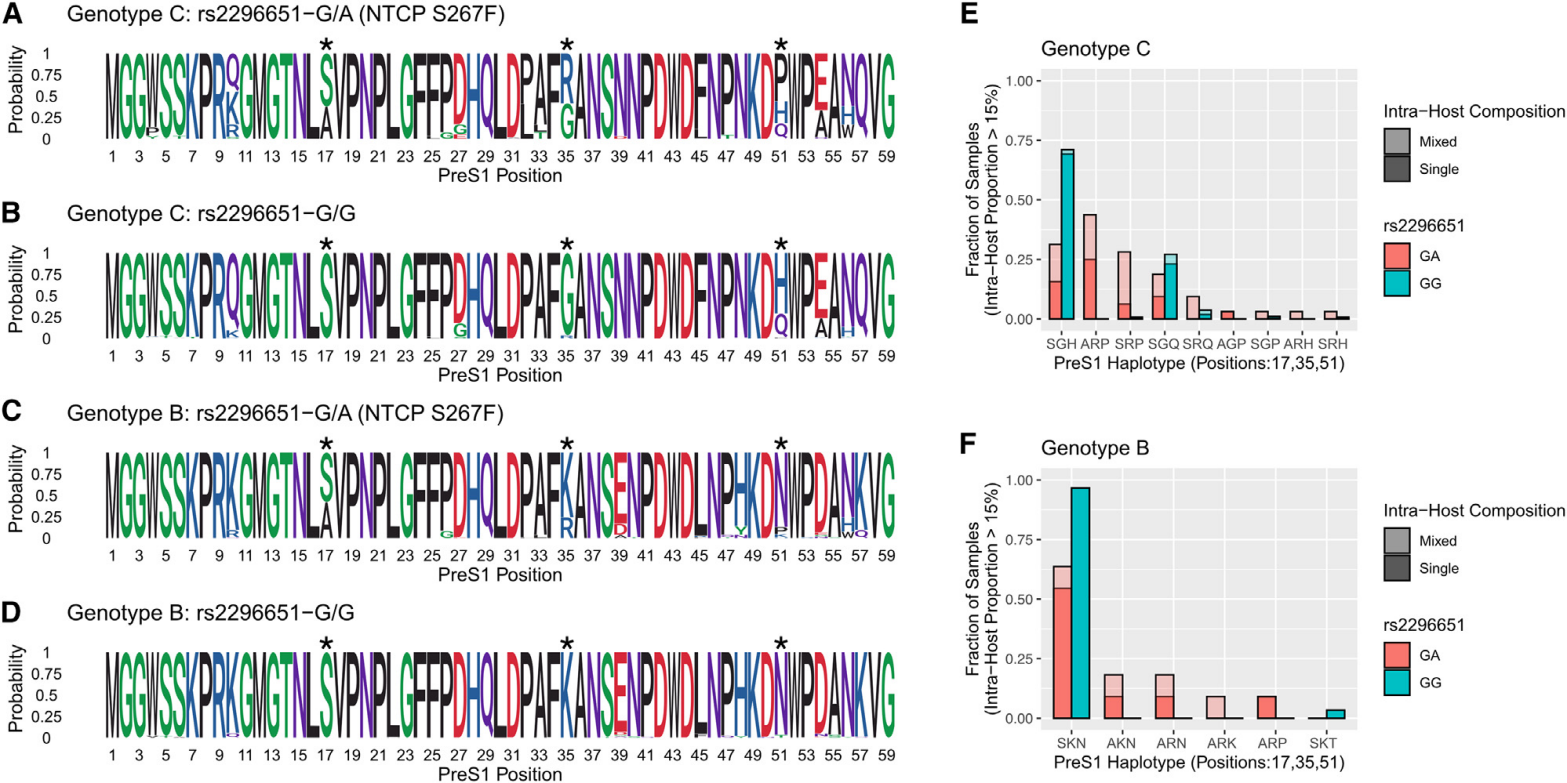
Intra-host composition of preS1 receptor-binding region haplotypes Sequence logos represent the overall intra-host composition of preS1 receptor-binding region sites. PreS1 sites that show significant association with rs2296651 (NTCP S267F) are marked with asterisks. Bar plots represent the fraction of samples where the haplotype (defined based on the 17^th^, 35^th^, and 51^st^ residue of preS1) exceeded the minimal intra-host frequency of 15%. Colors represent rs2296651 genotype. Samples with multiple intra-host haplotypes are considered as mixed. (A and B) Sequence logo of genotype C-infected individuals who carry rs2296651-G/A and rs2296651-G/G, respectively. (C and D) Sequence logo of genotype B-infected individuals who carry rs2296651-G/A and rs2296651-G/G, respectively. (E and F) Frequency of intra-host haplotypes in genotype C- and genotype B-infected individuals, respectively.

To characterize the effect of the human NTCP variant on the intra-host diversity of the preS1 receptor-binding region, we stratified patients according to rs2296651 genotypes and constructed sequence logos that represent the overall intra-host composition. Figures 3A and 3B illustrate the intra-host composition in genotype C-infected individuals who are heterozygous NTCP S267F carriers (rs2296651-G/A) and NTCP WT carriers (rs2296651-G/G), respectively. Figures 3C and 3D illustrate the intra-host composition in genotype B-infected individuals who are heterozygous NTCP S267F carriers (rs2296651-G/A) and NTCP WT carriers (rs2296651-G/G), respectively. For both HBV genotypes, we observed that preS1 sites associated with rs2296651 (marked by asterisks) were highly conserved in rs2296651-G/G individuals but showed increased diversity in rs2296651-G/A individuals, suggesting intra-host selection induced by the NTCP variant. To quantify the magnitude and direction of intra-host selection within the entire preS1 receptor-binding region, we compared the non-synonymous nucleotide diversity ($\pi_{N}$) against the synonymous nucleotide diversity ($\pi_{S}$) in rs2296651-G/A and rs2296651-G/G individuals. Compared to the $d_{N}/d_{S}$ ratio, the $\pi_{N}/\pi_{S}$ ratio is weighted according to the intra-host composition of viral populations but similarly reflects positive selection if greater than 1, neutral selection if equal to 1, and purifying selection if less than 1.^61^ No significant differences were observed in $\pi_{S}$ between rs2296651-G/G and rs2296651-G/A individuals, but higher $\pi_{N}$ was observed in rs2296651-G/A carriers (Figure S13A). This contributed to a higher $\pi_{N}/\pi_{S}$ ratio in rs2296651-G/A individuals, indicating increased positive selection. We observed similar results when we controlled for the effect of the overlapping reading frames by restricting to only substitutions and sites that are non-synonymous in the polymerase protein (Figure S13B).

Because the HBV preS1 sites that are associated with the human NTCP variant are in high linkage disequilibrium, we next assembled ultra-deep HBV sequencing reads (average coverage of 6,045× in the receptor-binding region) into intra-host haplotypes. We grouped haplotypes according to residues at the preS1 positions (17, 35, and 51) that are associated with NTCP S267F. Figures 3E and 3F show the fraction of samples for which each haplotype is present (exceeding intra-host frequency of 15%) within genotype C- and B-infected individuals, respectively. We identified putative escape haplotypes that are enriched in heterozygous NTCP S267F (rs2296651-G/A) carriers compared to homozygous WT (rs2296651-G/G) carriers (e.g., ARP in genotype C; AKN/ARN in genotype B). Furthermore, we observed that a higher proportion of heterozygous carriers have multiple intra-host preS1 haplotypes compared to homozygous WT carriers, consistent with the presence of intra-host selective pressure. Figure S14 shows that some heterozygous carriers are infected with mixtures of escape haplotypes (e.g., ARP in genotype C; AKN/ARN in genotype B) and population-consensus haplotypes (e.g., SGH/SGQ in genotype C; SKN in genotype B). For some heterozygous carriers, we were also able to establish possible evolutionary trajectories from the population-consensus haplotypes to the escape haplotypes (Figure S15).

Given that the HBV preS1 mutations associated with the human NTCP variant likely reflect viral escape, it is expected that they would be more prevalent in geographical regions where the NTCP variant is also prevalent. According to gnomAD,^66^ the minor allele frequency of rs2296651 (NTCP S267F) is approximately 8% in East Asians and virtually absent in all other populations. Corresponding to the prevalence of the human variant, the HBV mutation most strongly associated with NTCP S267F (preS1 S17A) is predominantly found in genotype B and C HBV samples from East Asian and Southeast Asian countries according to HBVdb^48^ (Table S7).

Finally, we evaluated the effect of the NTCP variant and associated preS1 escape haplotypes on biomarkers of HBV infection. We stratified patients according to rs2296651 genotypes and the presence or absence of escape haplotypes (Table 3). Compared to the NTCP WT group, the NTCP S267F non-escape group had lower levels of serum ALT levels (β=−0.22, p = $4.4\times 10^{-4}$) but no significant differences in HBsAg levels (p = 0.55) and viral load (p = 0.14). Compared to either the NTCP S267F non-escape group or the NTCP WT group, the NTCP S267F escape group had no significant differences in any of the biomarkers. However, the statistical power of this comparison is limited, given the small sample size of the NTCP S267F escape group (*N* = 18).

**Table 3:** Impact of rs2296651 and associated preS1 haplotypes on biomarkers of HBV infection

**Biomarkers**	**NTCP WT (*N* = 343) rs2296651:G/G preS1: non-escape**	**NTCP S267F non-escape (*N* = 23) rs2296651: G/A preS1: non-escape**			**NTCP S267F escape (*N* = 18) rs2296651: G/A preS1:escape**				
	**Mean (SD)**	**Mean (SD)**	**vs. NTCP WT**		**Mean (SD)**	**vs. NTCP S267F** **non-escape**		**vs. NTCP WT**	
			**Beta (SE)**	**p**		**Beta (SE)**	**p**	**Beta (SE)**	**p**
Viral load (log_10_ U/L)	6.75 (1.63)	6.82 (1.19)	−0.32 (0.21)	0.14	6.74 (1.17)	−0.06 (0.29)	0.83	−0.35 (0.24)	0.15
Serum ALT (log_10_ IU/ml)	1.88 (0.31)	1.74 (0.20)	−0.22 (0.06)	4.4e−4	1.78 (0.22)	0.15 (0.06)	0.02	−0.13 (0.07)	0.08
HBsAg level (log_10_ IU/ml)	3.65 (0.80)	3.63 (0.63)	−0.08 (0.14)	0.55	3.39 (0.46)	−0.30 (0.22)	0.18	−0.33 (0.16)	0.04

The homozygous NTCP WT group represents individuals who carry rs2296651-G/G and are infected with non-escape haplotypes. The heterozygous NTCP S267F non-escape group represents individuals who carry rs2296651-G/A and are infected with non-escape haplotypes. The heterozygous NTCP S267F escape group represents individuals who carry rs2296651-G/A and are infected with escape haplotypes. Escape haplotypes were defined as HBV preS1 haplotypes that are absent in all rs2296651-G/G individuals but present in any rs2296651-G/A individuals. *p* values and beta obtained from multiple linear regression adjusted for age, sex, HBeAg status, the top four human PCs, and the top six pathogen pPCs, and HBV nucleotide mutations significantly associated with the respective biomarker (Figure S10).

### *In vitro* preS1-NTCP binding assays

**Figure 4:**
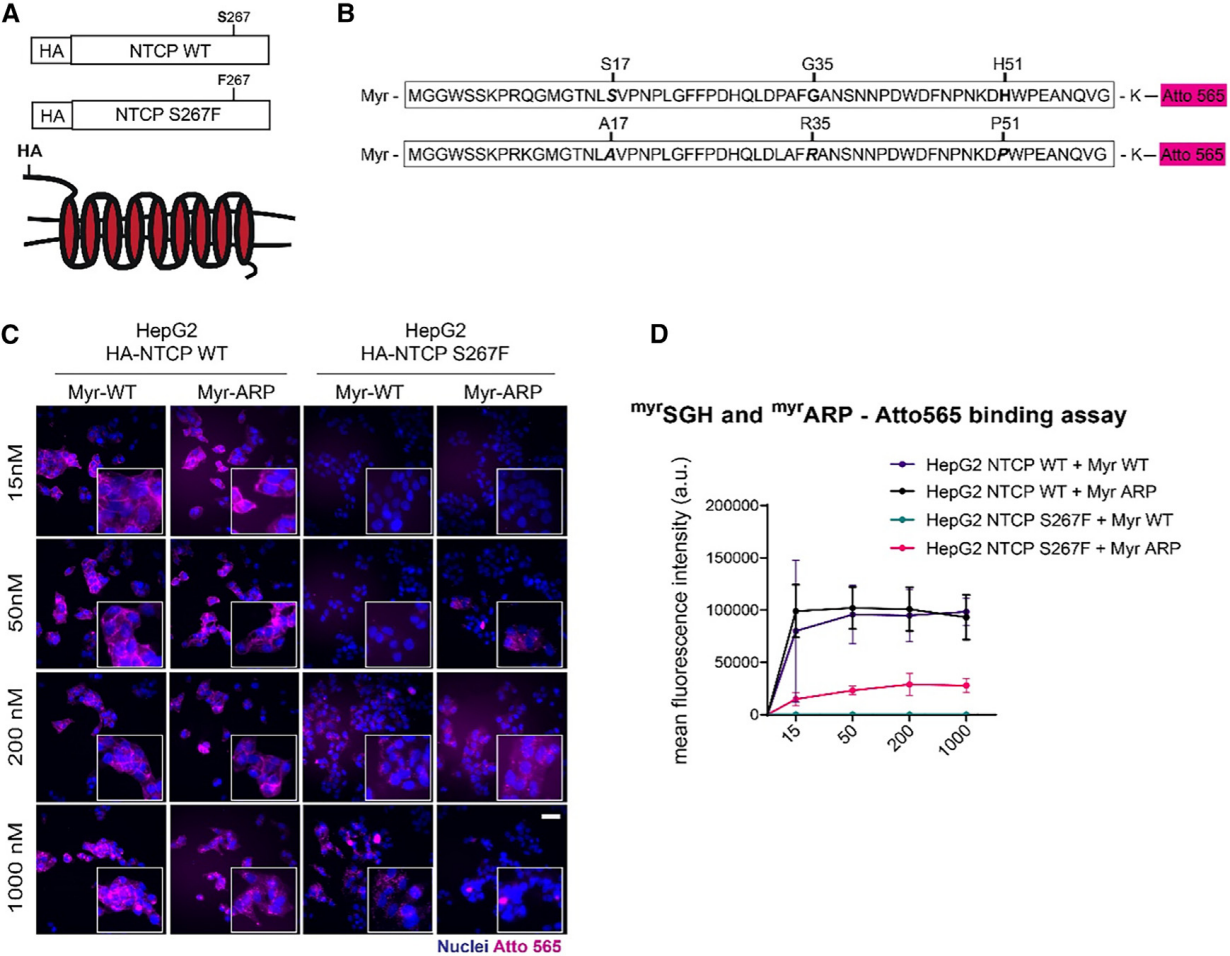
Synthesis of myristoylated peptides for affinity-binding studies in HepG2 HA-NTCP WT and HA-NTCP S267F cells (A) HepG2 cells were transduced with lentivirus for the stable expression of NTCP WT or NTCP S267F with an HA tag at the N terminus. (B) Schematic representation of WT and ARP myristoylated peptides synthesized and linked to Atto565-maleimide via addition of a lysine (K) at the C terminus. (C and D) For binding affinity assays HepG2, HepG2 HA-NTCP WT, and HepG2 HA-NTCP S267F, cells were incubated at increasing concentration (15, 50, 200, or 1,000 nM) of Myr-WT and Myr-ARP peptides. Atto565 signal was detected by fluorescence microscopy (scale bar: 100 μm), (D) or mean fluorescence intensity was measured by flow cytometry and plotted against the respective peptide concentration. Data were normalized by subtracting the autofluorescence at the respective concentration given by the binding in HepG2 cells.

To elucidate the effect of identified preS1 mutations on binding to NTCP, we generated HepG2 cell lines stably expressing NTCP WT or NTCP S267F, both with a hemagglutinin (HA) tag at the N terminus (Figure 4A). As expected, taurocholate uptake performed in HepG2 transduced with HA-NTCP WT or HA-NTCP S267F revealed a reduced functionality of the NTCP variant compared to WT NTCP.^68^ HepG2 HA-NTCP WT had a comparable level of taurocholate uptake to a well-established HepG2 NTCP clone (A3),^69^ indicating that the introduction of an HA tag does not impair NTCP functionality (Figure S16A). To exclude differences in intracellular and membrane expression between HA-NTCP WT and HA-NTCP S267F, we performed immunofluorescence staining using HA tag antibody and quantification of NTCP mRNA expression via RT-qPCR. Both methodologies revealed a comparable surface and intracellular expression level between NTCP WT and NTCP S267F (Figures S16B and S16C). We then synthesized myristoylated peptides derived from the most common genotype C preS1 haplotype found in NTCP WT carriers (referred to as Myr-WT) and in NTCP S267F carriers (referred to as Myr-ARP, carrying preS1 mutations S17A, G35R, H51P) (Figure 4B). We first assessed their binding capacity to NTCP WT, without (HepG2 NTCP A3 clone), and with HA-tag (HepG2 HA-NTCP WT), indicating no binding impairment when HA is present (Figure S16D). We then performed binding assays to measure their binding affinities to both HA-NTCP WT or HA-NTCP S267F. Myr-ARP and Myr-WT were able to bind NTCP WT with similar affinity (Figures 4C and 4D). Compared to the diminished binding capacity observed between Myr-WT and NTCP S267F, only a partial increase in binding affinity was observed between Myr-ARP and NTCP S267F (Figures 4C and 4D).

### HLA class I variations and HBV epitopes

We hypothesized that the associations observed between HLA class I variation and HBV amino-acid mutations might be driven by immune evasion. An individual’s HLA class I alleles determine the repertoire of viral epitopes that can be recognized and restricted by CTLs. Depending on the HLA alleles that an individual carries, a selective advantage could be present for specific HBV mutations that alter epitope sequences and lower HLA-binding affinities.

To test this hypothesis, we first identified whether HBV amino-acid mutations associated with HLA-A variation overlap with predicted viral epitopes. We used a sliding window approach to generate binding predictions of the HLA allele against all possible 9- to 14-mer peptides that overlap with the position based on two HLA-binding prediction algorithms (NetMHCpan^63^ and MixMHCpred^64^). We observed that all three HLA-associated HBV positions are located within at least one epitope predicted to be strongly bound (elution percentile rank <0.5% and half maximal inhibitory concentration [IC50] < 50 nM) by the respective HLA allele (Table S8).

**Figure 5:**
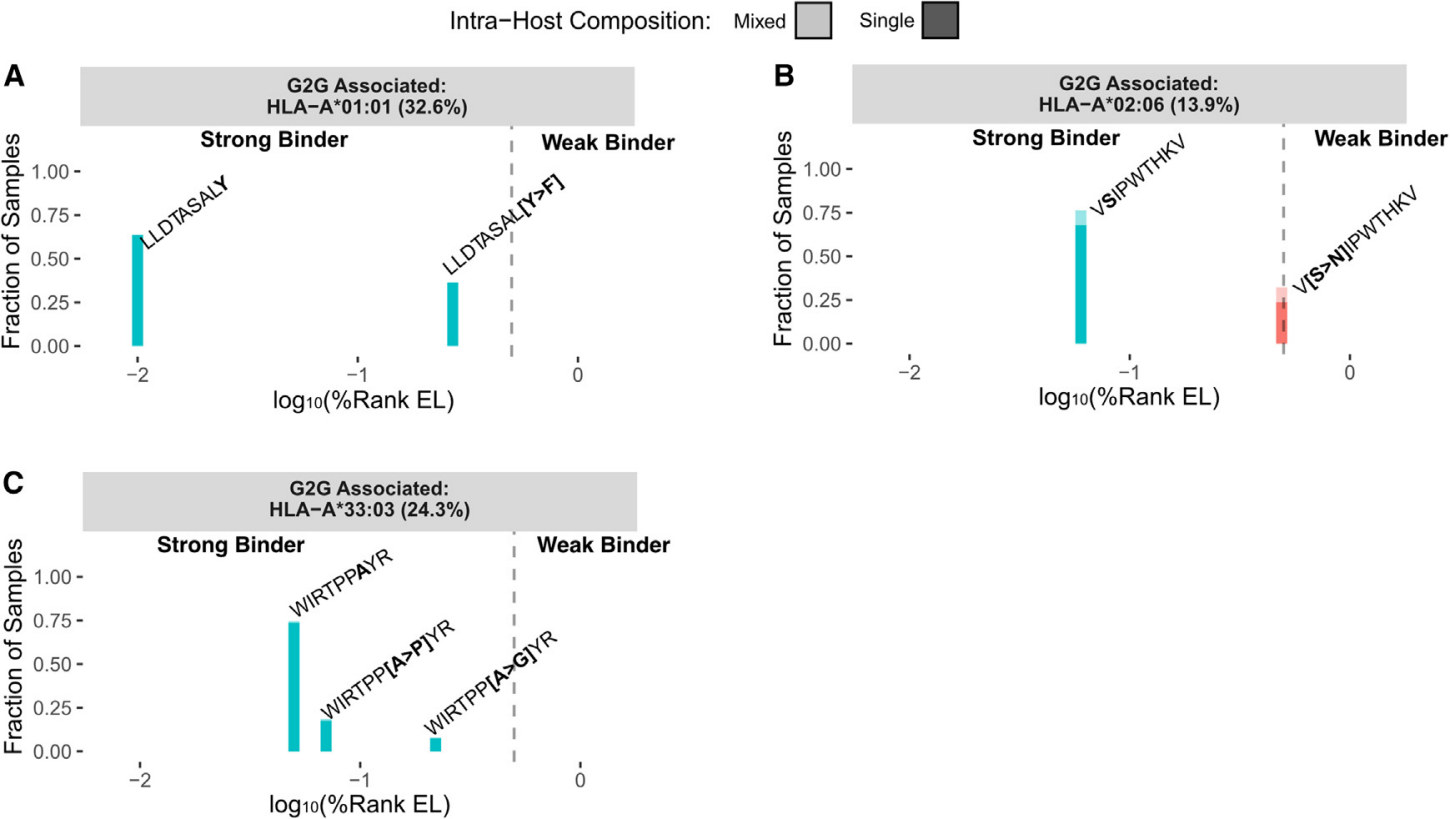
Impact of HBV amino-acid mutations on binding affinity, focusing on viral positions that are associated with HLA-A alleles Each panel refers to the binding affinities of peptides to the associated HLA-A allele, with the frequency of the HLA allele within the cohort indicated in parentheses. Peptide amino-acid sequence is shown above each bar and the relative position of the HLA-associated HBV position is indicated in bold. x axis represents the binding affinity of each peptide, and y axis represents the fraction of carriers of the specified HLA-A allele for which the peptide is present in the viral sample. Shading represents whether the peptide is present as part of a mixture in the intra-host level (multiple peptides in the intra-host viral population) or not. The dotted lines indicate the threshold of a strong binder, defined as elution percentile rank less than 0.5%. (A) Referring to the negative association between HLA-A^∗^01:01 and position 67 (associated amino acid: Y) of HBV precore/core protein in the European cohort. (B) Referring to the positive association between HLA-A^∗^02:06 and position 49 (associated amino acid: N) of HBV polymerase protein in the East Asian cohort. (C) Referring to the negative association between HLA-A^∗^33:03 and position 160 (associated amino acid: A) of HBV precore/core protein in the East Asian cohort.

Next, we evaluated the impact of each HBV mutation on the predicted binding affinities to HLA alleles. Figure 5 shows, for each HLA-associated HBV amino-acid position, the impact of each residue (annotated in bold within each epitope sequence) on predicted binding affinities and their respective frequency in carriers of the corresponding HLA-A allele. For all three HLA alleles, we observed that mutations that result in weaker binding affinities are observed at higher frequencies in carriers of the cognate HLA allele compared to other alleles. Conversely, the population reference residue tends to be conserved if the impact of the mutations on binding affinity is minimal. For example, Figure 5A shows that the substitution of tyrosine at position 67 of precore/core with phenylalanine (LLDTASAL**[Y > F]** in the predicted epitope for the associated allele HLA-A^∗^01:01) results in lower binding affinity. Phenylalanine is observed at higher frequencies in carriers of HLA-A^∗^01:01 compared to carriers of other prevalent HLA-A alleles (Figure S17A). For the other prevalent HLA-A alleles, no strong binding epitope overlaps with the position, and hence, the population consensus residue, tyrosine, is largely conserved. A similar observation was made for the two other HLA alleles and the corresponding HBV epitope mutations (Figures 5B, 5C, S17B, and S17C).

## Discussion

By systematically testing for associations between human and HBV genetic variations, we have identified signatures on the HBV genome that reflect selective pressure driven by human genetic variants. We identified associations between an NTCP variant (rs2296651; NTCP S267F) and HBV preS1 mutations, which might reflect escape from the inhibitory effect on viral entry induced by NTCP S267F. We also identified significant associations between HLA-A variation and HBV mutations that overlap with epitopes, pinpointing escape mutations that allow viral evasion from HLA-A presentation and CTL recognition.

A myristoylated lipopeptide that mimics the preS1 receptor-binding region (Bulevirtide, also known as Myrcludex B) can bind to NTCP to block viral entry.^69^^,^^70^^,^^71^ Using such preS1-derived peptides, a previous study was able to fine-map preS1 residues that are essential for binding to NTCP. The study revealed that two sets of preS1 residues 16–20 (27–31 in our genotype C-based sequence) and 34–48 (45–59 in our genotype C-based sequence) impacted binding, while residues in between them did not.^72^ This is consistent with the Alpha-Fold model we constructed (Figure S2), where the first set of preS1 residues is located within the NTCP binding tunnel and the second set of residues is in proximity to patch 1 of NTCP, with residues in between forming a “loop” that is not in contact with NTCP.

Regarding the human NTCP variant (rs2296651, NTCP S267F), *in vitro* studies have demonstrated that the variant has an inhibitory effect on viral entry. Cell line-based studies suggest that preS1-derived peptides cannot bind to NTCP S267F homozygous clones,^68^^,^^73^ and consequently, HBV infection could not be supported.^68^^,^^74^ In NTCP S267F/WT heterozygous clones, HBV infection could be supported,^74^ albeit potentially less efficiently.^68^ Genetic studies in Asian populations support such a protective effect, where heterozygous carriers of the variant have decreased susceptibility to HBV,^12^^,^^13^^,^^14^^,^^15^^,^^16^^,^^17^^,^^18^^,^^19^ a higher likelihood of spontaneous clearance,^11^ and a decreased risk of developing cirrhosis^13^^,^^15^^,^^19^ or HCC^12^^,^^14^^,^^17^ in individuals with chronic hepatitis B. Homozygous carriers of the variant could still be infected with HBV,^15^^,^^19^ albeit rarely. Limited data suggest that they’re asymptomatic if infected.^13^ Interestingly, viral escape mutations might have enabled infection of homozygous carriers. A study has pinpointed viral escape mutations in an individual with chronic hepatitis B who is a homozygous carrier of NTCP S267F and infected with HBV genotype B.^75^ The identified mutations include the preS1 S17A mutation, which we have found in this study to be associated with NTCP S267F for both genotype C and B.

The positive association that we identified between the NTCP S267F variant and three preS1 residues (17A, 35R, 51P), along with evidence of intra-host selection, suggests that the escape mutations may confer a fitness advantage for HBV within carriers of NTCP S267F. However, results from our peptide binding assays suggest that, compared to preS1 peptides derived from WT genotype C sequences (Myr-WT), peptides that carry the mutations (Myr-ARP) only displayed a partial increase in binding affinity to NTCP S267F. A reason that only a small difference in binding affinity was detected could be that the preS1 mutations might also play a role in downstream processes that are essential for preS1-NTCP internalization. For example, it has been shown that the presence of preS1 is required for NTCP oligomerization and that NTCP oligomerization is essential for preS1-NTCP internalization.^76^^,^^77^ Furthermore, the NTCP S267F variant has been shown to reduce NTCP oligomerization.^78^ Thus, an explanation could be that the preS1 escape mutations also improve the oligomerization efficiency of NTCP S267F-WT heterodimers or NTCP S267F homodimers. Further studies are needed to uncover the exact mechanism.

Although our study supports the protective role of NTCP S267F in individuals with chronic hepatitis B, reflected by significantly reduced serum ALT levels, we only observed a weak effect of HBV escape haplotypes on normalizing serum ALT levels. A likely explanation is that the biomarkers do not directly reflect the efficiency of viral entry and are influenced by multiple mechanisms, which may obscure the direct effects. Additionally, due to the overlapping reading frames of HBV, preS1 mutations associated with NTCP S267F also encode non-synonymous substitutions on the polymerase protein. It is thus possible that the impact on polymerase function may have counterbalanced any functional differences resulting from more efficient viral entry. Finally, the NTCP S267F variant is also associated with decreased bile acid (taurocholate) uptake by hepatocytes.^79^ This has been shown to result in increased serum bile acids levels, based on case reports of homozygous NTCP S267F carriers who are otherwise relatively healthy^80^^,^^81^ and on a GWAS.^82^ Because over-accumulation of bile acids in hepatocytes is thought to lead to liver injury,^83^ the variant may indirectly affect the clinical course of hepatitis B through its effect on liver inflammation.

Our findings also have implications with regards to treatment strategies that should be applied to NTCP S267F carriers. Given that entry inhibitors of NTCP such as Bulevirtide have been designed based on WT HBV sequences, their efficacy in NTCP S267F carriers are expected to be lower. As such, further clinical trials could investigate the implications and most optimal dosing regimens in NTCP S267F carriers.

The associations between HBV mutations and HLA-A variation suggest viral escape from human CTL recognition. This is supported by our observation that mutations within epitopes that lead to lower predicted binding affinity are enriched in carriers of their cognate HLA-A alleles. As such, our findings provide direct genetic evidence of HBV escape from HLA recognition, previously shown in other viruses such as HIV-1^34^^,^^84^^,^^85^^,^^86^ and HCV.^33^^,^^87^^,^^88^^,^^89^^,^^90^

A limitation of our study is that intra-host and inter-host selection processes, which occur during the infection and transmission process, respectively, were not explicitly differentiated. For example, G2G associations could be driven by differences in susceptibility to infection or development of chronicity that is dependent on both host and pathogen genetic factors. Alternatively, G2G associations could be the result of convergent intra-host selection of mutants, illustrating the emergence of escape mutations and adaptation of viruses to the host genetic background during the course of infection. Owning to the relatively high mutation rate of HBV compared to other DNA viruses,^26^ intra-host selection has been demonstrated to be a valid mechanism during chronic HBV infection.^91^ Past studies in fast-evolving viruses such as HIV^21^^,^^84^ lead us to believe that HLA-induced viral escape mutations that we observed in this study might be primarily driven by intra-host selective forces. On the other hand, the characteristics of the selective mechanism responsible for associations between NTCP S267F and HBV preS1 mutations are less clear. Our observation of increased intra-host positive selection within the preS1 receptor-binding region in rs2296651-G/A individuals and examples of valid evolutionary trajectories toward escape haplotypes support the presence of intra-host selective pressure. However, given that information regarding length of infection was not available as part of this study, further longitudinal studies with paired HBV and human genomic data would be required to validate our interpretations and disentangle these two levels of selective forces more directly.

Another limitation of our study is that the overall sample size is modest and primarily composed of individuals with East Asian ancestry. Our findings regarding NTCP were specific to the East Asian cohort. One explanation could be that the burden of HBV infection in East Asia has imposed stronger positive selection pressure on humans compared to Europe, hence increasing the frequency of protective NTCP variants. Another explanation could be the limited statistical power within the European cohort due to the small sample size. Polymorphisms in *NTCP* that impact function have also been identified in European and African populations.^79^ Thus, further studies with paired human and HBV genomic data in other populations, such as African populations where the burden of HBV infection is also among the highest, would strengthen the findings reported here.

A final limitation of our study is that human exome rather than whole-genome sequencing data were utilized. This means that there might be potential associations within non-exonic regions of the genome that this study missed. For example, it remains unclear whether genetic variants within the regulatory regions of *SLC10A1* could also induce evolutionary changes in HBV.

Overall, our results are consistent with previous studies that have established human NTCP and HLA genetic variations as determinants of hepatitis-related outcomes but provide additional genetic evidence that they could also drive HBV evolutionary changes. We also illustrate the potential of the G2G approach as an agnostic and hypothesis-free method to identify mechanisms involved in host-pathogen interactions, revealing insights that can be relevant for treatment and vaccine development.

## Data and code availability

Summary statistics are available on Zenodo (https://doi.org/10.5281/zenodo.8279455). Software code is available on GitHub (https://github.com/zmx21/G2G-HBV-Snakemake). Viral sequencing data have been deposited on the European Genome-Phenome Archive (ega-archive.org) under accession EGA: EGAS00001003689. Human exome sequencing data are not available due to privacy concerns.

## Supplemental information

Document S1. Figures S1–S17, Tables S1–S8, and Methods S1 and S2

Document S2. Article plus supplemental information
