# Supplementary material for: Joint host-pathogen genomic analysis identifies hepatitis B virus mutations associated with human NTCP and HLA class I variation
Source: Am J Hum Genet. 2024 May 14;111(6):1018–34. doi: 10.1016/j.ajhg.2024.04.013 (PMC11179264; doi:10.1016/j.ajhg.2024.04.013)
Supplement: Document S1. Figures S1–S17, Tables S1–S8, and Methods S1 and S2 [file mmc1.pdf]

The American Journal of Human Genetics, Volume *111*

Supplemental information

Joint host-pathogen genomic analysis identifies hepatitis B virus mutations associated with human *NTCP* and HLA class I variation

Zhi Ming Xu, Gnimah Eva Gnouamozi, Sina Rüeger, Patrick R. Shea, Maria Buti, Henry LY. Chan, Patrick Marcellin, Dylan Lawless, Olivier Naret, Matthias Zeller, Arne Schneuing, Andreas Scheck, Thomas Junier, Darius Moradpour, Ondrej Podlaha, Vithika Suri, Anuj Gaggar, Mani Subramanian, Bruno Correia, David Gfeller, Stephan Urban, and Jacques Fellay

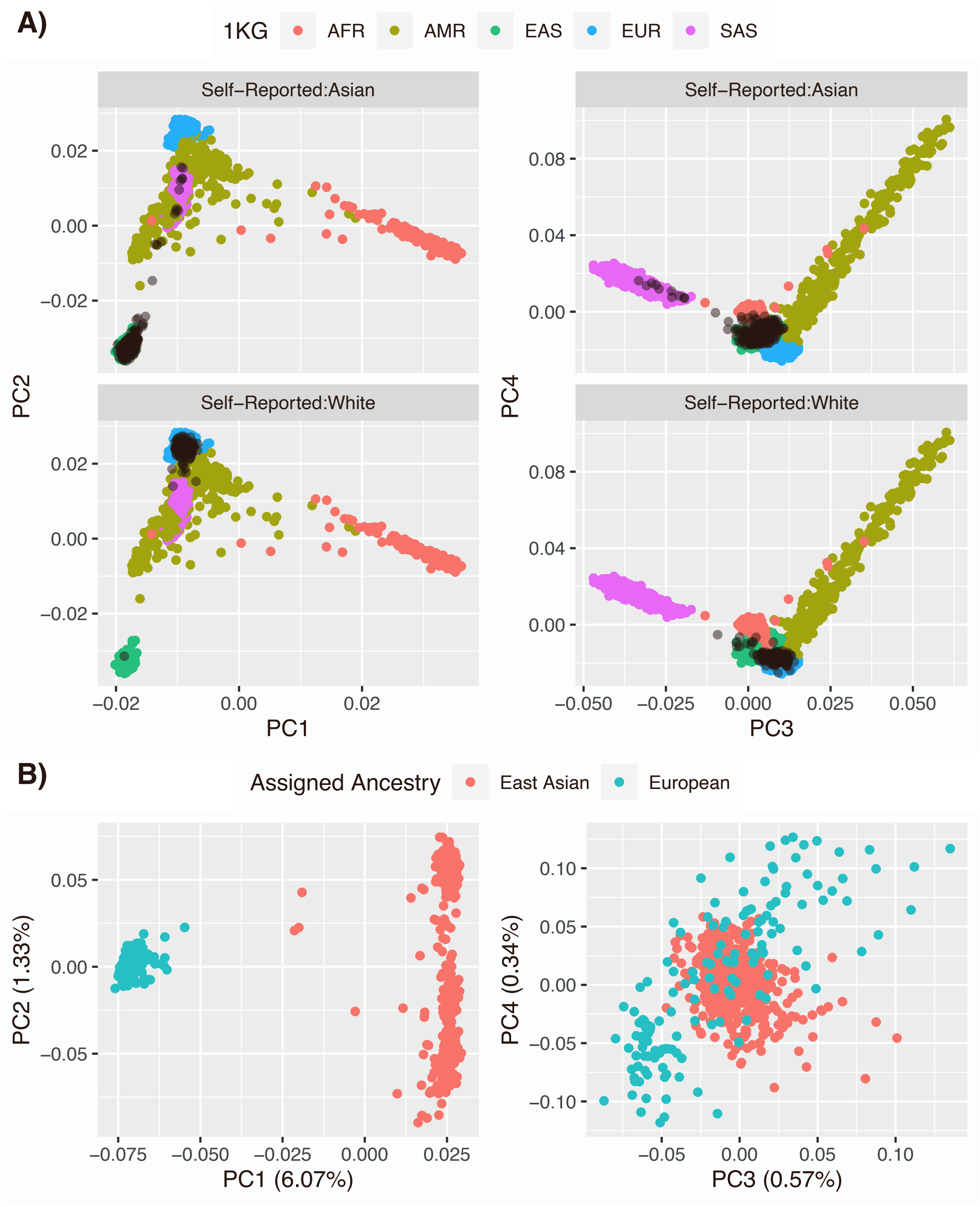

Figure S1: PCA representing human ancestry. a) Principal components based on 1000 Genomes (1KG) samples. Study participants were overlaid in the same principal component space using loadings derived from 1KG. Coloured dots represent 1KG populations and black dots represent study participants. Facet grids represent the self-reported race of study participants. **b)** Principal components re-calculated based on only retained study participants within the East Asian and European ancestry groups. Colors indicate assigned ancestry based on hierarchical clustering applied to 1KG based principal components.

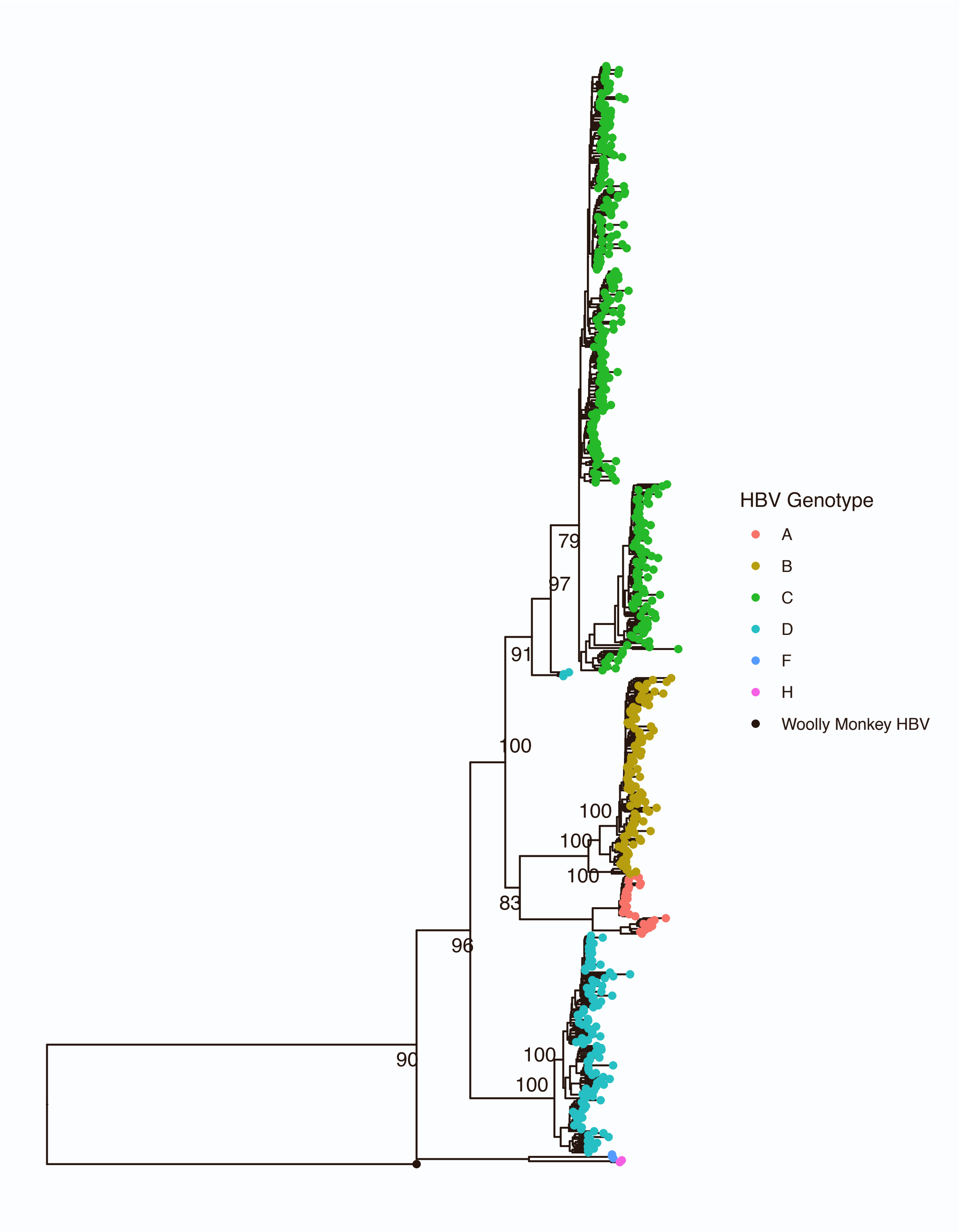

Figure S2: Phylogenetic tree of HBV sequences. Phylogenetic tree constructed using a maximum likelihood-based algorithm (IQ-TREE) from multiple sequence alignment of HBV sequences with Woolly Monkey HBV as the outgroup. Colours indicate HBV genotype. Bootstrap support values were calculated using UFBoot with 1000 replicates.

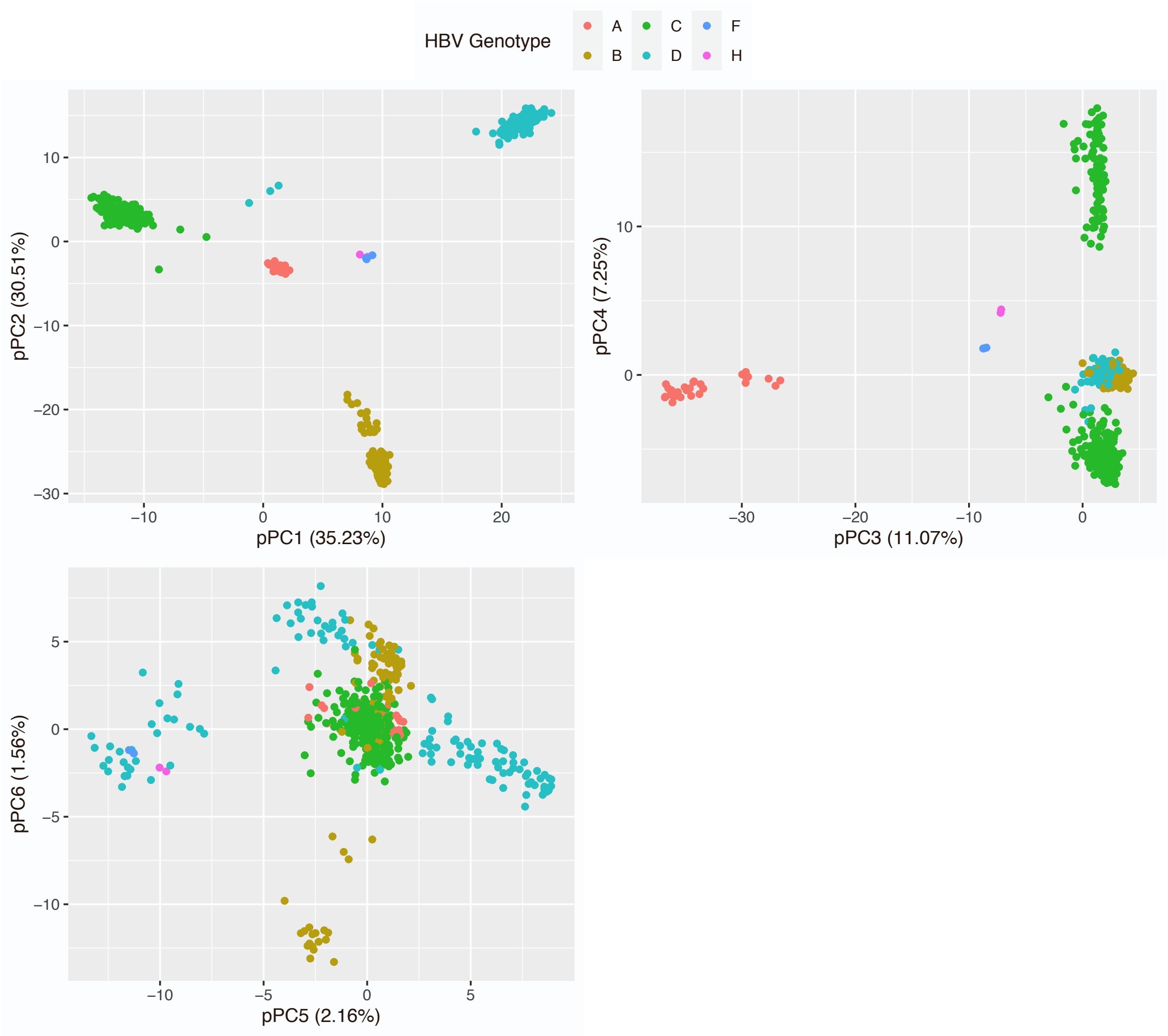

Figure S3: Phylogenetic principal component. Phylogenetic principal components (pPCs) derived from HBV amino acid variants and maximum-likelihood phylogenetic tree. Colors represent HBV genotypes and the percent of variance explained are indicated in brackets for each pPC.

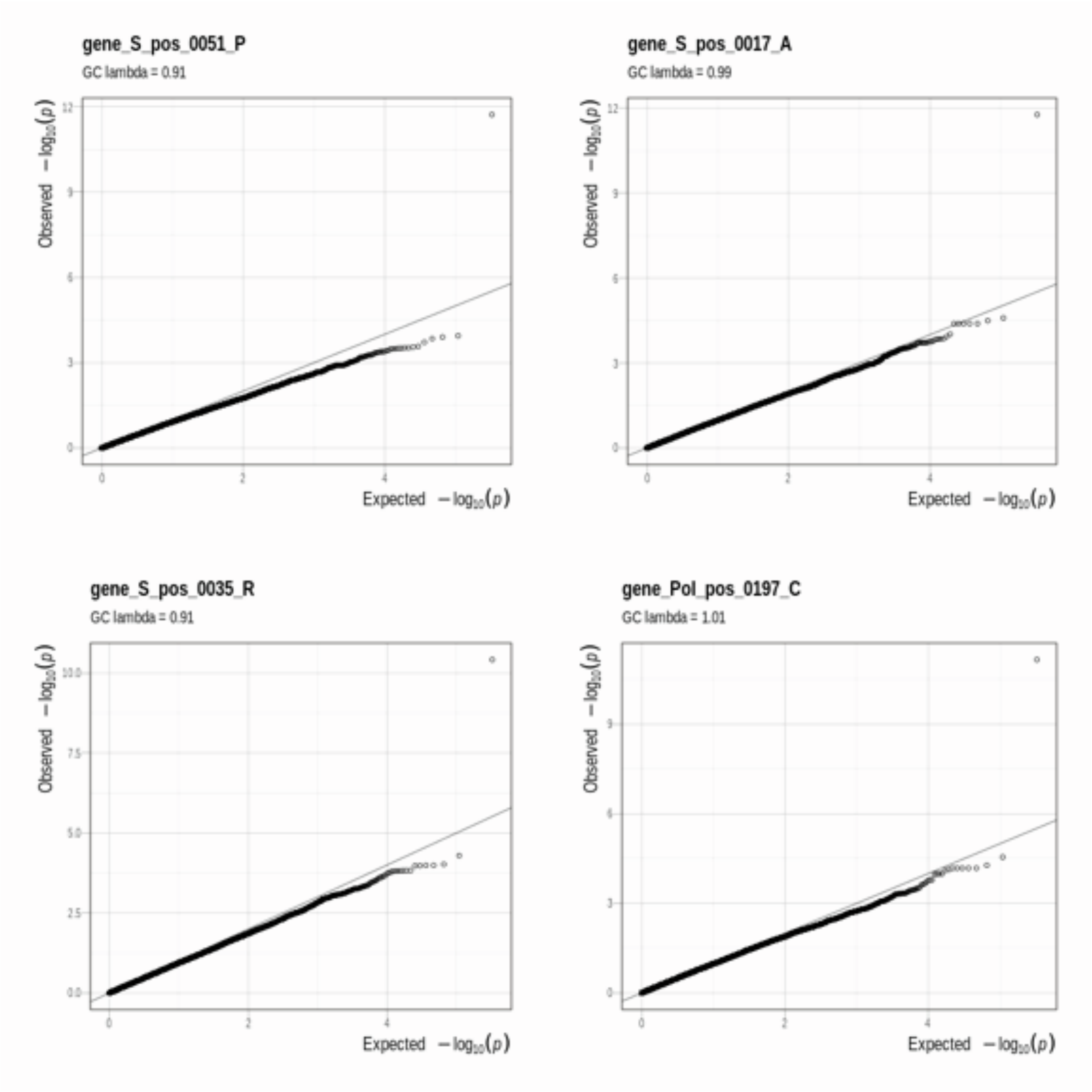

Figure S4: QQ plots for GWASs conducted on HBV variants which are associated with rs2296651. Observed p-values against expected p-values under the null chi-squared distribution.

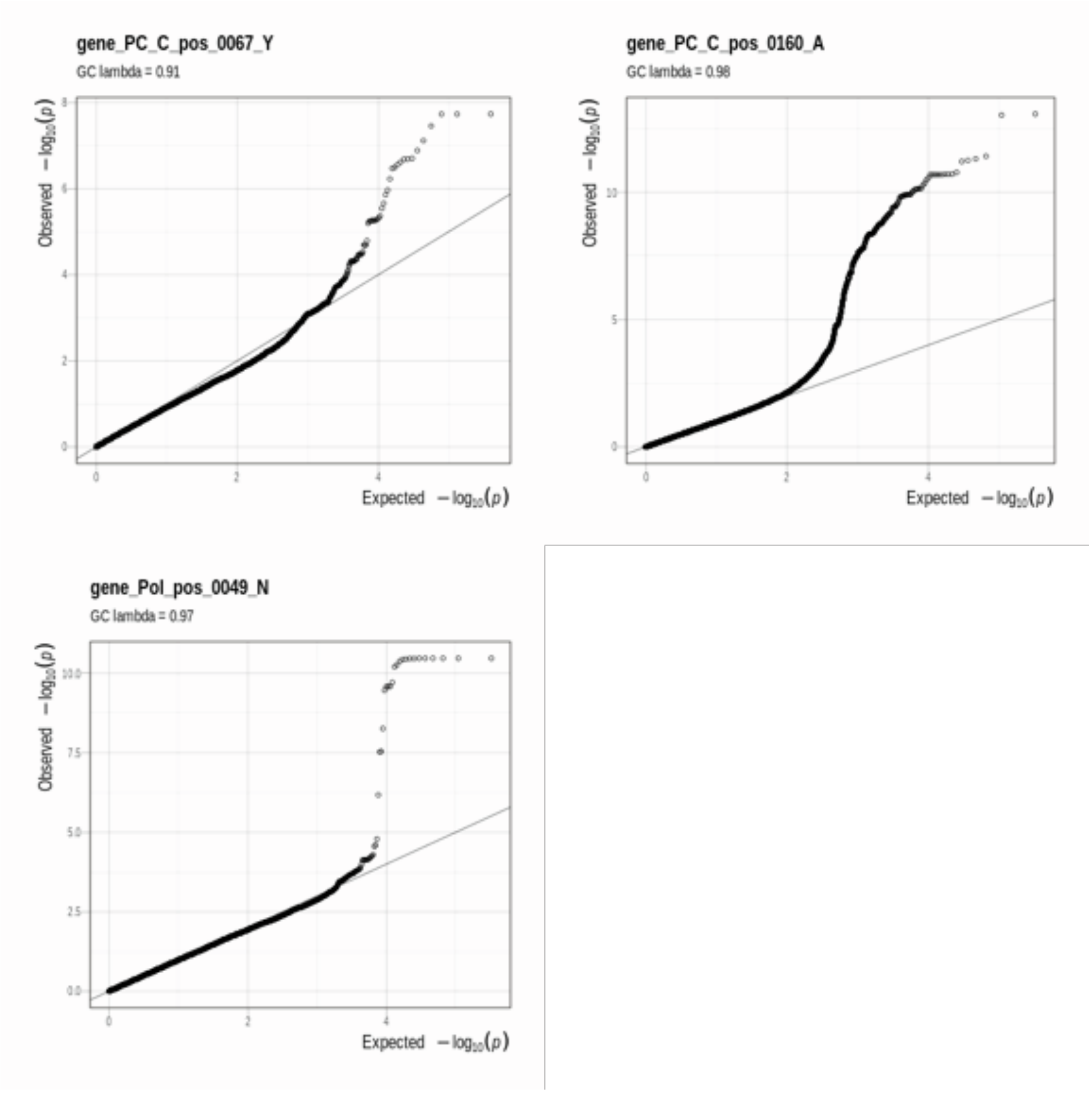

Figure S5: QQ plots for GWASs conducted on HBV variants that are associated with HLA Class I variants. Observed p-values against expected p-values under the null chi-squared distribution.

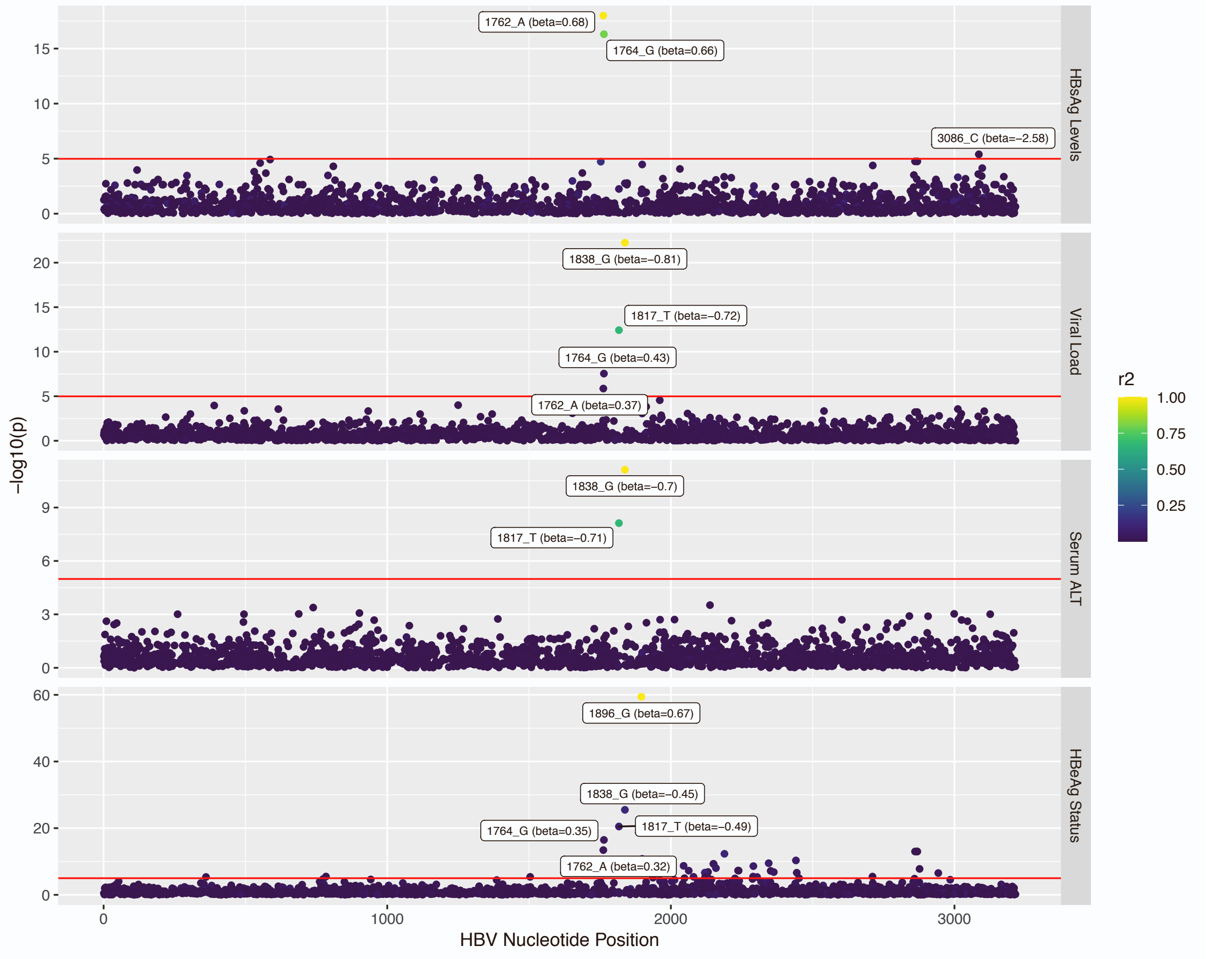

Figure S6: HBV nucleotide variants associated with markers of HBV infection. Variants were considered present if detected above 15% within the intra-host viral population. Quantitative markers of HBV infection were inverse-normal transformed and regressed against nucleotide variants using linear regression adjusting for age, sex, HBeAg status, top 4 human principal components, and top 6 phylogenetic principal components. HBeAg was regressed against nucleotide variants using logistic regression adjusting for age, sex, top 4 human principal components, and top 6 phylogenetic principal components. Red line shows Bonferroni corrected significance threshold of 1.03×10^{-5} (4858 tested nucleotide variants). Color represents the strength of linkage disequilibrium with the strongest association.

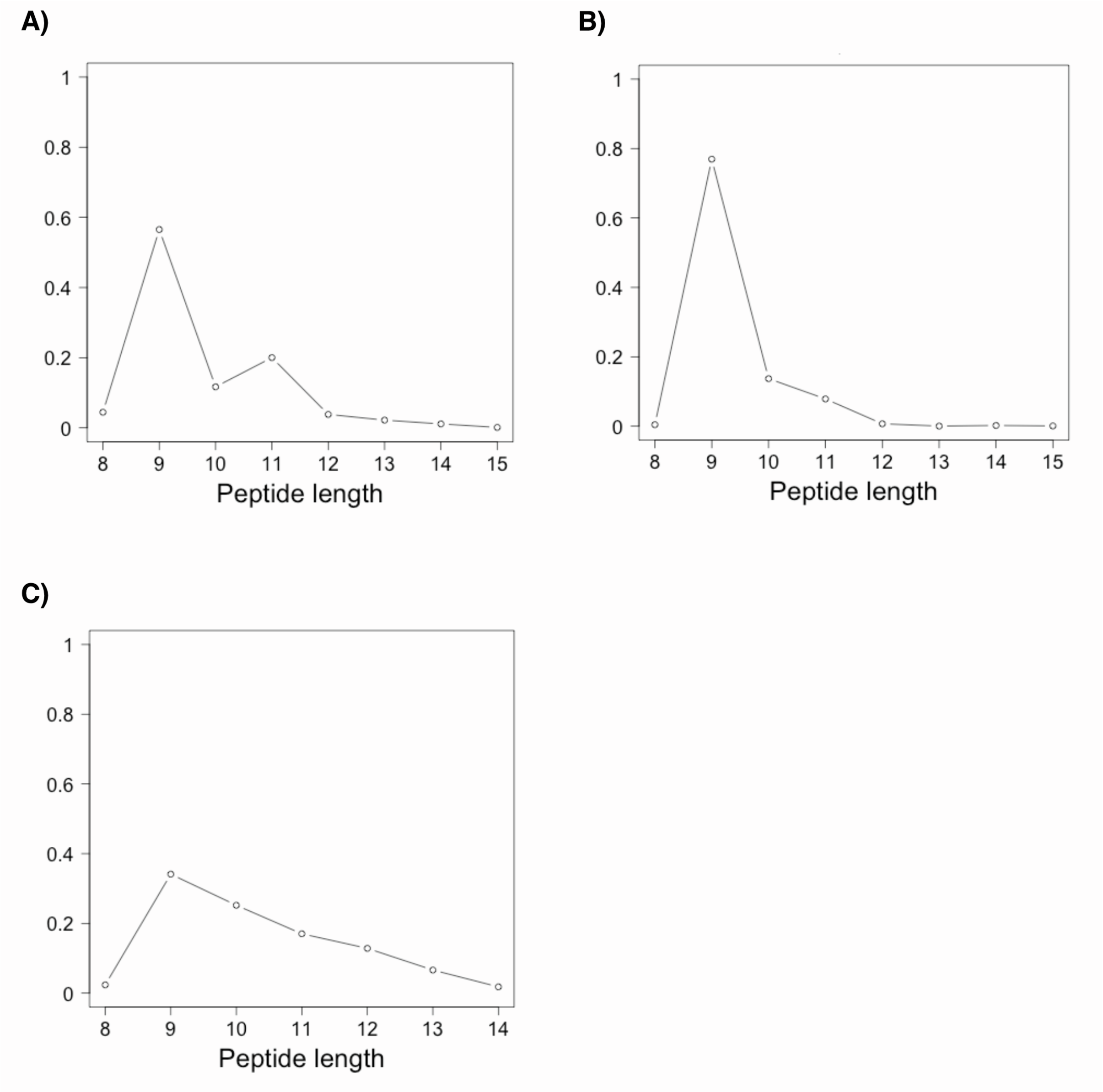

Figure S7: Peptide length distribution for HLA-A alleles. A) HLA-A*33:03 **B)** HLA-A*02:06 **C)** HLA-A*01:01

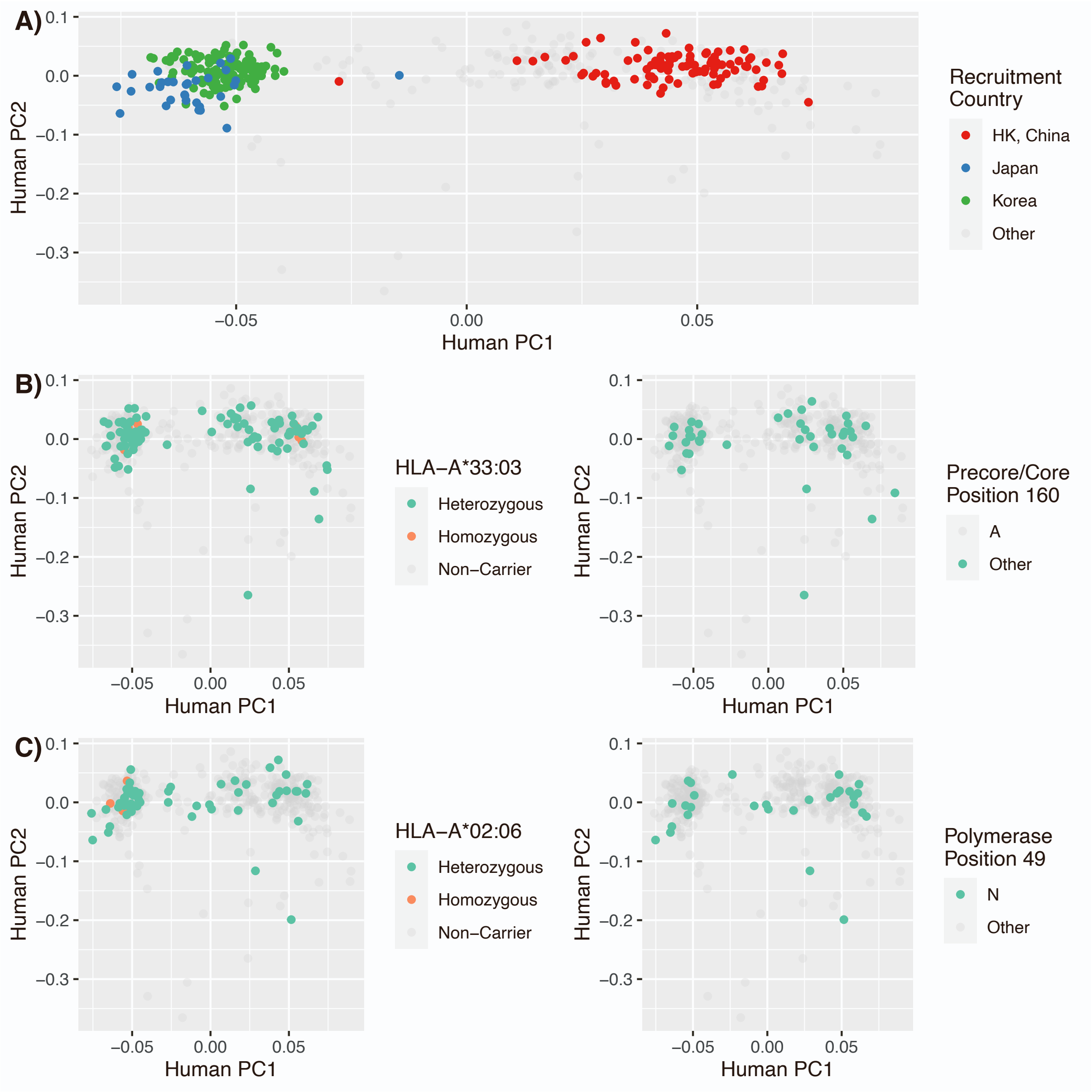

Figure S8: Prevalence of HLA-alleles and associated HBV mutations within the East Asian cohort. A) Human genetic principal components (PCs) capture genetic differences of individuals recruited from different East Asian countries, revealing ancestry subgroups. **B)** Both HLA-A*33:03 and the associated HBV mutation (Precore/Core position 160) are present across multiple recruitment countries and ancestry subgroups. **C)** Both HLA-A*02:06 and the associated HBV mutation (Polymerase position 49) are present across multiple recruitment countries and ancestry subgroups.

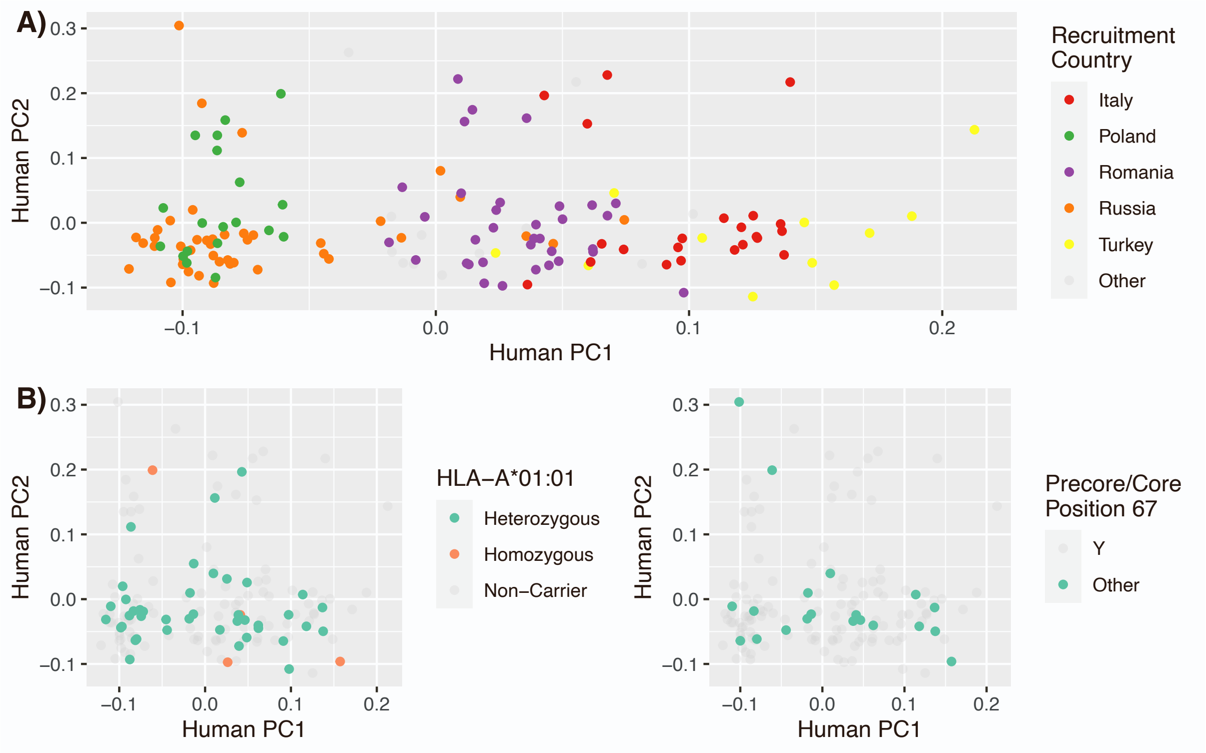

Figure S9: Prevalence of HLA-alleles and associated HBV mutations within the European cohort. A) Human genetic principal components (PCs) capture genetic differences of individuals recruited from different European countries, revealing ancestry subgroups. **B)** Both HLA-A*01:01 and the associated HBV mutation (Precore/Core position 67) are present across multiple recruitment countries and ancestry subgroups.

	Human_PC1	Human_PC2	Human_PC3	Human_PC4	phylo_PC1	phylo_PC2	phylo_PC3	phylo_PC4	phylo_PC5	phylo_PC6
HLA−A*33:03	0.01	0	0	0	0.01	0	0	0	0.01	0.01
HLA−A*02:06		0	0	0	0.01	0.01	0	0.01	0	0
Precore/Core Pos 160 (AA:A)			0	0	0	0	0	0.02	0.02	0.02
Polymerase Pos 49 (AA:N)				0	0	0.01	0	0	0	0

	Human_PC1	Human_PC2	Human_PC3	Human_PC4	phylo_PC1	phylo_PC2	phylo_PC3	phylo_PC4	phylo_PC5	phylo_PC6
HLA−A*01:01	0	0.01	0.01	0.01	0	0	0	0.01	0	0.01
Precore/Core Pos 67 (AA:Y)		0	0.01	0	0.02	0	0	0.02	0	0.05

Figure S10: Correlation between HLA-A alleles or association HBV mutations and human or phylogenetic principal components. Top panel refers to associations identified in the East Asian cohort, and bottom panel associations identified in the European cohort. Values indicate coefficient of determination (r^2) calculated using Point-Biserial correlation.

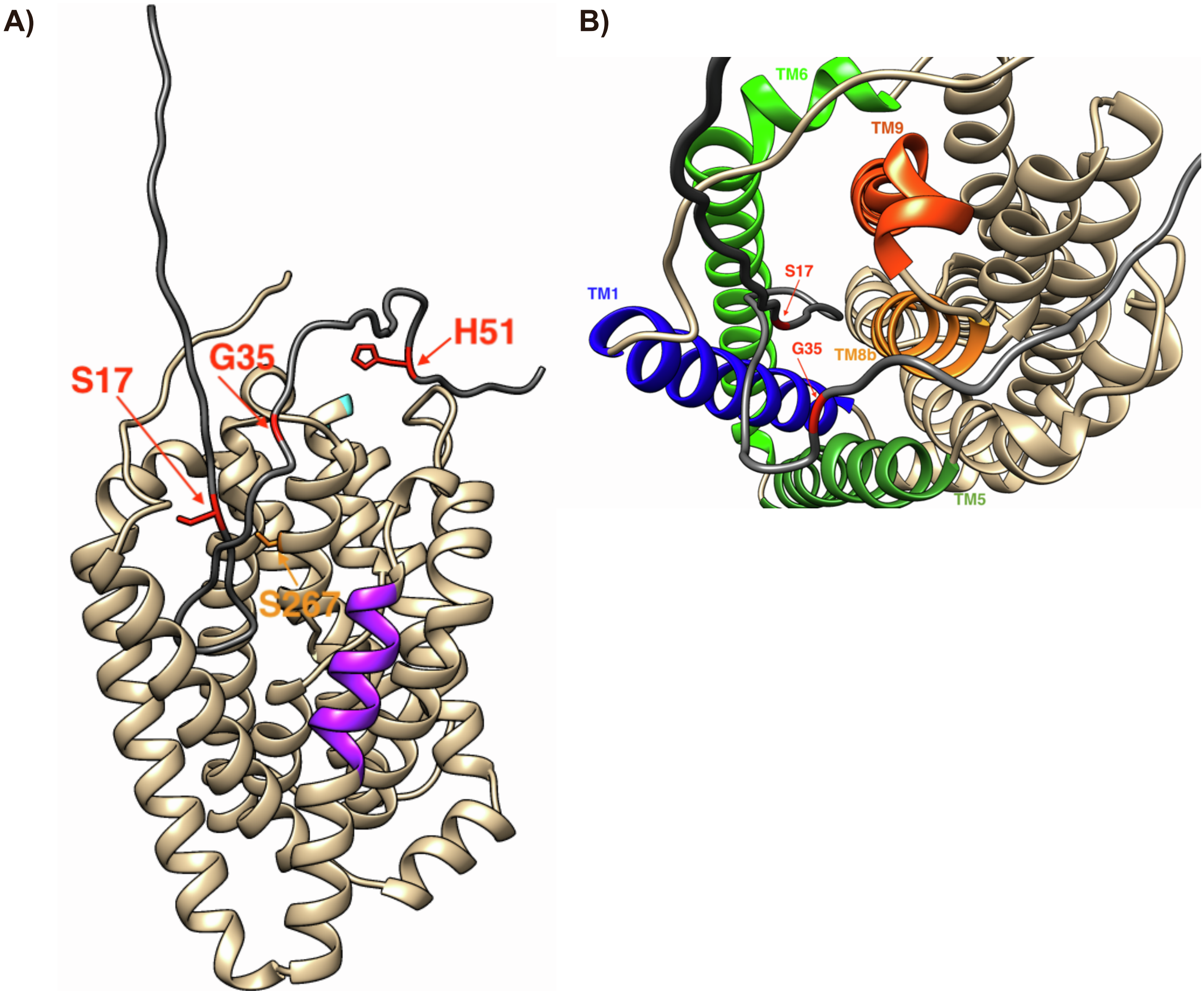

Figure S11: Top ranking model of preS1-NTCP binding based on AlphaFold-Multimer. The wildtype NTCP sequence (in tan) and a peptide (in grey) derived from HBV preS1 (aa 2-60) based on the Genotype C consensus sequence was used to construct the model. **A)** Position of the human variant (NTCP S267F) is labelled in orange, and positions of associated preS1 residues (position S17, G35, and H51) are labelled in red. Patch 1 (aa 84-87, in cyan) and patch 2 (aa 157-167, in purple) of NTCP are regions outside of the NTCP tunnel that were previously reported to be also important for preS1 binding. **B)** Zoom-in view representing extracellular view of the NTCP heterozygous, with domains color coded according to the published EM-structure (Asami et al, 2022).

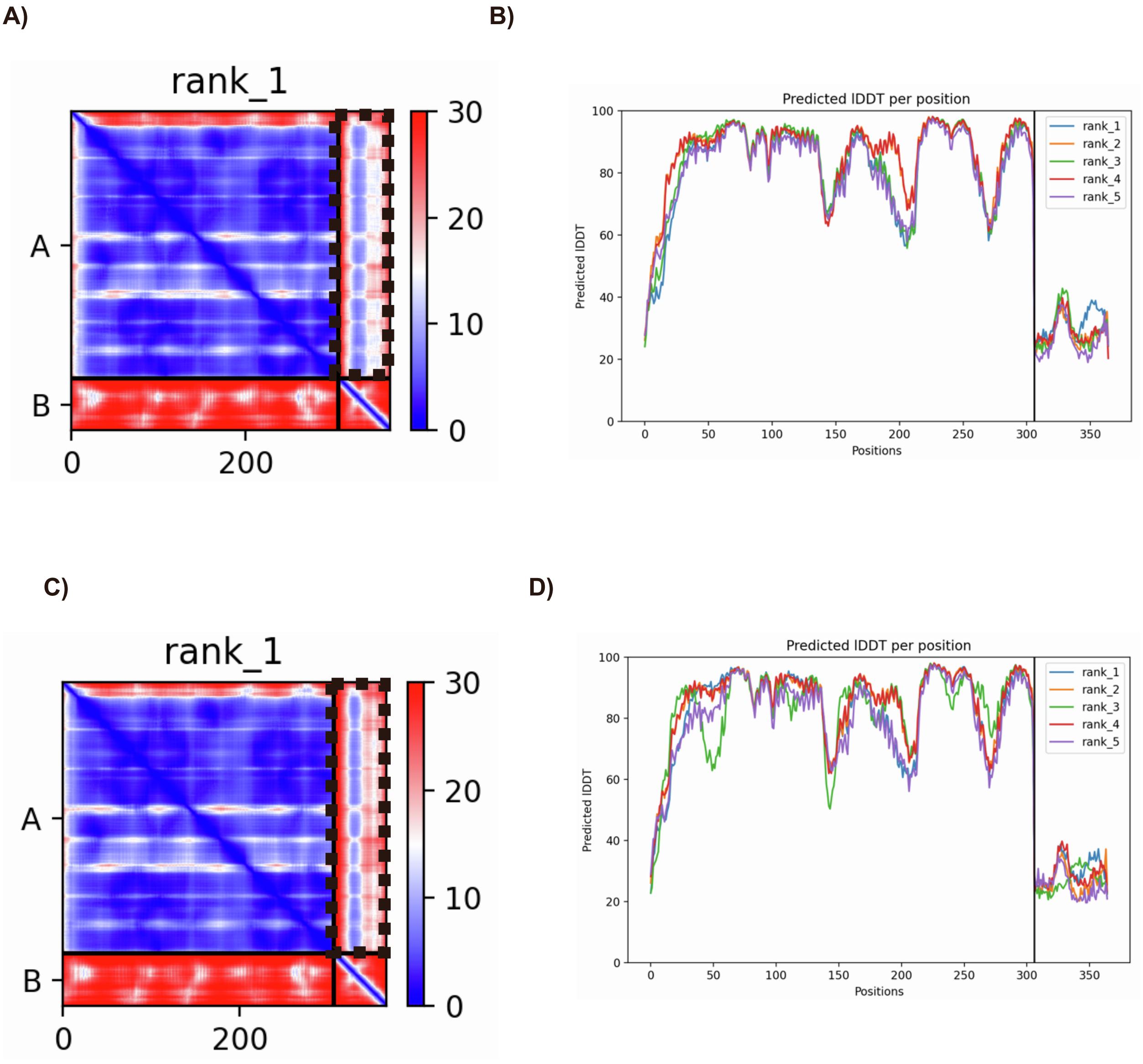

Figure S12: Predicted aligned error (PAE) score matrices and predicted local distance difference test (pLDDT) scores of AlphaFold-Multimer preS1-NTCP models. The PAE score is a per-residue accuracy metric between pairs of residues (x,y). The dotted box refers to residues within the matrix that were used to calculate the average PAE for the preS1-NTCP interface. The pLDDT is a per-residue accuracy metric of the local confidence of the structure. The NTCP residues are left of the vertical line and the HBV preS1 residues are right of the vertical line. **A)** PAE of top-ranking model based on NTCP WT and preS1 WT **B)** pLDDT of models based on NTCP WT and preS1 WT **C)** PAE of top-ranking model based on NTCP S267F and preS1 WT **D)** pLDDT of models based on NTCP S267F and preS1 WT.

A)

B)

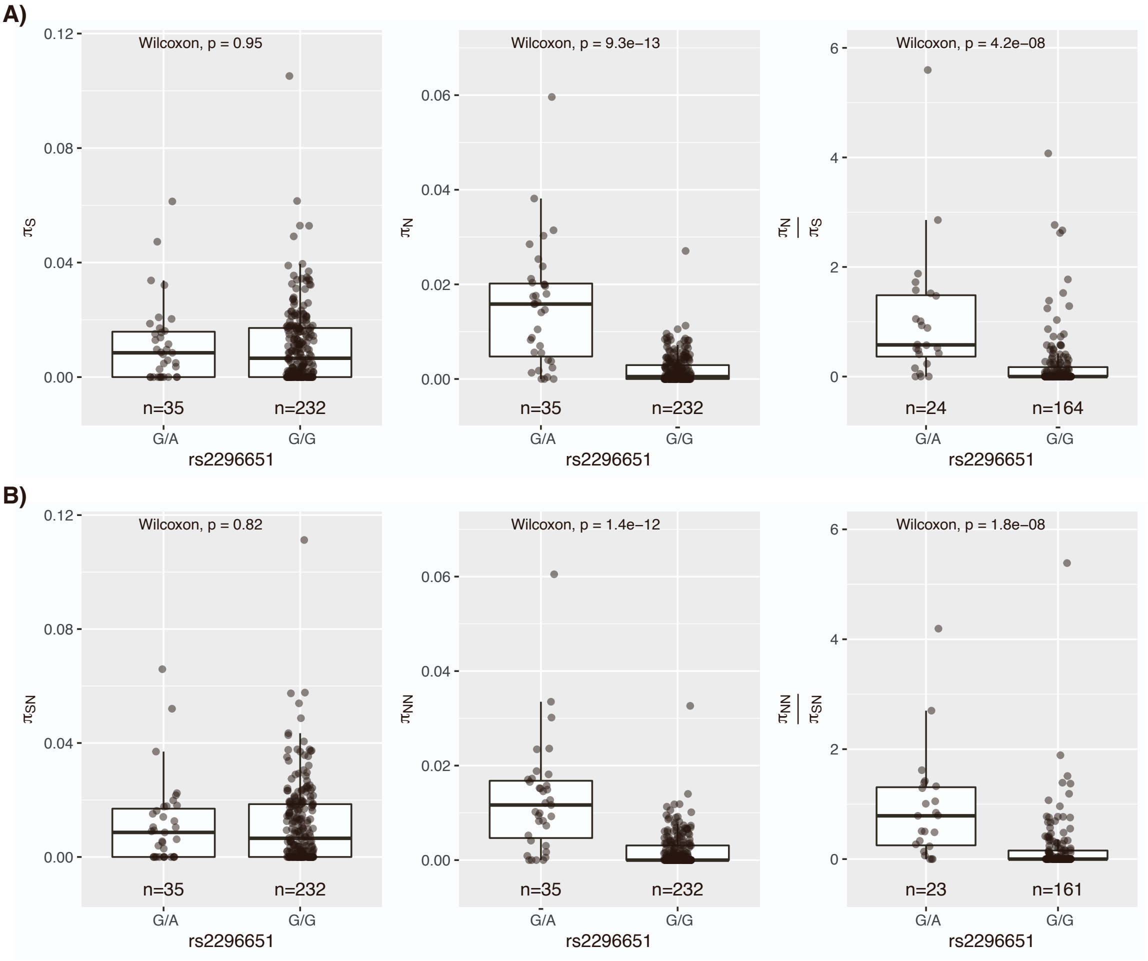

Figure S13: Intra-host non-synonymous and synonymous nucleotide diversity within the preS1 receptor binding region in heterozygous NTCP S267F carriers (rs2296551-G/A) and homozygous NTCP WT carriers (rs2296551-G/G) A) Substitutions and sites are included irrespective of consequence in the polymerase reading frame. π_N represents non-synonymous and π_s represents synonymous nucleotide diversity. **B)** Substitutions and sites are restricted to those that are non-synonymous in the polymerase reading frame.

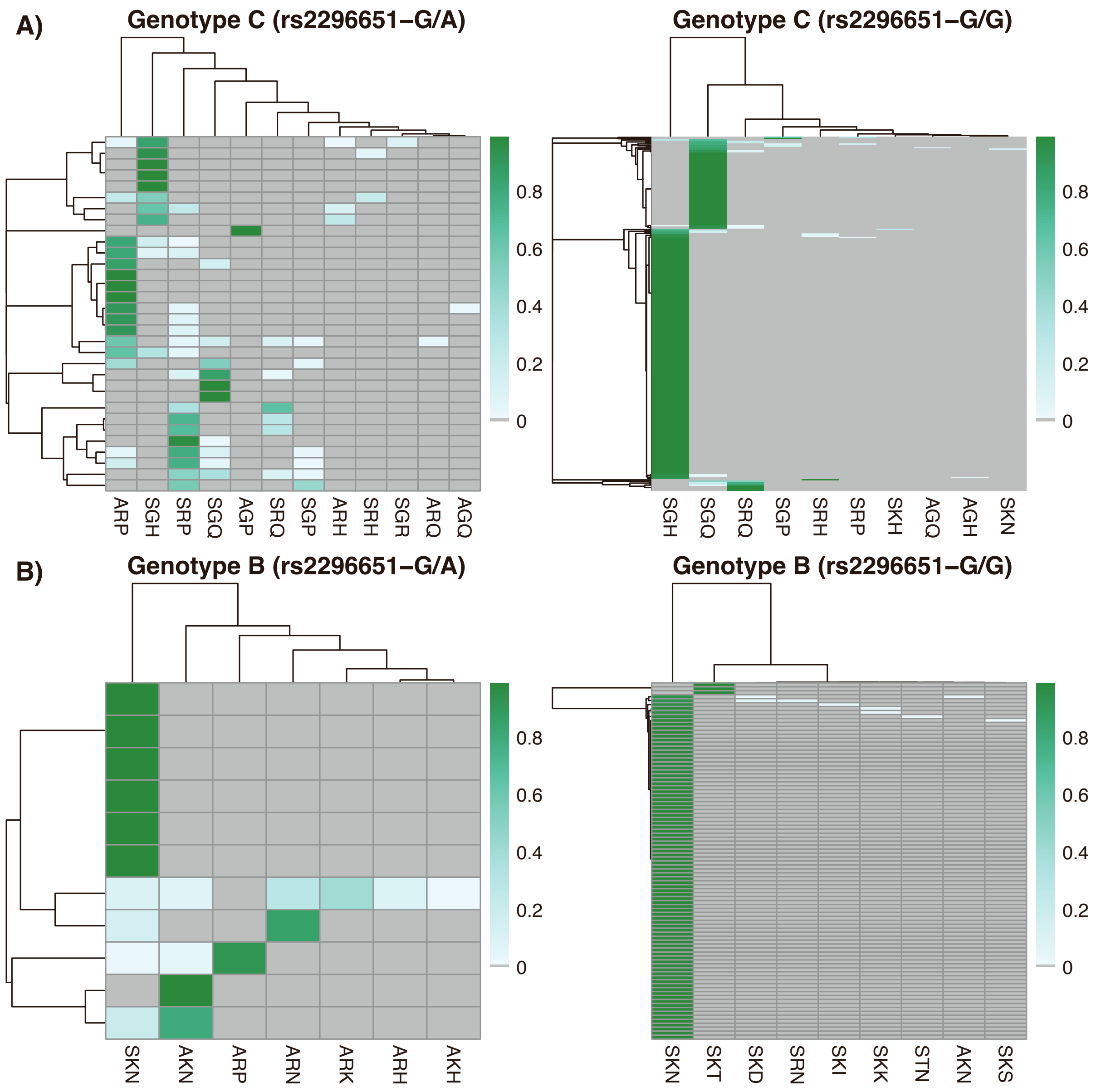

Figure S14: Heatmap of the intra-host composition of HBV preS1 receptor-binding region haplotypes. Haplotypes are grouped according to residues at position 17, 31, and 51 of preS1. Colors indicate the proportion of reads supporting each haplotype within each individual. An entry is grey if the haplotype is absent in the individual.

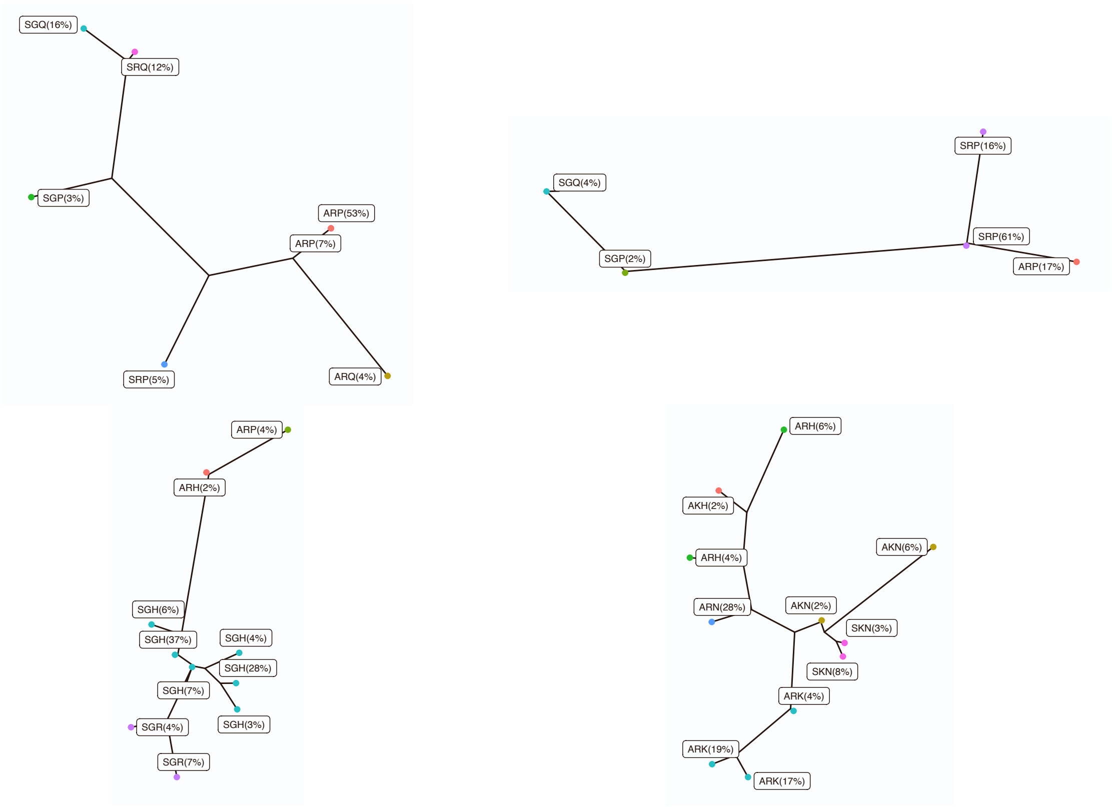

Figure S15: Examples of intra-host evolutionary trajectories. Unrooted tree constructed based on preS1 binding domain nucleotide sequences using a neighbour-joining algorithm. Labels indicate haplotypes based on residues at positions 17,35,51 of preS1. The proportion of intra-host reads supporting each haplotype is indicated in brackets.

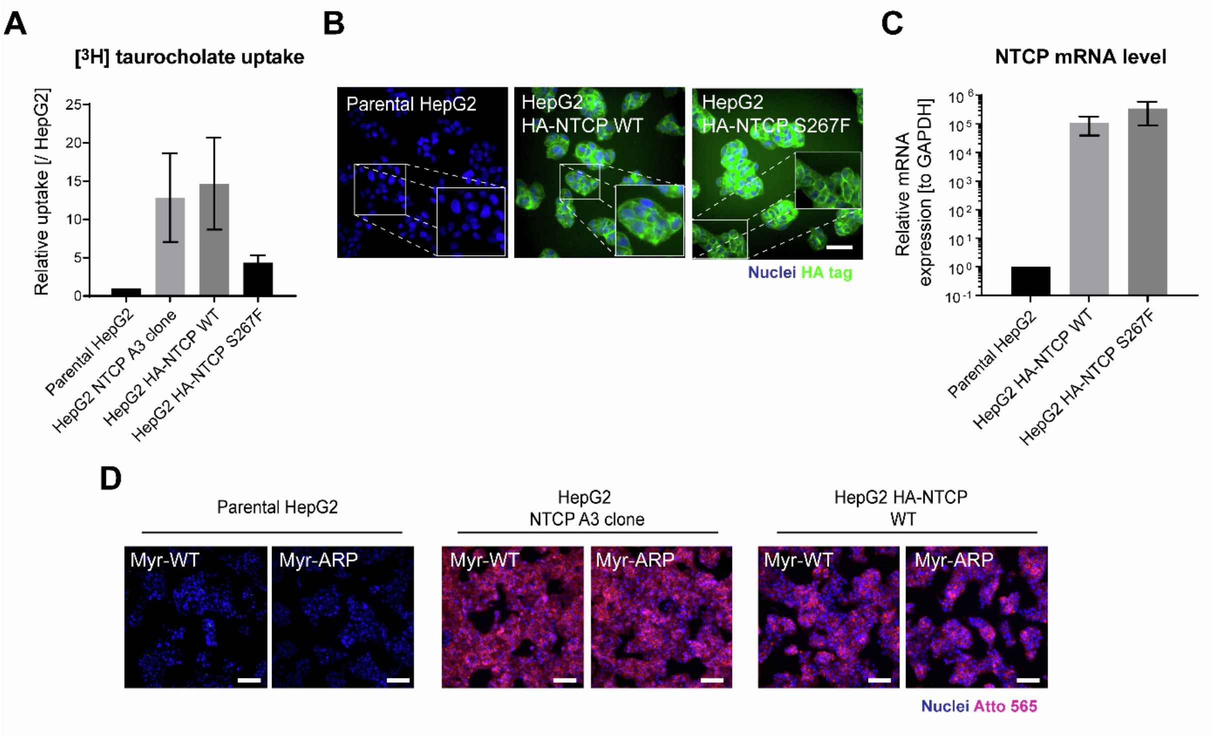

Figure S16: Validation and characterization of NTCP expression in HepG2 HA-NTCP and HA-NTCP S267F stable cell lines and synthesis of myristoylated peptides for affinity binding studies. A) After antibiotic selection, the functionality of NTCP in stable expressing cell lines was evaluated via taurocholate uptake assay comparing parental HepG2 (negative control), HepG2 NTCP A3 clone (positive control), HepG2 NTCP-WT or HepG2 NTCP-S267F cells. **B)** HA- NTCP expressing cell lines, were stained with mAb anti-HA tag and with Alexa 488-conjugated goat anti-mouse IgG, scale bar: 100µm **C**) Intracellular NTCP mRNA expression was measured by RT- qPCR. NTCP expression was calculated relative to housekeeping gene GAPDH and normalized on the expression in parental HepG2 cells. **D**) The binding capacity of the synthetized peptides was evaluated performing binding assay using parental HepG2, HepG2 NTCP A3 clone and HepG2 HA- NTCP cells scale bar: 100µm.

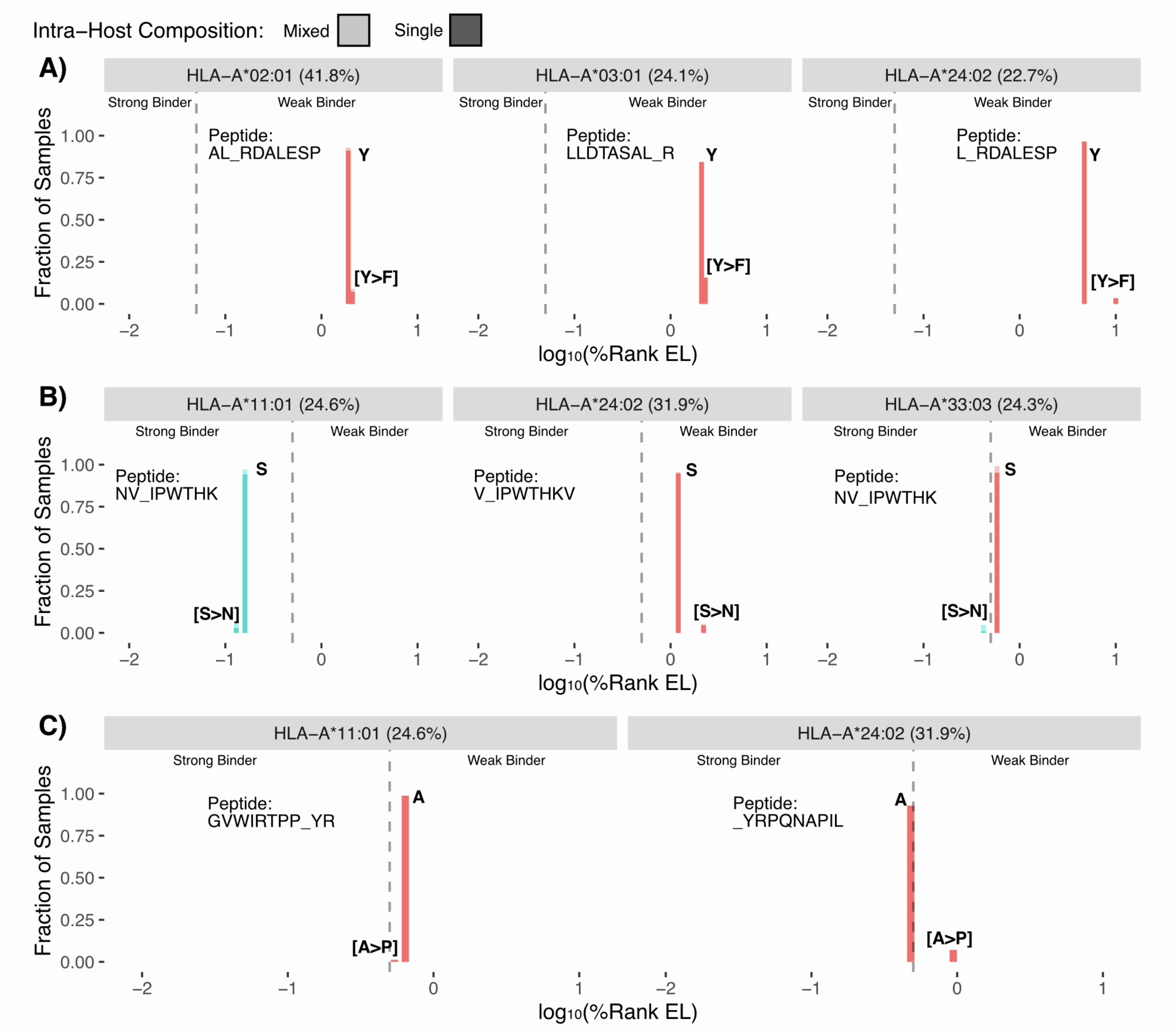

Figure S17: Impact of HBV amino acid mutations on binding affinity to other prevalent HLA-alleles. Each panel refers to the binding affinities of peptides to HLA-A alleles that are prevalent in the cohort (> 20% frequency) but not associated with the HBV position. Peptide amino acid sequence is shown above each bar and the relative position of the HLA-associated HBV position is indicated in bold. X-axis represents the binding affinity of each peptide, and y-axis represents the fraction of samples within carriers of the specific HLA-A allele for which the peptide is present. Shading represents whether the peptide is present as part of a mixture in the intra-host level (multiple peptides in the intra-host viral population) or not. The dotted lines indicate the threshold of a strong binder, defined as elution percentile rank less than 0.5% **(A)** Referring to position 67 of HBV precore/core protein in the European cohort. **(B)** Referring to position 49 of HBV polymerase protein in the East Asian cohort. **(C)** Referring to position 160 of HBV precore/core protein in the East Asian cohort.

Table S1: Summary of demographic characteristics of trial participants included in this study.

Clinical Trial ID	Time of Study	Key Inclusion Criteria	Key Exclusion Criteria
NCT02579382	Nov 2015 – Aug 2017	- Aged 18-65 years<br>- Chronic hepatitis B (HBsAg positivity of >6 months)<br>- HBV DNA ≥2000 IU/ml	- Extensive bridging fibrosis or cirrhosis<br>- Oral antiviral treatment within 3 months of screening<br>- Coinfections with HCV, HIV, or HDV
NCT01940341	Sept 2013 – Oct 2014	- Aged at least 18 years<br>- Chronic hepatitis B<br>- HBeAg negative and HBeAb positive<br>- HBV DNA ≥2000 IU/ml<br>- Serum alanine aminotransferase concentrations of > 60 U/L in men or >38 U/L in women and <10x the upper limit of the normal range.<br>- Treatment-naive or treatment-experienced, provided they meet entry criteria	- Evidence of hepatocellular carcinoma or decompensation.<br>- Treatment with interferon within 6 months<br>- Coinfections with HCV, HIV, or HDV
NCT01940341	Sept 2013 – Dec 2014	- Aged at least 18 years<br>- Chronic hepatitis B<br>- HBeAg positive<br>- Serum alanine aminotransferase concentrations of > 60 U/L in men or >38 U/L in women and <10x the upper limit of the normal range.<br>- Treatment-naive or treatment-experienced, provided they meet entry criteria	- Evidence of hepatocellular carcinoma or decompensation.<br>- Treatment with interferon within 6 months<br>- Coinfections with HCV, HIV, or HDV
NCT02174276	July 2014 – Aug 2016	- Aged at least 18 years<br>- Documented evidence of chronic hepatitis B virus (HBV) infection<br>- HBV DNA ≥2000 IU/ml	- Cirrhosis, inadequate liver function, or hepatocellular carcinoma.<br>- Therapy with immunomodulators, biologics, or investigational agents within 3 months of screening.<br>- Coinfections with HCV, HIV, or HDV

Table S2: Association between HLA-A alleles and HBV amino acid variants in the East Asian cohort. Top association annotated in bold.

		Precore/core: Position 160 (Associated AA: A)			Polymerase: Position 49 (Associated AA: N)		
**HLA Allele**	**Frequency**	**P**	**OR**	**SE**	**P**	**OR**	**SE**
HLA-A*24:02	0.3190	1.85e-01	1.6300	0.367	5.17e-01	0.784	0.376
HLA-A*11:01	0.2460	1.47e-03	4.2200	0.453	6.36e-01	0.800	0.471
HLA-A*33:03	0.2430	**2.10e-10**	**0.0775**	**0.402**	4.63e-01	0.735	0.419
HLA-A*02:01	0.1730	1.17e-01	2.1500	0.487	7.02e-01	0.817	0.529
HLA-A*02:07	0.1470	4.29e-01	1.5200	0.527	1.82e-01	0.475	0.558
HLA-A*02:06	0.1390	1.17e-01	2.1600	0.490	**1.20e-12**	**84.200**	**0.624**
HLA-A*02:03	0.0898	1.96e-01	2.1700	0.598	3.30e-02	0.276	0.604
HLA-A*31:01	0.0827	7.92e-02	0.3290	0.634	4.11e-01	0.533	0.767
HLA-A*11:50	0.0757	3.86e-02	4.7100	0.749	9.52e-02	0.274	0.776
HLA-A*26:01	0.0709	7.69e-01	1.2500	0.755	2.95e-01	0.460	0.740
HLA-A*11:32	0.0449	3.65e-01	2.3800	0.957	5.13e-01	1.880	0.964
HLA-A*11:02	0.0307	5.12e-01	0.4940	1.080	8.39e-01	1.270	1.190
HLA-A*30:01	0.0260	3.91e-01	0.3910	1.100	3.67e-01	0.309	1.300

Table S3: Association between HLA-A alleles and HBV amino acid variants in the European cohort. Top association annotated in bold.

		Precore/core: Position 67 (Associated AA: Y)		
**HLA Allele**	**Frequency**	**P**	**OR**	**SE**
HLA-A*02:01	0.4180	6.13e-02	2.5900	0.510
HLA-A*01:01	0.3260	**9.74e-08**	**0.0676**	**0.505**
HLA-A*03:01	0.2410	7.59e-01	1.1800	0.534
HLA-A*24:02	0.2270	2.18e-01	2.3400	0.691
HLA-A*32:01	0.0709	8.30e-01	1.2800	1.140
HLA-A*26:01	0.0638	2.73e-01	3.9000	1.240
HLA-A*30:01	0.0567	2.28e-01	3.7100	1.090
HLA-A*31:01	0.0567	3.52e-01	3.5900	1.380
HLA-A*68:01	0.0496	2.64e-01	3.8000	1.200
HLA-A*23:01	0.0496	7.24e-01	1.5500	1.230
HLA-A*11:32	0.0426	3.75e-01	3.6500	1.460
HLA-A*11:01	0.0355	2.15e-01	0.0828	2.010
HLA-A*11:50	0.0355	8.62e-01	0.7880	1.370

Table S4: Association between HLA-A amino acid positions and HBV amino acid variants in the East Asian cohort. P-values based on the omnibus test. The conditional p-value represents the association conditioned on the top association (in bold).

HLA-A Position	Possible AA	Precore/core position 160 (Associated AA: A)		Pol position 49 (Associated AA: N)	
		P	P (Conditional)	P	P (Conditional)
10	L,V	1.27e-05	4.00e-01	2.18e-02	4.49e-02
14	L,S	4.66e-12	7.83e-01	2.39e-01	4.94e-01
35	T,S,F,Y	6.20e-12	6.02e-01	**5.76e-05**	NA
82	R,G	1.29e-02	6.73e-01	5.12e-02	7.93e-02
88	R,E,Q,G	1.07e-07	6.24e-01	1.60e-03	3.28e-03
89	N,E	1.27e-08	2.72e-01	2.15e-02	4.20e-02
91	G,R	2.72e-01	6.06e-01	4.80e-01	3.13e-01
92	N,K	8.87e-05	5.81e-01	5.13e-03	1.03e-02
96	Q,H	2.02e-02	8.64e-01	1.17e-02	3.47e-03
99	I,T	**2.90e-13**	NA	1.52e-02	NA
100	H,D	1.93e-03	2.65e-01	1.04e-04	2.28e-04
102	A,E,V	4.43e-01	4.32e-01	1.32e-01	7.97e-02
103	N,D	3.48e-01	3.77e-01	9.19e-02	4.79e-02
105	R,G	1.26e-01	2.39e-01	2.16e-01	1.32e-01
106	I,T	1.26e-01	2.39e-01	2.16e-01	1.32e-01
107	A,L	1.26e-01	2.39e-01	2.16e-01	1.32e-01
108	L,R	1.26e-01	2.39e-01	2.16e-01	1.32e-01
109	R,G	1.26e-01	2.39e-01	2.16e-01	1.32e-01
116	D,A	1.48e-03	4.93e-01	1.69e-01	7.59e-02
121	L,V,I	2.31e-04	4.52e-01	6.85e-04	1.36e-03
123	I,R,M	2.00e-06	9.76e-01	7.50e-04	1.57e-03
125	C,F,Y	1.94e-01	4.81e-01	2.77e-01	1.68e-01
131	P,S	1.22e-03	2.01e-01	1.24e-01	4.91e-02
133	W,G	3.21e-03	3.01e-01	2.28e-04	5.07e-04
140	R,Q,H	3.86e-11	1.12e-01	9.45e-03	1.89e-02
142	D,Y	7.96e-05	1.04e-01	2.65e-03	5.82e-03
153	N,K	6.87e-05	2.87e-01	6.28e-03	1.35e-02
168	T,I	2.51e-03	3.00e-01	2.89e-04	6.41e-04
170	Q,K	2.27e-11	9.42e-02	5.88e-02	9.70e-02
171	H,R	2.51e-03	3.00e-01	2.89e-04	6.41e-04
175	T,A	1.79e-01	2.95e-01	6.22e-02	4.32e-02
177	R,H	1.17e-11	2.63e-01	1.54e-02	NA
178	E,A,V	5.68e-03	5.09e-01	6.66e-03	1.45e-03
196	W,Q,L	7.66e-05	8.45e-01	1.25e-02	7.58e-04
203	R,T	2.41e-03	7.35e-01	5.75e-02	2.38e-02
206	D,E	2.94e-01	4.85e-01	4.06e-01	2.75e-01
207	G,W	2.94e-01	4.85e-01	4.06e-01	2.75e-01
227	A,P	2.10e-03	3.42e-01	2.29e-03	5.06e-03
236	P,A	5.79e-03	6.67e-01	2.37e-02	2.35e-03
237	I,V	5.78e-03	6.67e-01	2.42e-02	2.39e-03
250	G,S	5.79e-03	6.67e-01	2.37e-02	2.35e-03
289	S,A	1.24e-09	5.32e-01	8.00e-02	1.70e-01
296	E,Q	5.66e-03	7.32e-01	2.34e-02	2.33e-03
319	L,P	4.86e-02	6.74e-01	6.38e-02	2.42e-02
325	V,I	3.06e-01	3.57e-01	2.56e-01	1.58e-01
337	L,F	5.04e-03	6.32e-01	2.23e-02	2.63e-03
341	F,I	3.00e-12	6.73e-01	7.60e-02	1.58e-01
342	A,T	5.51e-11	6.50e-01	1.03e-01	2.20e-01
350	R,M	3.00e-12	6.73e-01	7.60e-02	1.58e-01
354	N,K	3.06e-01	3.57e-01	2.56e-01	1.58e-01
364	T,S	2.59e-02	3.88e-01	1.33e-01	5.40e-02
377	M,V	3.13e-11	3.65e-01	1.97e-01	4.16e-01

Table S5: Association between HLA-A amino acid positions and HBV amino acid variants in the European cohort. P-values based on the omnibus test. The conditional p-value represents the association conditioned on the top association (in bold).

		Precore/core position 67 (Associated AA: Y)	
HLA-A Position	Possible AA	P	P (Conditional)
10	L,V	6.62e-10	4.21e-08
14	L,S	2.31e-08	5.94e-07
35	T,Y,S,F	1.70e-09	1.05e-07
91	G,R	6.37e-09	9.99e-08
92	K,N	4.41e-10	1.85e-08
96	Q,H	7.45e-09	1.82e-07
100	H,D	1.04e-10	4.19e-09
102	A,E,V	1.81e-08	1.41e-07
103	N,D	3.89e-08	3.98e-08
105	R,G	1.57e-09	1.30e-08
106	I,T	6.69e-10	1.20e-08
107	A,L	6.69e-10	1.20e-08
108	L,R	6.69e-10	1.20e-08
109	R,G	6.69e-10	1.20e-08
116	D,A	1.66e-10	2.03e-10
121	L,V,I	2.67e-10	1.06e-08
123	I,M,R	2.66e-10	1.28e-08
125	F,Y	1.26e-09	1.45e-08
131	P,S	1.18e-09	2.88e-09
133	W,G	1.25e-10	4.79e-09
140	Q,R,H	3.46e-07	1.51e-07
142	D,Y	1.24e-07	5.86e-08
153	N,K	6.66e-11	2.53e-09
168	T,I	1.42e-10	4.91e-09
170	Q,K	**5.86e-11**	NA
171	H,R	1.42e-10	4.91e-09
177	R,H	2.02e-09	6.42e-09
178	E,V	1.21e-07	5.86e-07
201	D,E	7.41e-10	2.32e-08
203	R,T	1.29e-08	1.45e-07
206	D,E	7.83e-09	1.28e-07
207	G,W	7.83e-09	1.28e-07
227	A,P	1.65e-09	1.25e-07
236	P,A	4.27e-09	2.44e-07
237	I,V	2.73e-09	1.37e-07
250	G,S	5.28e-09	2.76e-07
289	S,A	1.42e-08	1.17e-06
296	E,Q	3.26e-09	1.95e-07
319	L,P	3.82e-05	8.83e-04
325	V,I	6.87e-05	1.53e-03
337	L,F	4.25e-05	9.18e-04
341	F,I	8.56e-05	1.42e-03
342	A,T	8.45e-05	1.15e-03
350	R,M	8.56e-05	1.42e-03
354	N,K	6.87e-05	1.53e-03
364	T,S	3.82e-05	8.83e-04
377	M,V	8.15e-05	1.71e-03

Table S6: Interface template-modelling (ipTM) score and average interface predicted aligned error (PAE) scores of top-ranking models. Models were constructed based on wildtype (WT) NTCP or NTCP S267F, along with a peptide derived from the HBV genotype C wildtype (WT) preS1 sequence or with the introduction of associated preS1 mutations. Average interface PAE was calculated based on the mean across pairs of NTCP and preS1 residues (upper right box of PAE matrices, **Figure S12A and S12C**).

	**preS1 WT**	**preS1 S17A, G35R, H51P**	**preS1 S17A**	**preS1 G35R**	**preS1 H51P**
**NTCP WT**	ipTM: 0.34<br>PAE: 16.84	ipTM: 0.34<br>PAE: 17.25	ipTM: 0.27<br>PAE: 20.10	ipTM: 0.33<br>PAE: 20.16	ipTM: 0.34<br>PAE: 16.95
**NTCP S267F**	ipTM: 0.31<br>PAE: 18.87	ipTM: 0.31<br>PAE: 18.64	ipTM: 0.22<br>PAE: 21.12	ipTM: 0.3<br>PAE: 18.89	ipTM: 0.32<br>PAE: 18.38

Table S7: Population frequency of residues at position 17 of HBV preS1 (normalized to Genotype C) according to HBVdb. Asterisks indicate that the study the mutation (S17A) was observed included individuals with Asian ancestry in a country outside of Asia.

	Genotype A		Genotype B		Genotype C		Genotype D	
	**A**	**S**	**A**	**S**	**A**	**S**	**A**	**S**
**Australia and New Zealand**	0	1	**1***	24	**1***	29	0	34
**Central Asia**	0	4	0	0	0	2	0	18
**East Asia**	0	71	**24**	1901	**11**	1941	0	80
**Europe**	0	181	0	8	0	13	0	433
**Central and South America**	0	124	0	9	**1***	8	0	100
**Middle East and North Africa**	0	1	0	0	0	0	0	240
**North America**	0	470	**1**	315	0	523	0	137
**Polynesia and Micronesia**	0	0	0	0	0	9	0	5
**South Asia**	0	41	0	0	0	44	0	255
**South-East Asia**	**1**	10	**2**	373	**3**	293	0	7
**Sub-Saharan Africa**	0	263	0	0	0	2	0	89

Table S8: Predicted epitopes overlapping HBV amino acid positions. HLA-associated HBV amino acid positions are annotated in bold within the epitope sequences. Binding affinities are based on two algorithms (MixMHCpred and NetMHCpan). Experimental assay results are based on epitope sequence with exact match on IEDB (accessed June 2021).

Peptide Sequence	MixMHCpred EL Percentile Rank (%)	NetMHCPan IC50 (nM)	NetMHCpan EL Percentile Rank (%)	IEDB MHC Ligand Assays (# Positive / All)	IEDB T-Cell Assays (# Positive / All)
**Position 67 precore/core (G2G Association: HLA-A*01:01)**					
LLDTASAL**Y** (IEDB Epitope ID: 37181)	0.01	7.62	0.01	HLA-A*01:01 (1/1) HLA-A*02:01 (0/1)	Qualitative binding (2/2) IFNg release (1/1)
**Position 49 polymerase (G2G Association: HLA-A*02:06)**					
V**S**IPWTHKV	0.13	36.59	0.06	None	None
**Position 160 precore/core (G2G Association: HLA-A*33:03)**					
WIRTPP**A**YR (IEDB Epitope ID: 72649)	0.26	16.13	0.05	HLA-A*02:01 (0/1)	None

Supplementary Methods 1

Using AlphaFold-Multimer, a model was constructed for each combination: presence or absence of the NTCP S267F variant and the associated preS1 mutations. To quantify each model's support of preS1-NTCP binding, we focused on two metrics: the predicted interface template-modeling (ipTM) score and the predicted aligned-error (PAE) score. The ipTM score is a global accuracy measure of interface residues (between NTCP and preS1), with a higher value indicating higher accuracy and thus higher support of NTCP-preS1 binding. The PAE score is a per-residue accuracy metric between pairs of residues (x,y). The score refers to the expected position error at residue x if the predicted and true structures were aligned on residue y. We calculated the average PAE of the interface by including all residue pairs within the upper right box of the PAE matrices (**Figure S12A and S12C)**. Lower average PAE values within the interface indicate higher support of NTCP-preS1 binding. We did not include the lower left box of the PAE matrices to calculate the average PAE of the interface. This is because the pLDDT scores of preS1 residues, referring to the per-residue local confidence of the preS1 structure itself, were low (**Figure S12B and S12D)** and thus cannot be reliably aligned on.

First, we tested the independent effect of the NTCP S267F variant. Similar to the NTCP WT model, it is important to note that the prediction confidence for both the preS1-NTCP interface residues (**Figure S12C**) and the preS1 structure itself (**Figure S12D**) was also relatively low here. We compared the model of NTCP WT against the model of NTCP S267F, both in the presence of preS1 WT. By focusing on average PAE of the interface (dotted upper right box of **Figure S12A and S12C)**, we observed lower PAE in the NTCP WT model compared to the NTCP S267F model (**Table S6)**. A higher ipTM was also observed (**Table S6).** This corresponds to previous studies that establish the effect of NTCP S267F on inhibiting preS1 binding. Next, we tested the effects of associated preS1 mutations, either in the presence or absence of NTCP S267F. Focusing on models with NTCP S267F, the difference in PAE and ipTM in the presence or absence of all three associated preS1 mutations was minimal (**Table S6).** Minimal difference was also observed between models with NTCP WT. Given the relatively low overall confidence of the interface and preS1 structure, AlphaFold may not be sensitive enough to detect any changes in binding affinity caused by the associated preS1 mutations.

Supplementary Methods 2

Cell lines and maintenance

HepG2 and HEK293T cells were maintained in Dulbecco's Modified Eagle Medium (DMEM), supplemented with 10% FBS, 2 mM L-glutamine, 50 U/mL penicillin, 50 µg/mL streptomycin. For lentivirus production HEK293T cells were transfected using LT1 Transfection reagent with plasmid harboring WT NTCP and NTCP S267F variant both with a HA tag at the N terminus. HEK293T supernatant was collected from 24h to 48 h post transfection and ultracentrifuged for 2 hours at 20000 rpm. The lentivirus pellet was resuspended in DMEM medium and HepG2 cell transduced using 100 µl. NTCP expressing HepG2 were maintained under puromycin selection.

Taurocholate Uptake Assay

To measure the functionality of the stable expressed HA-NTCP a taurocholate uptake assay was performed. 24 hours after seeding, HepG2, HepG2 NTCP A3 clone (used as positive control), HepG2 HA-NTCP WT and HepG2 HA-NTCP S267F cells were incubated with taurocholate (150 mM containing 450 cpm/fmol [3H] taurocholate). Transport was stopped after 5 minutes by removing the medium and washing 3x with ice-cold PBS. Cells were lysed (0.2 M NaOH and 0.05% sodium dodecyl sulfate). Radioactivity of cell lysates was measured in a liquid scintillation counter (Packard Instruments, Frankfurt, Germany) using Ultima Gold liquid scintillation solution (Perkin Elmer, Rodgau, Germany).

Western Blot

Western blot analysis was performed on HepG2, HepG2 NTCP WT and HepG2 NTCP S267F cell lysates to quantify the expression level of NTCP. NTCP signals were detected using HA tag antibody (BioLegend Cat. 901502), quantified using Image Studio and normalized on the actin values.

Immunofluorescence

To assess NTCP cellular localization and expression after lentivirus transduction, HepG2, HepG2 NTCP WT and HepG2 NTCP S267F cells were fixed with 4% PFA and permeabilized at RT for 10 min. The cells were incubated with HA tag antibody (BioLegend Cat. 901502) in 2% BSA/PBS at RT for 1 hour. After washing with PBS, cells were incubated with 1:1000 diluted secondary antibody (goat anti rabbit Alexa Fluor 488) and 2 µg/mL Hoechst in the same solution as the secondary antibody for 1 hour at RT, protected from light. Imagines were acquired on fluorescence microscopy.

Synthesis of myristoylated peptides

WT and ARP myristoylated peptides were synthesized by solid phase peptide synthesis employing the Fmoc/tBu strategy with HBTU/DIPEA activation in an Applied Biosystems 433A peptide synthesizer. Atto565-maleimide was linked to the lysine (K) at position 59. The identity of the peptides was controlled by mass spectrometry.
