# Supplementary material for: Joint host-pathogen genomic analysis identifies hepatitis B virus mutations associated with human NTCP and HLA class I variation
Source: Am J Hum Genet. 2024 May 14;111(6):1018–34. doi: 10.1016/j.ajhg.2024.04.013 (PMC11179264; doi:10.1016/j.ajhg.2024.04.013)
Supplement: Document S2. Article plus supplemental information [file mmc2.pdf]

Joint host-pathogen genomic analysis identifies hepatitis B virus mutations associated with human *NTCP* and HLA class I variation

Graphical abstract

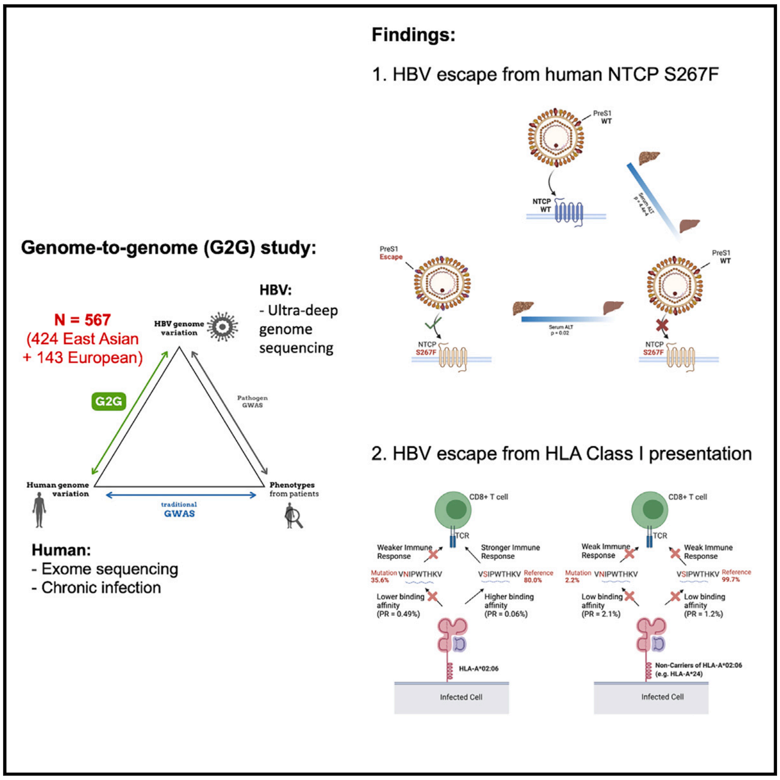

Authors

Zhi Ming Xu, Gnimah Eva Gnouamozi, Sina Rüeger, ..., David Gfeller, Stephan Urban, Jacques Fellay

Correspondence

jacques.fellay@epfl.ch

Host-induced selective pressure can shape viral evolution during chronic infections. By analyzing paired human and hepatitis B virus (HBV) genomic data, we pinpoint viral mutations associated with human NTCP and HLA-A variation, shedding light on HBV-evasion strategies that influence viral entry and HLA presentation.

Xu et al., 2024, The American Journal of Human Genetics *111*, 1018–1034
June 6, 2024 © 2024 The Author(s).
https://doi.org/10.1016/j.ajhg.2024.04.013

ARTICLE

Joint host-pathogen genomic analysis identifies hepatitis B virus mutations associated with human *NTCP* and HLA class I variation

Zhi Ming Xu,[1,2] Gnimah Eva Gnouamozi,[3] Sina Rüeger,[1,2] Patrick R. Shea,[4] Maria Buti,[5] Henry LY. Chan,[6] Patrick Marcellin,[7] Dylan Lawless,[1,2] Olivier Naret,[1,2] Matthias Zeller,[1] Arne Schneuing,[1] Andreas Scheck,[1] Thomas Junier,[1,2] Darius Moradpour,[8] Ondrej Podlaha,[9] Vithika Suri,[9] Anuj Gaggar,[9] Mani Subramanian,[9] Bruno Correia,[1] David Gfeller,[10,2] Stephan Urban,[3,11] and Jacques Fellay[1,2,12,*]

Summary

Evolutionary changes in the hepatitis B virus (HBV) genome could reflect its adaptation to host-induced selective pressure. Leveraging paired human exome and ultra-deep HBV genome-sequencing data from 567 affected individuals with chronic hepatitis B, we comprehensively searched for the signatures of this evolutionary process by conducting "genome-to-genome" association tests between all human genetic variants and viral mutations. We identified significant associations between an East Asian-specific missense variant in the gene encoding the HBV entry receptor NTCP (rs2296651, NTCP S267F) and mutations within the receptor-binding region of HBV preS1. Through *in silico* modeling and *in vitro* preS1-NTCP binding assays, we observed that the associated HBV mutations are in proximity to the NTCP variant when bound and together partially increase binding affinity to NTCP S267F. Furthermore, we identified significant associations between HLA-A variation and viral mutations in HLA-A-restricted T cell epitopes. We used *in silico* binding prediction tools to evaluate the impact of the associated HBV mutations on HLA presentation and observed that mutations that result in weaker binding affinities to their cognate HLA alleles were enriched. Overall, our results suggest the emergence of HBV escape mutations that might alter the interaction between HBV PreS1 and its cellular receptor NTCP during viral entry into hepatocytes and confirm the role of HLA class I restriction in inducing HBV epitope variations.

Introduction

Hepatitis B virus (HBV) infection is a major public health concern, with approximately 296 million chronically infected individuals as of 2019.[1] Chronic hepatitis B (MIM: 610424) leads to an increased risk of developing cirrhosis and hepatocellular carcinoma (HCC; MIM: 114550).[2] In 2019, 820,000 people died from HBV infection-related causes, mostly from cirrhosis and HCC.[1]

The human genetic basis of the susceptibility to HBV infection and the progression to or prognosis of chronic hepatitis B have been explored in numerous studies. Genome-wide association studies (GWASs) and candidate gene studies have had success in pinpointing specific loci associated with outcomes of HBV infection.[3] Many of the identified variants map to immune-related genes, including human leukocyte antigen (HLA) class I and II,[4,5] cytokines,[6–8] and Toll-like receptors.[9,10] Variants within non-immune-related genes have also been identified. In particular, an Asian-specific single-nucleotide polymorphism (SNP) (rs2296651), which encodes an amino-acid change (S267F) in the sodium taurocholate co-transporting polypeptide (NTCP; MIM: 182396), the entry receptor of HBV, has been associated with reduced susceptibility to HBV infection and more favorable clinical outcomes.[11–19]

A limitation of using GWASs to study infectious disease is the failure to consider the genetic variability of the pathogen. Because host and pathogen genetic determinants jointly determine the fitness of the pathogen and the prognosis of the disease, evolutionary changes occur on both genomes as a result of an "arms-race"-like evolutionary process.[20] Selective pressure originating from host polymorphisms can induce escape mutations on the pathogen genomes, and on a much longer timescale, host polymorphisms that counteract these escape mutations could undergo positive selection at a population level. For viruses with a high mutation rate, intra-host evolution resulting from host genetic pressure has been shown to occur during chronic infections. For example, during HIV (MIM: 609423) infection, longitudinal studies have provided direct evidence of immune evasion in the form of

[1]School of Life Sciences, École Polytechnique Fédérale de Lausanne, Lausanne, Switzerland; [2]Swiss Institute of Bioinformatics, Lausanne, Switzerland; [3]Department of Infectious Diseases, Molecular Virology, University Hospital Heidelberg, Heidelberg, Germany; [4]Institute for Genomic Medicine, Columbia University, New York, NY, USA; [5]Liver Unit, Hospital Universitario Vall d'Hebron and CIBEREHD del Instituto Carlos III, Barcelona, Spain; [6]The Chinese University of Hong Kong, Hong Kong, China; [7]Service d'Hépatologie, Hôpital Beaujon, Clichy, France; [8]Division of Gastroenterology and Hepatology, Lausanne University Hospital and University of Lausanne, Lausanne, Switzerland; [9]Gilead Sciences Inc, Foster City, CA, USA; [10]Department of Oncology UNIL-CHUV, Lausanne University Hospital, Ludwig Institute for Cancer Research, University of Lausanne, Lausanne, Switzerland; [11]German Center for Infection Research (DZIF), Partner Site Heidelberg, Heidelberg, Germany; [12]Precision Medicine Unit, Biomedical Data Science Center, Lausanne University Hospital and University of Lausanne, Lausanne, Switzerland
*Correspondence: jacques.fellay@epfl.ch
https://doi.org/10.1016/j.ajhg.2024.04.013.
© 2024 The Author(s). This is an open access article under the CC BY license (http://creativecommons.org/licenses/by/4.0/).

escape mutations in HIV epitopes targeted by cytotoxic T lymphocytes (CTLs).[21–25] Although HBV is a DNA virus, it has a mutation rate that is closer to that of RNA viruses than to other DNA viruses[26] due to an error-prone reverse-transcription step in its replication cycle. As such, evidence of intra-host evolution and its role in the development of HCC has been observed for HBV.[27] Cross-sectional studies have also supported the presence of CTL escape during HBV infection.[28–31]

In this study, we leveraged paired human exome and ultra-deep HBV genome-sequencing data from a cohort of 567 affected individuals with chronic hepatitis B. We utilized a "genome-to-genome" (G2G) approach[32–34] where we performed a genomes-wide search for associations between all pairs of HBV amino-acid mutations and human SNPs while correcting for confounding factors including human and viral population structures. We identified HBV mutations that are associated with human genetic variations, reflecting host-induced selective pressure on the virus. Our results support the role of HLA class I variation in inducing HBV epitope variation and pinpoint potential escape mutations within HBV preS1 that alter preS1-NTCP interaction, which is a prerequisite for HBV internalization.

Material and methods

Study description

Paired human blood and pre-treatment HBV DNA samples were obtained from affected individuals with chronic hepatitis B enrolled in four phase three clinical trials that evaluated tenofovir-based antiviral regimens (GS-US-283-1062,[35] GS-US-320-0108,[36] GS-US-320-0110,[37] and GS-US-330-1401[38]) conducted by Gilead Sciences. Individuals with East Asian ancestry were recruited from Australia (AUS) ($N = 6$), Canada (CAN) ($N = 80$), Spain (ESP) ($N = 1$), France (FRA) ($N = 1$), Great Britain (GBR) ($N = 2$), Hong Kong (HKG) ($N = 94$), Italy (ITA) ($N = 1$), Japan (JPN) ($N = 32$), South Korea (KOR) ($N = 117$), New Zealand (NZL) ($N = 15$), Russia (RUS) ($N = 2$), Singapore (SGP) ($N = 4$), and the United States (USA) ($N = 69$). Individuals with European ancestry were recruited from AUS ($N = 2$), CAN ($N = 1$), ESP ($N = 1$), GBR ($N = 3$), ITA ($N = 22$), NZL ($N = 1$), Poland (POL) ($N = 20$), Romania (ROU) ($N = 34$), RUS ($N = 44$), Turkey (TUR) ($N = 11$), and USA ($N = 4$). The trials excluded individuals with evidence of HCC, cirrhosis/hepatic decompensation, and co-infections with hepatitis C virus (HCV), hepatitis D virus (HDV), or HIV. Biomarkers of HBV infection including HBV viral load, hepatitis B surface-antigen (HBsAg) level, hepatitis B e antigen (HBeAg) status, and serum alanine aminotransferase (ALT) level were measured at baseline. Across the four trials, both HBeAg-positive and HBeAg-negative individuals were included. All study participants signed an informed consent form prior to screening and in accordance with local regulatory and ethics committee requirements. A summary of the demographical details of each study can be found in Table S1. Complete details can be found at https://clinicaltrials.gov/ (ClinicalTrials: NCT02579382, NCT01940341, NCT01940471, and NCT02174276).

Exome sequences

High-quality genomic DNA was extracted from frozen peripheral blood samples using the Beckman Biomek FXp liquid handling system. Enrichment of the exonic regions of the genome was performed using Roche or IDT Exome Research Panel V1 capture kits (Integrated DNA Technologies, Coralville, IA USA). Short-read DNA sequencing was performed using Illumina HiSeq2500 or NovaSeq 6000 sequencers according to the manufacturer's instructions. Initial quality-control filtering of the exome-sequencing reads, adapter masking, and conversion of BCL files to fastq format was performed using CASAVA software version 1.8 (https://support.illumina.com/sequencing/sequencing_software/bcl2fastq-conversion-software.html).

Sequencing reads were aligned to the GRCh37.87 (hs37d5.fa; https://ftp.1000genomes.ebi.ac.uk/vol1/ftp/technical/reference/phase2_reference_assembly_sequence/hs37d5.fa.gz) reference genome using the DRAGEN Bio-IT platform (https://support.illumina.com/sequencing/sequencing_software/dragen-bio-it-platform.html) v.2.0.1. Duplicate sequencing reads were removed using Picard (https://broadinstitute.github.io/picard/) v.2.2.1. Variant calling was performed with the Genome Analysis Tool Kit (GATK) HaplotypeCaller program (https://gatk.broadinstitute.org/hc/en-us) v.v3.6-0-g89b7209 according to GATK Best Practice guidelines[39] using dbSNP v138 (https://www.ncbi.nlm.nih.gov/snp/). Local re-alignment of all insertion/deletions variants was performed using Smith-Waterman, and GATK Base Quality Score Recalibration was applied to single nucleotide variants (SNVs).

Quality control

Poor-quality samples and variant calls were excluded using PLINK (https://www.cog-genomics.org/plink/) v.1.9[40] by applying a sample-based and variant-based missingness threshold of 0.1. Variants that deviate from Hardy Weinberg equilibrium were also excluded by applying a filtering threshold of $p < 1 \times 10^{-6}$. Related individuals up to first-degree relatives were excluded using the KING software (https://www.kingrelatedness.com/) v.2.2.2.[41]

Human genetic principal components

To obtain genetically determined ancestry of study participants, principal-component analysis (PCA) was first applied to the 1000 Genomes (1KG) reference samples (https://www.internationalgenome.org/data).[42] Using the GCTA software v.1.91.7,[43] PCA loadings were extracted from the 1KG samples and then applied to the genotypes of our study participants. We then compared self-reported race and genetically determined ancestry. We observed that most of those who self-report as White were most genetically similar to the European-ancestry group, while most of those who self-report as Asian were most genetically similar to the East Asian-ancestry group (Figure S1). To assign genetically determined ancestry labels, hierarchical clustering was applied using the Wald method based on Euclidean distances in the PC space. The dendrogram was cut such that study participants were clustered into two major ancestry groups (East Asian and European) and a separate group representing mostly South Asian ancestry. Participants assigned to the East Asian- and European-ancestry group, with self-reported race of Asian and White, respectively, were retained. Participants in other ancestry groups were excluded given the limited sample size with constrained statistical power to detect associations. PCs were then re-calculated for the retained participants to be used in subsequent analyses. We refer to participants within these two ancestry groups as the East Asian and European cohort, respectively.

Imputation of HLA alleles and amino acid variants

The HLA-LA[44] software (https://github.com/DiltheyLab/HLA-LA) was used to impute HLA alleles at the four-digit levels based on

BAM files that included unaligned reads. A quality threshold (Q1 > 0.7) was applied to retain high-quality HLA allele inferences. To encode amino-acid variants of HLA, the PyHLA[45] software (https://github.com/felixfan/PyHLA) was used to identify amino-acid changes associated with each HLA allele. HLA alleles and HLA amino-acid variants were then encoded as dosages (presence or absence), and Variant Call Format (VCF) files were constructed for subsequent analyses.

Viral genome sequences

Viral genome sequencing and mapping

DNA isolation was performed using Qiagen MinElute kit for serum samples with viral load <100,000 IU/mL and Roche MagNA Pure robot 32 for samples with viral loads >100,000 IU/mL. The amplification of HBV whole genome was performed by DDL Diagnostics Laboratory (Rijswijk, Netherlands) following the modified protocol described in Gunther et al.[46] Whole-genome HBV amplicons were sequenced on Illumina MiSeq with 150 bp paired-end reads. Low-quality bases ($Q < 20$) at 5′ and 3′ of each read were trimmed with Trimmomatic software (https://github.com/usadellab/Trimmomatic) v.0.35,[47] and reads shorter than 50 bp were removed. Subsequently, paired reads were merged based on overlapping regions, and sequencing error correction was performed using PEAR software (https://cme.h-its.org/exelixis/web/software/pear/) v.0.9.6. Unmerged reads were discarded. Read mapping was performed with BWA software (https://bio-bwa.sourceforge.net/) v.0.7.9a, and the reference genome for each sample was chosen from HBVdb.ibcp.fr (https://hbvdb.lyon.inserm.fr/HBVdb/HBVdbIndex)[48] given the patient's genotype, which was determined via laboratory genotyping assay. NCBI accession numbers (https://www.ncbi.nlm.nih.gov/nuccore) are as follows: genotype A, NCBI: EU054331; genotype B, NCBI: AB219428; genotype C, NCBI: GQ924620; genotype D, NCBI: FJ904433; genotype F, NCBI: AY090458; and genotype H, NCBI: FJ356716. On average, a genome-wide coverage of approximately 6,800× was achieved per sample: 337 samples exceeded 7,000×, 202 samples between 5,000× and 7,000×, and 28 samples between 2,000× and 5,000×.

Viral mutations

Viral genome positions were normalized to the HBV genotype C (NCBI: GQ924620). At a given genome position, a nucleotide was treated as present if it exceeded the minimal intra-host frequency of 15%. In cases where multiple nucleotides were detected above minimal frequency, International Union of Pure and Applied Chemistry (IUPAC) coding was implemented. Minimum sequencing coverage of 100 reads was required to record a nucleotide call at each genome position.

Amino-acid mutation matrix was generated using an analogous strategy requiring a minimum sequencing coverage spanning the entire codon length. Because the HBV genome contains overlapping reading frames, each open reading frame was covered independently of others.

Phylogenetic tree

The Woolly Monkey HBV (GenBank: NC_028129.1) nucleotide sequence was used as an outgroup for the construction of the phylogenetic tree. The Woolly Monkey sequences were aligned to the HMM profile built by the multiple-sequence alignment of HBV nucleotide sequences from our study using hmmbuild of HMMER software (http://hmmer.org/) v.3.2.1. The maximum-likelihood-based phylogenetic tree was inferred using the IQ-TREE software (http://www.iqtree.org/) v.1.16.12,[49] where the best model within the general time-reversible (GTR) model family was selected as the nucleotide substitution model. Branch support bootstrap values were calculated using UFBoot[50] with 1,000 replicates. In general, HBV genotypes were monophyletic, and the tree topology corresponded to our current understanding of evolutionary relationships between HBV genotypes,[51] suggesting the validity of the inferred phylogeny (Figure S2).

Phylogenetic PCs

To capture HBV amino-acid variations that are correlated with phylogenetic structure and to summarize such variation as synthetic variables, phylogenetic PCA (pPCA) was applied to HBV amino-acid dosages (presence or absence). Similar to PCA, pPCA is a dimensional reduction technique where orthogonal synthetic variables that capture most of the variance are constructed. However, unlike standard PCs, pPCs applied to amino acids are constrained such that variations that are correlated with the phylogenetic structures are captured. The ppca function within the *adephylo* package (https://cran.r-project.org/web/packages/adephylo/index.html) v.1.1–11 in R, which implements the pPCA formulation as described by Jombart et al.,[52] was utilized. Abouheif's proximity was selected as the phylogenetic proximity metric. Because pPCs are constrained by phylogenetic structures, they were able to accurately discern HBV genotypes as expected (Figure S3).

Association studies

For G2G association studies, our analyses were restricted to common human SNPs (minor allele frequency >0.05) and common HBV amino-acid mutations (minor allele count >20) for which the model was able to reach convergence. Analyses were conducted separately in the East Asian and European cohort to avoid spurious associations driven by population stratification given that HBV genotypes and variation are highly correlated with patient ancestry (Table 1). This resulted in the inclusion of 164,204 human SNPs with 442 HBV amino-acid mutations in the East Asian cohort and 196,545 human SNPs with 88 HBV amino-acid mutations in the European cohort. HBV amino-acid mutations with perfect correlation ($r^2 = 1$), which include mutations at the same amino-acid position or mutations in overlapping reading frames driven by the same nucleotide mutations, were pruned. The number of effective tests (N_{eff}) was determined to be 433 and 88 in the East Asian and European cohort, respectively. To account for multiple testing, the Bonferroni correction threshold was derived by dividing the genome-wide significance threshold (5×10^{-8}) by the total number of effective tests ($N_{eff} = 521$), resulting in a threshold of 9.6×10^{-11}.

Each retained HBV amino-acid mutation was encoded as binary outcomes, representing either presence above 15% in the intra-host population or absence. A GWAS was conducted for each HBV amino-acid variant as the outcome using the generalized linear mixed model implemented by SAIGE (v.0.35.8)[53] in R. Age, sex, the top four human PCs (8.3% of variance explained), and the top six pathogen pPCs (87.8% of variance explained) were included as covariates. p values based on the saddle point approximation (SPA) test implemented by SAIGE software (https://saigegit.github.io/SAIGE-doc/) v.0.35.8[53] are reported. For associations involving rs2296651, due to the unreliability of effect-size estimates based on the SPA test for variants with low minor allele counts, odds ratios (ORs) were calculated using Firth logistic regression implemented by PLINK v.1.9.

To confirm the absence of genomic inflation in the GWAS with significant genome-wide association(s), we constructed quantile-quantile (QQ) plots and calculated genomic inflation factors (λ), which demonstrated the absence of inflation (Figures S4 and S5).

Table 1. Baseline characteristics

	East Asian (*N* = 424)	European (*N* = 143)
**Age**		
Median (range)	42 (18–69)	38 (18–73)
**Sex**		
Female	157 (37%)	47 (32.9%)
Male	267 (63%)	96 (67.1%)
**HBV genotype**		
A	7 (1.65%)	24 (16.8%)
C	310 (73.1%)	3 (2.1%)
D	5 (1.18%)	111 (77.6%)
F	0 (0%)	3 (2.1%)
H	0 (0%)	2 (1.4%)
B	102 (24.1%)	0 (0%)
**HBeAg**		
Negative	170 (40.1%)	73 (51%)
Positive	254 (59.9%)	70 (49%)

Baseline characteristics and HBV genotypes of study participants, grouped according to genetically determined ancestry.

For G2G associations involving HLA amino-acid mutations, which could be multiallelic, the GWASs were based on omnibus tests where the –hap-snps and –chap flag of PLINK (https://zzz.bwh.harvard.edu/plink/) v.1.07 was used to group amino-acid residues at the same position. For associations involving HLA alleles, the SPA test implemented by SAIGE was used to calculate the p values and ORs under a dominant model. For the conditional analyses, the top association (either SNP, amino acid, or allele) was conditioned on using the –cond command of PLINK v.1.9.

Inverse normal transformations were applied to biomarkers of HBV infection (HBsAg levels, serum ALT levels, and viral load). HBV nucleotide mutations associated with biomarkers of HBV infection were identified using linear regression, adjusting for HBeAg status, age, sex, the top four human PCs, and the top six pathogen pPCs. HBV nucleotide mutations associated with HBeAg status were identified using logistic regression, adjusting for age, sex, the top four human PCs, and the top six pathogen pPCs.

For stratified analyses involving rs2296651 genotype and associated HBV preS1 escape haplotypes, individuals were separated into groups according to human genotype and the presence or absence of HBV escape haplotypes. Pairwise comparisons were made between groups. Using multiple linear regression, each biomarker of HBV infection was regressed against the group indicator (binary 0 or 1), HBeAg status, age, sex, the top four human PCs, the top six pathogen pPCs, and all nucleotide mutations significantly associated with each marker of infection (Figure S6). Nucleotide mutations significantly associated with each marker of infection were clumped ($r^2 < 0.8$) based on the strength of association, such that only independent mutations were incorporated in the regression model.

In silico model of preS1-NTCP binding

To construct an *in silico* model of preS1-NTCP binding, the ColabFold[54] (https://colab.research.google.com/github/sokrypton/ColabFold/blob/main/AlphaFold2.ipynb) implementation of AlphaFold-Multimer was used. The developers of AlphaFold-Multimer have shown that the method has the ability to predict protein-peptide complexes when the protein and peptide are encoded as separate chains.[55] Here, we encoded the preS1-derived peptide (amino acids 2–60 of preS1) and NTCP protein as separate chains. We relied on an *in silico* approach because existing cryoelectron microscopy (cryo-EM) data[56] did not offer sufficient resolution to model the interface. Nevertheless, the cryo-EM structure of NTCP in the presence of preS1 (PDB: 7VAG) was used as a template to improve the *in silico* predictions. Default settings of ColabFold were used, and the number of recycles was set at three. Images of predicted structures were produced using the UCSF Chimera package (https://www.cgl.ucsf.edu/chimera/)[57] from the Resource for Biocomputing, Visualization, and Informatics at the University of California, San Francisco.

The amino-acid sequence of NTCP that was used was identical to that of the structure (PDB: 7VAG) excluding missing residues. The preS1 sequence was based on the genotype C consensus sequences derived from the cohort, stratified based on NTCP genotype (NTCP wild type [WT] or S267F).

PreS1 haplotype calling and intra-host nucleotide diversity

To assemble sequencing reads into intra-host preS1 haplotypes, the shorah software (https://github.com/cbg-ethz/shorah)[58] was used. The shotgun-based local analysis was selected because the region of interest (first 177 bp of preS1) was slightly longer than the average read length (150 bp). Low-confidence haplotypes (posterior <0.95 or supporting reads <10) were filtered out. Nucleotide haplotypes were translated into amino-acid sequences using the seqinr package (https://cran.r-project.org/web/packages/seqinr/index.html)[59] in R. A multiple-sequence alignment with 1,000 sequences was constructed for each patient by up-sampling each haplotype based on its intra-host proportion. Sequence logos that represent the overall intra-host composition across all samples were then constructed based on merging multiple-sequence alignment across all samples and using the ggseqlogo package (https://omarwagih.github.io/ggseqlogo/)[60] in R.

To estimate the magnitude and infer the type of intra-host selection occurring within preS1, we calculated the ratio of non-synonymous to the synonymous nucleotide diversity (π_N / π_S). The π_N / π_S ratio is analogous to the d_N / d_S ratio but is weighted according to the composition of intra-host viral population.[61] To incorporate the effect of overlapping reading frames, the mean pairwise number of substitutions per site that are non-synonymous in both frames (π_{NN}) and that are synonymous in preS1 but non-synonymous in polymerase (π_{SN}) were calculated using OLGenie (https://github.com/chasewnelson/OLGenie)[62] based on the up-sampled multiple-sequence alignment. Non-synonymous (π_N) and synonymous nucleotide (π_S) diversity ignoring the overlapping reading frame was also calculated using the same approach.

Population frequency of HBV preS1 amino-acid mutations

To estimate the population frequencies of HBV preS1 amino-acid mutations, amino-acid multiple-sequence alignments for the large surface protein were downloaded separately for each HBV genotype (A, B, C, D) from HBVdb.ibcp.fr.[48] Positions were normalized to genotype C (NCBI: GQ924620). The Entrez batch query tool (https://www.ncbi.nlm.nih.gov/sites/batchentrez) was used to extract the "country" field from the metadata for each HBV sequence hosted

on the NCBI Nucleotide Database (https://www.ncbi.nlm.nih.gov/nucleotide/). Countries were subsequently grouped into geographical regions.

In vitro preS1-NTCP binding assays

We synthesized the 59-amino-acid myristoylated peptides derived from the most common genotype C preS1 haplotypes observed in either NTCP WT carriers or NTCP S267F carriers:

NTCP S267F (Myr-ARP):

Myr-MGGWSSKPRKGMGTNL**A**VPNPLGFFPDHQLDLAF**R**ANSNNPDWDFNPNKD**P**WPEANQVG

NTCP WT (Myr-WT):

Myr-MGGWSSKPRQGMGTNL**S**VPNPLGFFPDHQLDPAF**G**ANSNNPDWDFNPNKD**H**WPEANQVG

The variable amino acids at positions 17, 35, and 51 of the preS1 region are highlighted in bold. For binding assay experiments, HepG2, HepG2 HA-NTCP WT, and HepG2 HA-NTCP S267F were aliquoted at a concentration of 2×10^5 per Eppendorf tube. The cells were pelleted for 3 min at 100 × g, and the pellet was resuspended in appropriate peptide dilution. Peptides were applied at 0, 15, 50, 200, and 1,000 nM, and Bulevirtide (Myrcludex B) was added as side binding control. Cells were incubated at room temperature for 30 min in the dark and then pelleted by centrifugation for 3 min at 100 × g and washed five times with PBS. The washed cells were resuspended in 500 μL of FACS buffer (0.5% BSA and 0.02% sodium azide in 1× PBS), and fluorescence was measured by flow cytometry using Cell Sorter BD FACS Celesta. For immunofluorescence readout of the binding assay, cells were seeded in 24-well plate (3×10^5/well) and 24 h later incubated with peptides as performed for fluorescence-activated cell sorting (FACS) analysis. After incubation, cells were washed with 2% BSA/PBS and fixed with 1.25% paraformaldehyde. Atto 565 signal was detected at fluorescence microscope. Further details can be found in Supplementary Methods S2.

HLA peptide-binding predictions

To predict peptides restricted by specific HLA class I alleles and their respective binding affinities or elution probabilities, the NetMHCpan4.0 (http://tools.iedb.org/mhci/download/)[63] and MixMHCpred2.2 (https://github.com/GfellerLab/MixMHCpred)[64] software were used.

For each HBV amino-acid position associated with a human HLA allele, we obtained flanking sequences based on the HBV genotype C reference sequence (NCBI: GQ924620). Next, for each HBV amino-acid position and HLA-allele pair, we used a sliding window approach where we predicted the binding affinities for all possible 9- to 14-mers peptides to identify the most likely peptide restricted by the HLA allele. We chose to include all 9–14 mers as they are the most likely peptide lengths for the HLA class I alleles of interest (Figure S7). For each HLA allele and HBV variant pair, the peptide with the lowest NetMHCpan predicted elution percentile rank was chosen as the putative peptide.

Results

Study description

Table 1 summarizes the baseline characteristics of the study participants. Based on the consensus between self-reported and genetically determined ancestry (Figure S1), we separated our study into an East Asian cohort ($N = 424$) and a European cohort ($N = 143$). Individuals within the East Asian cohort were predominantly infected with HBV genotype B ($N = 310$; 73%) and C ($N = 102$; 24%), while those within European cohort were predominantly infected with HBV genotype A ($N = 111$; 78%) and D ($N = 24$; 17%). Both HBeAg-positive ($N = 243$; 43%) and HBeAg-negative ($N = 324$; 57%) participants were included in the study.

Association study between human and HBV genetic variation

To identify associations between human SNPs and HBV amino-acid mutations, which we refer to as G2G associations, we conducted multiple GWASs where each HBV amino-acid variant was encoded as a binary outcome (presence above 15% in the intra-host viral population or absence). The GWASs were conducted separately in two cohorts, including participants with East Asian and European ancestry, respectively. Human and pPCs were included as covariates to correct for population stratification.

We found that variants mapping to two regions of the human genome were significantly associated with HBV amino-acid variation: the *SLC10A1* gene region on chromosome 14 and the HLA class I region on chromosome 6. (Figure 1).

On chromosome 14, an SNP in the *SLC10A1* (MIM: 182396) gene (rs2296651), which encodes for an amino-acid change (S267F) on the HBV entry receptor NTCP, showed significant association with three amino-acid mutations within the preS1 domain of the HBV large-surface protein (Table 2). According the gnomAD, rs2296651 is a variant that is prevalent in East Asian populations but virtually absent in all others. Correspondingly, the associations were only observed in the East Asian cohort. The strongest association was between rs2296651 and position 17 of preS1 (associated amino acid: A; $p = 1.7 \times 10^{-12}$; OR = 179.2), along with other significant associations with position 35 (associated amino acid: R; $p = 3.8 \times 10^{-11}$; OR = 36.5) and position 51 (associated amino acid: P; $p = 1.9 \times 10^{-12}$; OR = 88.9). A suggestive association was observed between rs2296651 and the position 32 of preS1 (associated amino acid: L; $p = 1.5 \times 10^{-10}$; OR = 126.7). To confirm that the significant associations were not genotype specific, we conducted a stratified analysis within HBV genotypes B and C in the East Asian cohort (Table 2). Genotypes A and D were excluded given their low prevalence in the East Asian cohort. The direction of associations was identical across genotypes, and effect sizes were similar across genotypes for position 17 and position 35 while less strong for position 51 in genotype B. Due to the overlapping reading frames of HBV, rs2296651 also showed significant association with position 197 of the polymerase protein (associated amino acid: C; $p = 6.9 \times 10^{-12}$; OR = 181.8) and suggestive association with position 215 of the polymerase protein (associated amino acid = Q; $p = 2.8 \times 10^{-9}$; OR = 27.5). The associated polymerase mutations reflect the same variation at the nucleotide level as the associated preS1 mutations.

On chromosome 6, SNPs mapped to the *HLA-A* (MIM: 142800) gene within the HLA class I region showed

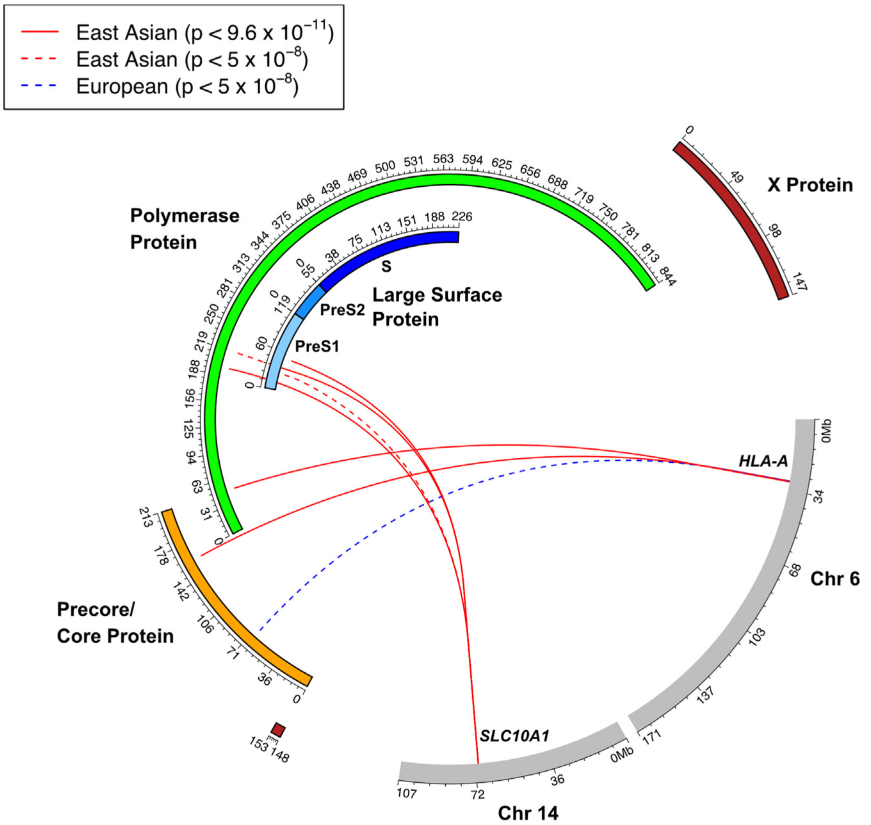

Figure 1. Genome-to-genome analysis
Associations between human SNPs and HBV amino-acid mutations. Gray panels represent human chromosomes and respective nucleotide positions. Colored panels represent HBV proteins and respective amino-acid positions. Solid lines represent significant associations under Bonferroni correction ($p < 9.6 \times 10^{-11}$), and dashed lines represent genome-wide significant associations ($p < 5.0 \times 10^{-8}$). Red lines represent associations within the East Asian cohort, and blue lines represent associations within the European cohort.

significant associations (top association: $p = 8.4 \times 10^{-14}$) with two HBV amino-acid mutations in the East Asian cohort (Figures 2A and 2B) and suggestive associations (top association: $p = 1.9 \times 10^{-8}$) with an HBV amino-acid variant in the European cohort (Figure 2C). Because the associated SNPs could be tagging functional variants at the HLA amino-acid or allele level, we imputed the four-digit HLA alleles and HLA amino acids, which we used in additional association analyses (Figure 2; Tables S2–S5). In the East Asian cohort, HLA-A T99I ($p = 2.9 \times 10^{-13}$; OR = 0.07) and HLA-A*33:03 ($p = 2.1 \times 10^{-10}$; OR = 0.08) were associated with position 160 of the HBV precore/core protein (associated residue: A). In addition, HLA-A Y35T ($p = 5.8 \times 10^{-5}$; OR = 0.33) and HLA-A*02:06 ($p = 1.3 \times 10^{-12}$; OR = 84.2) were associated with position 49 of the HBV Pol (associated residue: N). In the European cohort, HLA-A K170Q ($p = 5.9 \times 10^{-11}$; OR = 2.95) and HLA-A*01:01 ($p = 9.7 \times 10^{-8}$; OR = 0.07) were associated with position 67 of HBV precore/core protein (associated residue: Y). To evaluate whether a separate signal might be present outside of the *HLA-A* gene, we conducted conditional analyses based on the top association within *HLA-A* (Figure 2). We did not observe any significant associations outside of *HLA-A* in these conditional analyses. Given that HLA-allele frequencies are highly variable among human populations, we also investigated whether the associations were restricted to certain ancestry subgroups within the East Asian and European cohorts. PCA revealed that the HLA alleles and the associated HBV mutations were present in multiple ancestry subgroups within the East Asian or European cohort (Figures S8 and S9), and their prevalences were not strongly correlated with any human or phylogenetic PCs ($r^2 < 0.05$) (Figure S10).

NTCP and HBV preS1 variations

Because it has been established that the interaction between the receptor-binding region of the HBV preS1 and NTCP is essential for viral entry,[65,66] we used AlphaFold-Multimer[55] to construct *in silico* models of binding that allowed us to evaluate the proximity between the human NTCP variant (rs2296651, NTCP S267F) and associated HBV preS1 mutations when bound. We first constructed a model based on the human WT NTCP and a preS1 peptide (amino acids 2–60) derived from the HBV genotype C consensus sequence. Consistent with cryo-EM densities, the top-ranking model indicated binding of the preS1 peptide to the extracellular opening of the NTCP tunnel that is also responsible for substrate transport (Figures S11A and S11B). According to the *in silico* model, the NTCP S267 variant is located within the tunnel and is in proximity to S17 and G35 of preS1 when bound (Figure S11A). S17 of preS1 is the most proximal residue to S267 of NTCP at 9.5 Å. G35 of preS1 is 16.2 Å to S267 of NTCP. H51 of preS1 is 9.4 Å to patch 1 of NTCP (84–87), which has been reported to include residues essential for preS1 binding.[67] To study the impact of the NTCP S267F on the binding of preS1 peptides that encode or do not encode the mutations associated with the human NTCP variant, we also constructed separate models for every combination (Supplementary Methods S1, Table S6). Given that the prediction confidence for both the preS1-NTCP interface (Figure S12A) and the preS1 peptide structure itself (Figure S12B) was relatively low, conclusions that could be drawn from this *in silico* analysis were limited.

To characterize the effect of the human NTCP variant on the intra-host diversity of the preS1 receptor-binding region, we stratified patients according to rs2296651 genotypes and constructed sequence logos that represent the overall intra-host composition. Figures 3A and 3B illustrate

Table 2. Association between rs2296651 and HBV preS1 mutations in the East Asian cohort

preS1 Position	Cases: associated amino acid (%)	Controls: ALT amino acid	rs2296651 MAF (controls:cases)	p	OR	OR 95% CI
**All HBV genotypes**						
17	A (5%)	S	0.031:0.48	1.69×10^{-12}	179.2	30.4–1055.4
35	R (9.9%)	E, G, K	0.025:0.31	3.83×10^{-11}	36.5	12.4–107.3
51	P (6.6%)	H, K, N, Q, R, T	0.025:0.45	1.87×10^{-12}	88.9	23.4–336.9
**HBV genotype C**						
17	A (5.1%)	S	0.029:0.47	5.94×10^{-8}	118.6	16.7–842.3
35	R (11.9%)	E, G, K	0.016:0.31	5.61×10^{-10}	28.9	9.1–91
51	P (8.7%)	H, Q	0.014:0.44	6.74×10^{-13}	78.7	18.8–329.4
**HBV genotype B**						
17	A (4.9%)	S	0.031:0.5	2.12×10^{-3}	53.0	5.1–551.4
35	R (2.9%)	K	0.04:0.5	NA	25.5	2.6–249.9
51	P (1%)	K, N, T	0.05:0.5	NA	5.1	0.5–48.1

Samples with the presence (exceeding intra-host frequency of 15%) and absence of the associated HBV amino acid were encoded as cases and controls, respectively. Minor-allele frequency (MAF) ratio represents the MAF of rs2296651 in the cases compared to controls. Odds ratio (OR) is based on firth logistic regression assuming an additive model based on the minor allele (A) of rs2296651. *p* value is based on the SPA test, with NA indicating that the algorithm failed to converge due to low minor allele count.

the intra-host composition in genotype C-infected individuals who are heterozygous NTCP S267F carriers (rs2296651-G/A) and NTCP WT carriers (rs2296651-G/G), respectively. Figures 3C and 3D illustrate the intra-host composition in genotype B-infected individuals who are heterozygous NTCP S267F carriers (rs2296651-G/A) and NTCP WT carriers (rs2296651-G/G), respectively. For both HBV genotypes, we observed that preS1 sites associated with rs2296651 (marked by asterisks) were highly conserved in rs2296651-G/G individuals but showed increased diversity in rs2296651-G/A individuals, suggesting intra-host selection induced by the NTCP variant. To quantify the magnitude and direction of intra-host selection within the entire preS1 receptor-binding region, we compared the non-synonymous nucleotide diversity (π_N) against the synonymous nucleotide diversity (π_S) in rs2296651-G/A and rs2296651-G/G individuals. Compared to the d_N/d_S ratio, the π_N/π_S ratio is weighted according to the intra-host composition of viral populations but similarly reflects positive selection if greater than 1, neutral selection if equal to 1, and purifying selection if less than 1.[61] No significant differences were observed in π_S between rs2296651-G/G and rs2296651-G/A individuals, but higher π_N was observed in rs2296651-G/A carriers (Figure S13A). This contributed to a higher π_N/π_S ratio in rs2296651-G/A individuals, indicating increased positive selection. We observed similar results when we controlled for the effect of the overlapping reading frames by restricting to only substitutions and sites that are non-synonymous in the polymerase protein (Figure S13B).

Because the HBV preS1 sites that are associated with the human NTCP variant are in high linkage disequilibrium, we next assembled ultra-deep HBV sequencing reads (average coverage of 6,045× in the receptor-binding region) into intra-host haplotypes. We grouped haplotypes according to residues at the preS1 positions (17, 35, and 51) that are associated with NTCP S267F. Figures 3E and 3F show the fraction of samples for which each haplotype is present (exceeding intra-host frequency of 15%) within genotype C- and B-infected individuals, respectively. We identified putative escape haplotypes that are enriched in heterozygous NTCP S267F (rs2296651-G/A) carriers compared to homozygous WT (rs2296651-G/G) carriers (e.g., ARP in genotype C; AKN/ARN in genotype B). Furthermore, we observed that a higher proportion of heterozygous carriers have multiple intra-host preS1 haplotypes compared to homozygous WT carriers, consistent with the presence of intra-host selective pressure. Figure S14 shows that some heterozygous carriers are infected with mixtures of escape haplotypes (e.g., ARP in genotype C; AKN/ARN in genotype B) and population-consensus haplotypes (e.g., SGH/SGQ in genotype C; SKN in genotype B). For some heterozygous carriers, we were also able to establish possible evolutionary trajectories from the population-consensus haplotypes to the escape haplotypes (Figure S15).

Given that the HBV preS1 mutations associated with the human NTCP variant likely reflect viral escape, it is expected that they would be more prevalent in geographical regions where the NTCP variant is also prevalent. According to gnomAD,[66] the minor allele frequency of rs2296651 (NTCP S267F) is approximately 8% in East Asians and virtually absent in all other populations. Corresponding to the prevalence of the human variant, the HBV mutation most strongly associated with NTCP S267F (preS1 S17A) is predominantly found in genotype B and

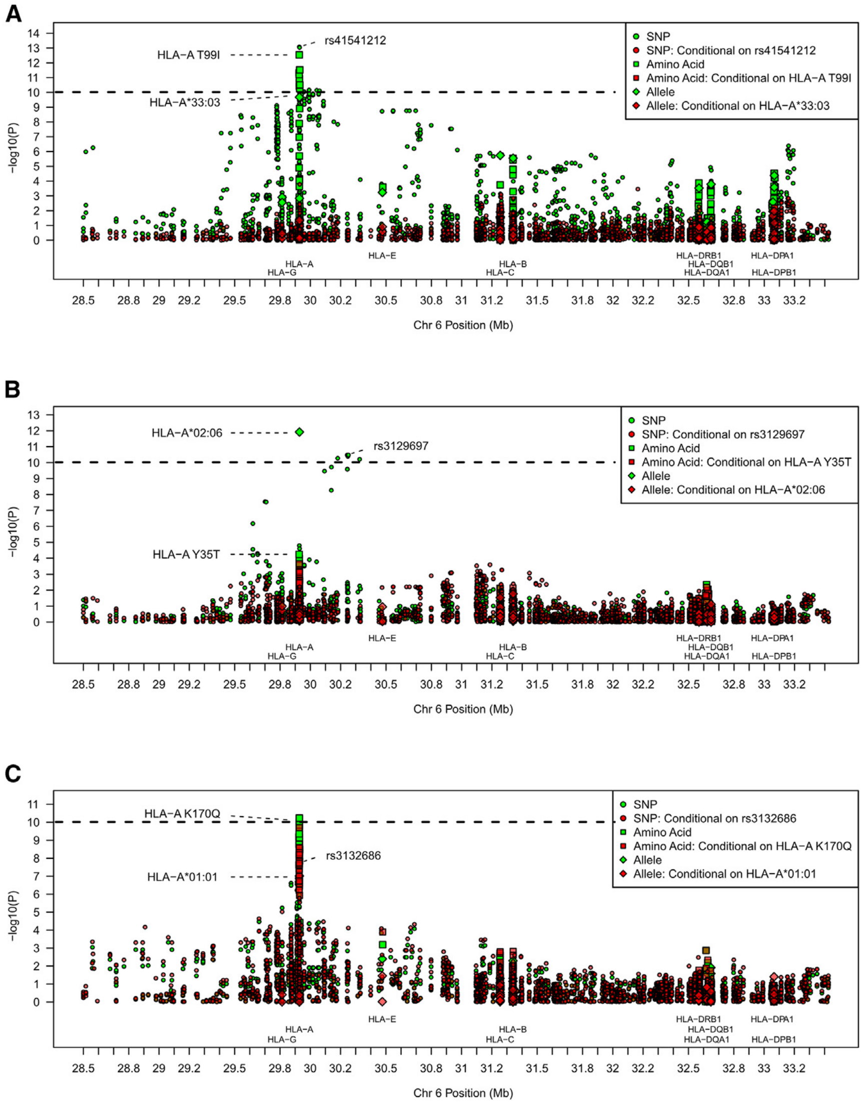

Figure 2. Associations between human variants within the HLA region and HBV amino-acid mutations

Shapes represent the type of human variant (SNP, amino-acid substitution, or HLA allele). Red points represent conditional associations based on the strongest association. Dashed lines represent Bonferroni corrected threshold.

(A) Association with position 160 (associated amino acid: A) of HBV precore/core protein in the East Asian cohort.

(B) Association with position 49 (associated amino acid: N) of HBV polymerase protein in the East Asian cohort.

(C) Association with position 67 (associated amino acid: Y) of HBV precore/core protein in the European cohort.

C HBV samples from East Asian and Southeast Asian countries according to HBVdb[48] (Table S7).

Finally, we evaluated the effect of the NTCP variant and associated preS1 escape haplotypes on biomarkers of HBV infection. We stratified patients according to rs2296651 genotypes and the presence or absence of escape haplotypes (Table 3). Compared to the NTCP WT group, the NTCP S267F non-escape group had lower levels of serum ALT levels ($\beta = -0.22$, $p = 4.4 \times 10^{-4}$) but no significant differences in HBsAg levels ($p = 0.55$) and viral load ($p = 0.14$). Compared to either the NTCP S267F non-escape group or the NTCP WT group, the NTCP S267F escape group had no significant differences in any of the biomarkers. However, the statistical power of this comparison is limited, given the small sample size of the NTCP S267F escape group ($N = 18$).

In vitro preS1-NTCP binding assays

To elucidate the effect of identified preS1 mutations on binding to NTCP, we generated HepG2 cell lines stably expressing NTCP WT or NTCP S267F, both with a hemagglutinin (HA) tag at the N terminus (Figure 4A). As expected, taurocholate uptake performed in HepG2 transduced with HA-NTCP WT or HA-NTCP S267F revealed a reduced functionality of the NTCP variant compared to WT NTCP.[68] HepG2 HA-NTCP WT had a comparable level of taurocholate uptake to a well-established HepG2 NTCP clone (A3),[69] indicating that the introduction of an HA tag does not impair NTCP functionality (Figure S16A). To exclude differences in intracellular and membrane expression between HA-NTCP WT and HA-NTCP S267F, we performed immunofluorescence staining using HA tag antibody and quantification of NTCP mRNA expression via RT-qPCR. Both methodologies revealed a comparable surface and intracellular expression level between NTCP WT and NTCP S267F (Figures S16B and S16C). We then synthesized myristoylated peptides derived from the most common genotype C preS1 haplotype found in NTCP WT carriers (referred to as Myr-WT) and in NTCP S267F carriers (referred to as Myr-ARP, carrying preS1 mutations S17A, G35R, H51P) (Figure 4B). We first assessed their binding capacity to NTCP WT, without (HepG2 NTCP A3 clone), and with HA-tag (HepG2 HA-NTCP WT), indicating no binding impairment when HA is present (Figure S16D). We then performed binding assays to measure their binding affinities to both HA-NTCP WT or HA-NTCP S267F. Myr-ARP and Myr-WT were able to bind NTCP WT with similar affinity (Figures 4C and 4D). Compared to the diminished binding capacity observed between Myr-WT and NTCP S267F, only a partial increase in binding affinity was observed between Myr-ARP and NTCP S267F (Figures 4C and 4D).

Table 3. Impact of rs2296651 and associated preS1 haplotypes on biomarkers of HBV infection

	NTCP WT ($N = 343$) rs2296651:G/G preS1: non-escape	NTCP S267F non-escape ($N = 23$) rs2296651: G/A preS1: non-escape			NTCP S267F escape ($N = 18$) rs2296651: G/A preS1:escape				
			vs. NTCP WT			vs. NTCP S267F non-escape		vs. NTCP WT	
Biomarkers	Mean (SD)	Mean (SD)	Beta (SE)	p	Mean (SD)	Beta (SE)	p	Beta (SE)	p
Viral load ($\log_{10}$ U/L)	6.75 (1.63)	6.82 (1.19)	−0.32 (0.21)	0.14	6.74 (1.17)	−0.06 (0.29)	0.83	−0.35 (0.24)	0.15
Serum ALT ($\log_{10}$ IU/ml)	1.88 (0.31)	1.74 (0.20)	−0.22 (0.06)	4.4e−4	1.78 (0.22)	0.15 (0.06)	0.02	−0.13 (0.07)	0.08
HBsAg level ($\log_{10}$ IU/ml)	3.65 (0.80)	3.63 (0.63)	−0.08 (0.14)	0.55	3.39 (0.46)	−0.30 (0.22)	0.18	−0.33 (0.16)	0.04

The homozygous NTCP WT group represents individuals who carry rs2296651-G/G and are infected with non-escape haplotypes. The heterozygous NTCP S267F non-escape group represents individuals who carry rs2296651-G/A and are infected with non-escape haplotypes. The heterozygous NTCP S267F escape group represents individuals who carry rs2296651-G/A and are infected with escape haplotypes. Escape haplotypes were defined as HBV preS1 haplotypes that are absent in all rs2296651-G/G individuals but present in any rs2296651-G/A individuals. *p* values and beta obtained from multiple linear regression adjusted for age, sex, HBeAg status, the top four human PCs, and the top six pathogen pPCs, and HBV nucleotide mutations significantly associated with the respective biomarker (Figure S10).

HLA class I variations and HBV epitopes

We hypothesized that the associations observed between HLA class I variation and HBV amino-acid mutations might be driven by immune evasion. An individual's HLA class I alleles determine the repertoire of viral epitopes that can be recognized and restricted by CTLs. Depending on the HLA alleles that an individual carries, a selective advantage could be present for specific HBV mutations that alter epitope sequences and lower HLA-binding affinities.

To test this hypothesis, we first identified whether HBV amino-acid mutations associated with HLA-A variation overlap with predicted viral epitopes. We used a sliding window approach to generate binding predictions of the HLA allele against all possible 9- to 14-mer peptides that overlap with the position based on two HLA-binding prediction algorithms (NetMHCpan[63] and MixMHCpred[64]). We observed that all three HLA-associated HBV positions are located within at least one epitope predicted to be strongly bound (elution percentile rank <0.5% and half maximal inhibitory concentration [IC50] < 50 nM) by the respective HLA allele (Table S8).

Next, we evaluated the impact of each HBV mutation on the predicted binding affinities to HLA alleles. Figure 5 shows, for each HLA-associated HBV amino-acid position, the impact of each residue (annotated in bold within each epitope sequence) on predicted binding affinities and their respective frequency in carriers of the corresponding HLA-A allele. For all three HLA alleles, we observed that mutations that result in weaker binding affinities are observed at higher frequencies in carriers of the cognate HLA allele compared to other alleles. Conversely, the population reference residue tends to be conserved if the impact of the mutations on binding affinity is minimal. For example, Figure 5A shows that the substitution of tyrosine at position 67 of precore/core with phenylalanine (LLDTASAL**[Y > F]** in the predicted epitope for the associated allele HLA-A*01:01) results in lower binding affinity. Phenylalanine is observed at higher frequencies in carriers of HLA-A*01:01 compared to carriers of other prevalent HLA-A alleles (Figure S17A). For the other prevalent HLA-A alleles, no strong binding epitope overlaps with the position, and hence, the population consensus residue, tyrosine, is largely conserved. A similar observation was made for the two other HLA alleles and the corresponding HBV epitope mutations (Figures 5B, 5C, S17B, and S17C).

Discussion

By systematically testing for associations between human and HBV genetic variations, we have identified signatures on the HBV genome that reflect selective pressure driven by human genetic variants. We identified associations between an NTCP variant (rs2296651; NTCP S267F) and HBV preS1 mutations, which might reflect escape from the inhibitory effect on viral entry induced by NTCP S267F. We also identified significant associations between HLA-A variation and HBV mutations that overlap with epitopes, pinpointing escape mutations that allow viral evasion from HLA-A presentation and CTL recognition.

A myristoylated lipopeptide that mimics the preS1 receptor-binding region (Bulevirtide, also known as Myrcludex B) can bind to NTCP to block viral entry.[69–71] Using such preS1-derived peptides, a previous study was able to fine-map preS1 residues that are essential for binding to NTCP. The study revealed that two sets of preS1 residues 16–20 (27–31 in our genotype C-based sequence) and 34–48 (45–59 in our genotype C-based sequence) impacted binding, while residues in between them did not.[72] This is consistent with the Alpha-Fold model we constructed (Figure S2), where the first set of preS1 residues is located within the NTCP binding tunnel and the second set of residues is in proximity to patch 1 of NTCP, with residues in between forming a "loop" that is not in contact with NTCP.

Regarding the human NTCP variant (rs2296651, NTCP S267F), *in vitro* studies have demonstrated that the variant has an inhibitory effect on viral entry. Cell line-based

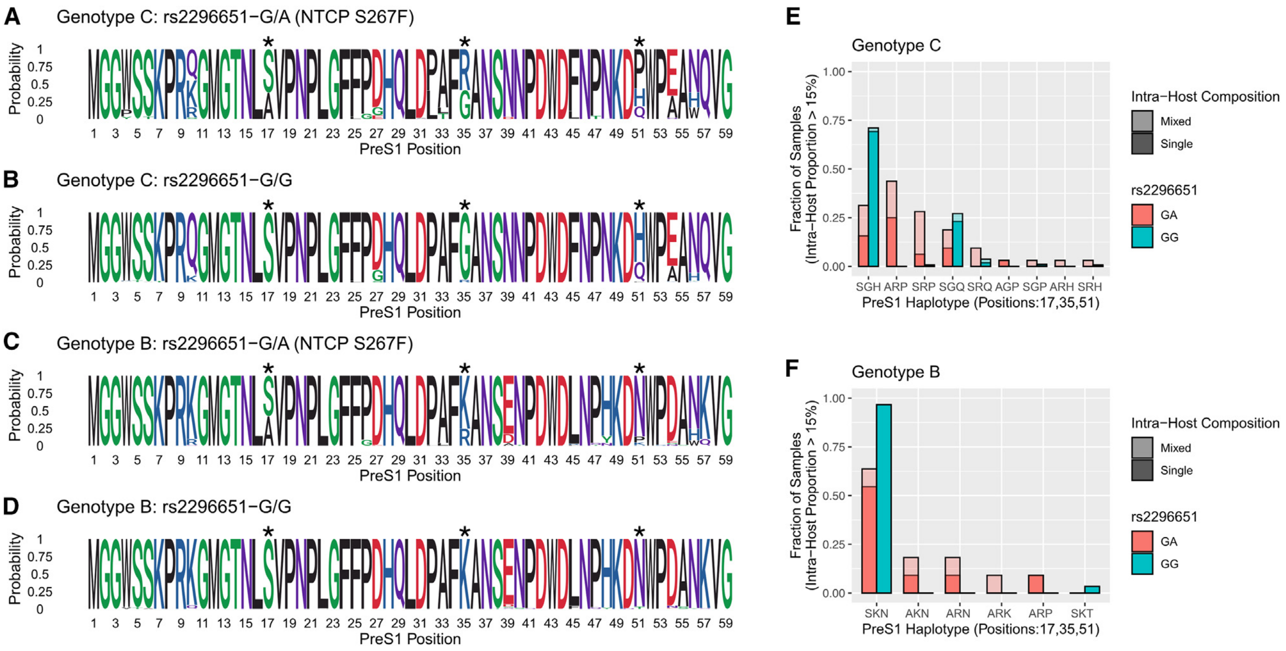

Figure 3. Intra-host composition of preS1 receptor-binding region haplotypes
Sequence logos represent the overall intra-host composition of preS1 receptor-binding region sites. PreS1 sites that show significant association with rs2296651 (NTCP S267F) are marked with asterisks. Bar plots represent the fraction of samples where the haplotype (defined based on the 17th, 35th, and 51st residue of preS1) exceeded the minimal intra-host frequency of 15%. Colors represent rs2296651 genotype. Samples with multiple intra-host haplotypes are considered as mixed.
(A and B) Sequence logo of genotype C-infected individuals who carry rs2296651-G/A and rs2296651-G/G, respectively.
(C and D) Sequence logo of genotype B-infected individuals who carry rs2296651-G/A and rs2296651-G/G, respectively.
(E and F) Frequency of intra-host haplotypes in genotype C- and genotype B-infected individuals, respectively.

studies suggest that preS1-derived peptides cannot bind to NTCP S267F homozygous clones,[68,73] and consequently, HBV infection could not be supported.[68,74] In NTCP S267F/WT heterozygous clones, HBV infection could be supported,[74] albeit potentially less efficiently.[68] Genetic studies in Asian populations support such a protective effect, where heterozygous carriers of the variant have decreased susceptibility to HBV,[12–19] a higher likelihood of spontaneous clearance,[11] and a decreased risk of developing cirrhosis[13,15,19] or HCC[12,14,17] in individuals with chronic hepatitis B. Homozygous carriers of the variant could still be infected with HBV,[15,19] albeit rarely. Limited data suggest that they're asymptomatic if infected.[13] Interestingly, viral escape mutations might have enabled infection of homozygous carriers. A study has pinpointed viral escape mutations in an individual with chronic hepatitis B who is a homozygous carrier of NTCP S267F and infected with HBV genotype B.[75] The identified mutations include the preS1 S17A mutation, which we have found in this study to be associated with NTCP S267F for both genotype C and B.

The positive association that we identified between the NTCP S267F variant and three preS1 residues (17A, 35R, 51P), along with evidence of intra-host selection, suggests that the escape mutations may confer a fitness advantage for HBV within carriers of NTCP S267F. However, results from our peptide binding assays suggest that, compared to preS1 peptides derived from WT genotype C sequences (Myr-WT), peptides that carry the mutations (Myr-ARP) only displayed a partial increase in binding affinity to NTCP S267F. A reason that only a small difference in binding affinity was detected could be that the preS1 mutations might also play a role in downstream processes that are essential for preS1-NTCP internalization. For example, it has been shown that the presence of preS1 is required for NTCP oligomerization and that NTCP oligomerization is essential for preS1-NTCP internalization.[76,77] Furthermore, the NTCP S267F variant has been shown to reduce NTCP oligomerization.[78] Thus, an explanation could be that the preS1 escape mutations also improve the oligomerization efficiency of NTCP S267F-WT heterodimers or NTCP S267F homodimers. Further studies are needed to uncover the exact mechanism.

Although our study supports the protective role of NTCP S267F in individuals with chronic hepatitis B, reflected by significantly reduced serum ALT levels, we only observed a weak effect of HBV escape haplotypes on normalizing serum ALT levels. A likely explanation is that the biomarkers do not directly reflect the efficiency of viral entry and are influenced by multiple mechanisms, which may obscure the direct effects. Additionally, due to the overlapping reading frames of HBV, preS1 mutations associated with NTCP S267F also encode non-synonymous substitutions on the polymerase protein. It is thus possible that the impact on polymerase function may have counterbalanced any functional differences resulting from more

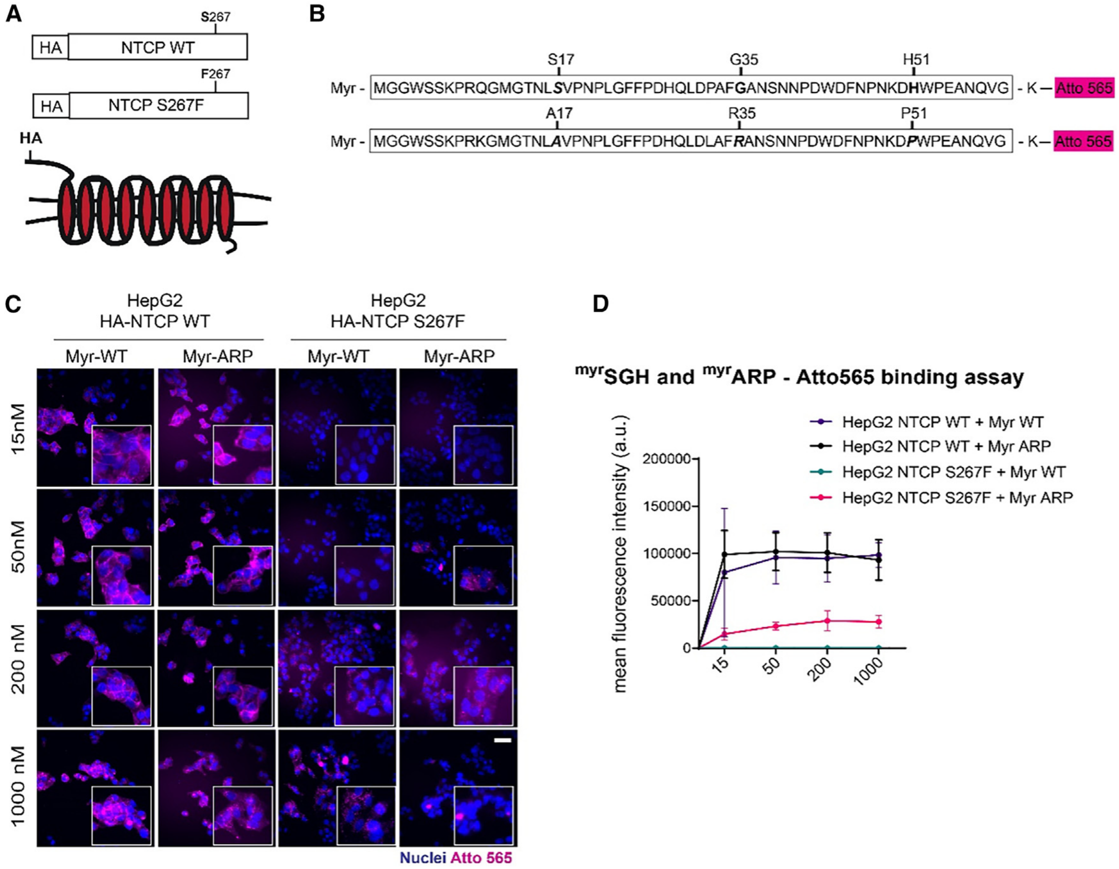

Figure 4. Synthesis of myristoylated peptides for affinity-binding studies in HepG2 HA-NTCP WT and HA-NTCP S267F cells
(A) HepG2 cells were transduced with lentivirus for the stable expression of NTCP WT or NTCP S267F with an HA tag at the N terminus.
(B) Schematic representation of WT and ARP myristoylated peptides synthesized and linked to Atto565-maleimide via addition of a lysine (K) at the C terminus.
(C and D) For binding affinity assays HepG2, HepG2 HA-NTCP WT, and HepG2 HA-NTCP S267F, cells were incubated at increasing concentration (15, 50, 200, or 1,000 nM) of Myr-WT and Myr-ARP peptides. Atto565 signal was detected by fluorescence microscopy (scale bar: 100 μm), (D) or mean fluorescence intensity was measured by flow cytometry and plotted against the respective peptide concentration. Data were normalized by subtracting the autofluorescence at the respective concentration given by the binding in HepG2 cells.

efficient viral entry. Finally, the NTCP S267F variant is also associated with decreased bile acid (taurocholate) uptake by hepatocytes.[79] This has been shown to result in increased serum bile acids levels, based on case reports of homozygous NTCP S267F carriers who are otherwise relatively healthy[80,81] and on a GWAS.[82] Because over-accumulation of bile acids in hepatocytes is thought to lead to liver injury,[83] the variant may indirectly affect the clinical course of hepatitis B through its effect on liver inflammation.

Our findings also have implications with regards to treatment strategies that should be applied to NTCP S267F carriers. Given that entry inhibitors of NTCP such as Bulevirtide have been designed based on WT HBV sequences, their efficacy in NTCP S267F carriers are expected to be lower. As such, further clinical trials could investigate the implications and most optimal dosing regimens in NTCP S267F carriers.

The associations between HBV mutations and HLA-A variation suggest viral escape from human CTL recognition. This is supported by our observation that mutations within epitopes that lead to lower predicted binding affinity are enriched in carriers of their cognate HLA-A alleles. As such, our findings provide direct genetic evidence of HBV escape from HLA recognition, previously shown in other viruses such as HIV-1[34,84–86] and HCV.[33,87–90]

A limitation of our study is that intra-host and inter-host selection processes, which occur during the infection and transmission process, respectively, were not explicitly differentiated. For example, G2G associations could be driven by differences in susceptibility to infection or development of chronicity that is dependent on both host and pathogen genetic factors. Alternatively, G2G associations could be the result of convergent intra-host selection of mutants, illustrating the emergence of escape mutations and adaptation of viruses to the host genetic background during the course of infection. Owning to the relatively high mutation rate of HBV compared to other DNA viruses,[26] intra-host selection has been demonstrated to be a valid mechanism during chronic HBV infection.[91] Past studies in fast-evolving viruses such as HIV[21,84] lead us to

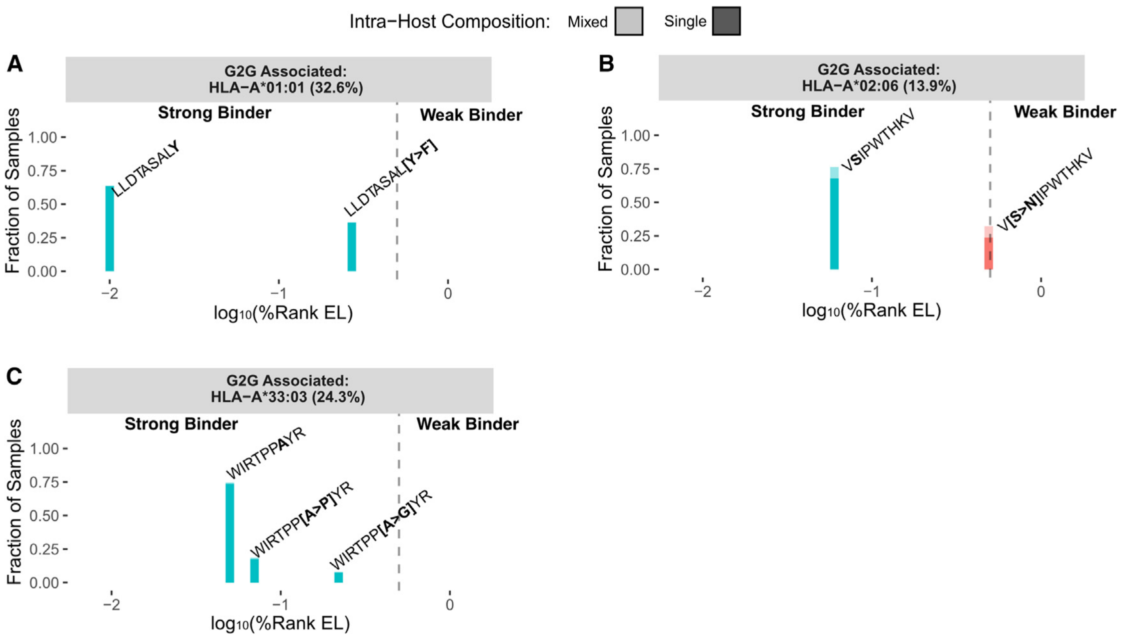

Figure 5. Impact of HBV amino-acid mutations on binding affinity, focusing on viral positions that are associated with HLA-A alleles
Each panel refers to the binding affinities of peptides to the associated HLA-A allele, with the frequency of the HLA allele within the cohort indicated in parentheses. Peptide amino-acid sequence is shown above each bar and the relative position of the HLA-associated HBV position is indicated in bold. x axis represents the binding affinity of each peptide, and y axis represents the fraction of carriers of the specified HLA-A allele for which the peptide is present in the viral sample. Shading represents whether the peptide is present as part of a mixture in the intra-host level (multiple peptides in the intra-host viral population) or not. The dotted lines indicate the threshold of a strong binder, defined as elution percentile rank less than 0.5%.
(A) Referring to the negative association between HLA-A*01:01 and position 67 (associated amino acid: Y) of HBV precore/core protein in the European cohort.
(B) Referring to the positive association between HLA-A*02:06 and position 49 (associated amino acid: N) of HBV polymerase protein in the East Asian cohort.
(C) Referring to the negative association between HLA-A*33:03 and position 160 (associated amino acid: A) of HBV precore/core protein in the East Asian cohort.

believe that HLA-induced viral escape mutations that we observed in this study might be primarily driven by intra-host selective forces. On the other hand, the characteristics of the selective mechanism responsible for associations between NTCP S267F and HBV preS1 mutations are less clear. Our observation of increased intra-host positive selection within the preS1 receptor-binding region in rs2296651-G/A individuals and examples of valid evolutionary trajectories toward escape haplotypes support the presence of intra-host selective pressure. However, given that information regarding length of infection was not available as part of this study, further longitudinal studies with paired HBV and human genomic data would be required to validate our interpretations and disentangle these two levels of selective forces more directly.

Another limitation of our study is that the overall sample size is modest and primarily composed of individuals with East Asian ancestry. Our findings regarding NTCP were specific to the East Asian cohort. One explanation could be that the burden of HBV infection in East Asia has imposed stronger positive selection pressure on humans compared to Europe, hence increasing the frequency of protective NTCP variants. Another explanation could be the limited statistical power within the European cohort due to the small sample size. Polymorphisms in *NTCP* that impact function have also been identified in European and African populations.[79] Thus, further studies with paired human and HBV genomic data in other populations, such as African populations where the burden of HBV infection is also among the highest, would strengthen the findings reported here.

A final limitation of our study is that human exome rather than whole-genome sequencing data were utilized. This means that there might be potential associations within non-exonic regions of the genome that this study missed. For example, it remains unclear whether genetic variants within the regulatory regions of *SLC10A1* could also induce evolutionary changes in HBV.

Overall, our results are consistent with previous studies that have established human NTCP and HLA genetic variations as determinants of hepatitis-related outcomes but provide additional genetic evidence that they could also drive HBV evolutionary changes. We also illustrate the potential of the G2G approach as an agnostic and hypothesis-free method to identify mechanisms involved in host-pathogen interactions, revealing insights that can be relevant for treatment and vaccine development.

Data and code availability

Summary statistics are available on Zenodo (https://doi.org/10.5281/zenodo.8279455). Software code is available on GitHub (https://github.com/zmx21/G2G-HBV-Snakemake). Viral sequencing data have been deposited on the European Genome-Phenome Archive (ega-archive.org) under accession EGA: EGAS00001003689. Human exome sequencing data are not available due to privacy concerns.

Supplemental information

Supplemental information can be found online at https://doi.org/10.1016/j.ajhg.2024.04.013.

Acknowledgments

This work was supported by the Swiss National Science Foundation (grant #197721 to J.F.), by Gilead Sciences (unrestricted research grant to J.F.), and by EPFL internal funding. G.E.G. and S.U. were funded by German Center for Infectious Research (DZIF), TTU Hepatitis Projects 5.709 and 5.822, and by the Deutsche Forschungsgemeinschaft (DFG, German Research Foundation) Projektnummer 272983813 – TRR 179 (TP15).

We would like to thank Biao Li, Christian Thorball, and Nimisha Chaturvedi for bioinformatics support.

Author contributions

Conceptualization: Z.M.X., G.E.G., S.R., D.G., S.U., J.F.; methodology: Z.M.X., G.E.G., S.R., P.R.S., D.L., O.N., A. Scheck, A. Schneuing, T.J., D.M., O.P., D.G., S.U., J.F.; formal analysis: Z.M.X., G.E.G., S.R., A. Scheck, A. Schneuing, D.G.; software: Z.M.X., S.R., M.Z.; investigation: Z.M.X., G.E.G., S.R.; visualization: Z.M.X., G.E.G., S.R.; supervision: B.C., V.S., S.U., J.F.; project administration: M.B., H.L.Y.C., P.M., O.P., V.S., A.G., M.S., S.U., J.F.; funding acquisition: J.F.; writing – original draft: Z.M.X., G.E.G., J.F.; writing – review & editing: all authors.

Declaration of interests

O.P, A.G., M.S., and V.S. are employees of Gilead Sciences Inc. O.N. is now an employee of SUN bioscience SA. S.R. is now an employee of Novartis AG. A. Scheck is now an employee of RIDGELINE Discovery GmbH. M.B. has received research funding from AB Altoimmune, Janssen, GSK, Gilead, and AbbVie. H.L.Y.C. is an advisor for Aligos, Arbutus, Gilead Sciences, Glaxo-Smith-Kline, Roche, Vaccitech, Vir Biotechnology, and Virion Therapeutics and a speaker for Echosens, Gilead Sciences, Roche, Mylan.

Received: January 8, 2024
Accepted: April 18, 2024
Published: May 14, 2024

References

1. World Health Organization (2021). Global Progress Report on HIV, Viral Hepatitis and Sexually Transmitted Infections, 2021: Accountability for the Global Health Sector Strategies 2016–2021: Actions for Impact (World Health Organization).
2. Ganem, D., and Prince, A.M. (2004). Hepatitis B Virus Infection — Natural History and Clinical Consequences. N. Engl. J. Med. *350*, 1118–1129. https://doi.org/10.1056/NEJMra031087.
3. Zhang, Z., Wang, C., Liu, Z., Zou, G., Li, J., and Lu, M. (2019). Host Genetic Determinants of Hepatitis B Virus Infection. Front. Genet. *10*, 696. https://doi.org/10.3389/fgene.2019.00696.
4. Seshasubramanian, V., Soundararajan, G., and Ramasamy, P. (2018). Human leukocyte antigen A, B and Hepatitis B infection outcome: A meta-analysis. Infect. Genet. Evol. *66*, 392–398. https://doi.org/10.1016/j.meegid.2017.07.027.
5. Kamatani, Y., Wattanapokayakit, S., Ochi, H., Kawaguchi, T., Takahashi, A., Hosono, N., Kubo, M., Tsunoda, T., Kamatani, N., Kumada, H., et al. (2009). A genome-wide association study identifies variants in the HLA-DP locus associated with chronic hepatitis B in Asians. Nat. Genet. *41*, 591–595. https://doi.org/10.1038/ng.348.
6. Cheong, J.Y., Cho, S.W., Hwang, I.L., Yoon, S.K., Lee, J.H., Park, C.S., Lee, J.E., Hahm, K.B., and Kim, J.H. (2006). Association between chronic hepatitis B virus infection and interleukin-10, tumor necrosis factor-alpha gene promoter polymorphisms. J. Gastroenterol. Hepatol. *21*, 1163–1169. https://doi.org/10.1111/j.1440-1746.2006.04304.x.
7. Karra, V.K., Gumma, P.K., Chowdhury, S.J., Ruttala, R., Polipalli, S.K., Chakravarti, A., and Kar, P. (2015). IL-18 polymorphisms in hepatitis B virus related liver disease. Cytokine *73*, 277–282. https://doi.org/10.1016/j.cyto.2015.02.015.
8. Tunçbilek, S. (2014). Relationship between cytokine gene polymorphisms and chronic hepatitis B virus infection. World J. Gastroenterol. *20*, 6226–6235. https://doi.org/10.3748/wjg.v20.i20.6226.
9. Huang, X., Li, H., Wang, J., Huang, C., Lu, Y., Qin, X., and Li, S. (2015). Genetic polymorphisms in Toll-like receptor 3 gene are associated with the risk of hepatitis B virus-related liver diseases in a Chinese population. Gene *569*, 218–224. https://doi.org/10.1016/j.gene.2015.05.054.
10. Zhu, J., Zhang, T., Cao, L., Li, A., Zheng, K., Zhang, N., Su, B., Chen, Z., Chen, N., Wu, H., and He, Q. (2017). Toll like receptor7 polymorphisms in relation to disease susceptibility and progression in Chinese patients with chronic HBV infection. Sci. Rep. *7*, 12417. https://doi.org/10.1038/s41598-017-12698-5.
11. Hu, P., Liu, J., and Zhang, D. (2019). Association of NTCP Gene Polymorphisms and Spontaneous Clearance of Hepatitis B Virus in Asia: A Meta-Analysis. Hepat. Mon. *19*. https://doi.org/10.5812/hepatmon.94500.
12. Wang, P., Mo, R., Lai, R., Xu, Y., Lu, J., Zhao, G., Liu, Y., Cao, Z., Wang, X., Li, Z., et al. (2017). Genetic variations of NTCP are associated with susceptibility to HBV infection and related hepatocellular carcinoma. Oncotarget *8*, 105407–105424. https://doi.org/10.18632/oncotarget.22211.
13. An, P., Zeng, Z., and Winkler, C.A. (2018). The Loss-of-Function S267F Variant in HBV Receptor NTCP Reduces Human Risk for HBV Infection and Disease Progression. J. Infect. Dis. *218*, 1404–1410. https://doi.org/10.1093/infdis/jiy355.
14. Binh, M.T., Hoan, N.X., Van Tong, H., Sy, B.T., Trung, N.T., Bock, C.-T., Toan, N.L., Song, L.H., Bang, M.H., Meyer, C.G., et al. (2019). NTCP S267F variant associates with decreased susceptibility to HBV and HDV infection and decelerated progression of related liver diseases. Int. J. Infect. Dis. *80*, 147–152. https://doi.org/10.1016/j.ijid.2019.01.038.

15. Lee, H.W., Park, H.J., Jin, B., Dezhbord, M., Kim, D.Y., Han, K.-H., Ryu, W.-S., Kim, S., and Ahn, S.H. (2017). Effect of S267F variant of NTCP on the patients with chronic hepatitis B. Sci. Rep. *7*, 17634. https://doi.org/10.1038/s41598-017-17959-x.
16. Wu, W., Zeng, Y., Lin, J., Wu, Y., Chen, T., Xun, Z., and Ou, Q. (2018). Genetic variants in *NTCP* exon gene are associated with HBV infection status in a Chinese Han population: *NTCP* variants associated with HBV infection status. Hepatol. Res. *48*, 364–372. https://doi.org/10.1111/hepr.13007.
17. Chuaypen, N., Tuyapala, N., Pinjaroen, N., Payungporn, S., and Tangkijvanich, P. (2019). Association of NTCP polymorphisms with clinical outcome of hepatitis B infection in Thai individuals. BMC Med. Genet. *20*, 87. https://doi.org/10.1186/s12881-019-0823-x.
18. Li, N., Zhang, P., Yang, C., Zhu, Q., Li, Z., Li, F., Han, Q., Wang, Y., Lv, Y., Wei, P., and Liu, Z. (2014). Association of Genetic Variation of Sodium Taurocholate Cotransporting Polypeptide with Chronic Hepatitis B Virus Infection. Genet. Test. Mol. Biomarkers *18*, 425–429. https://doi.org/10.1089/gtmb.2013.0491.
19. Peng, L., Zhao, Q., Li, Q., Li, M., Li, C., Xu, T., Jing, X., Zhu, X., Wang, Y., Li, F., et al. (2015). The p.Ser267Phe variant in *SLC10A1* is associated with resistance to chronic hepatitis B: Viral Hepatitis. Hepatology *61*, 1251–1260. https://doi.org/10.1002/hep.27608.
20. Daugherty, M.D., and Malik, H.S. (2012). Rules of Engagement: Molecular Insights from Host-Virus Arms Races. Annu. Rev. Genet. *46*, 677–700. https://doi.org/10.1146/annurev-genet-110711-155522.
21. Fryer, H.R., Frater, J., Duda, A., Roberts, M.G., SPARTAC Trial Investigators, Phillips, R.E., and McLean, A.R. (2010). Modelling the Evolution and Spread of HIV Immune Escape Mutants. PLoS Pathog. *6*, e1001196. https://doi.org/10.1371/journal.ppat.1001196.
22. Kelleher, A.D., Long, C., Holmes, E.C., Allen, R.L., Wilson, J., Conlon, C., Workman, C., Shaunak, S., Olson, K., Goulder, P., et al. (2001). Clustered Mutations in HIV-1 Gag Are Consistently Required for Escape from Hla-B27–Restricted Cytotoxic T Lymphocyte Responses. J. Exp. Med. *193*, 375–386. https://doi.org/10.1084/jem.193.3.375.
23. Price, D.A., Goulder, P.J., Klenerman, P., Sewell, A.K., Easterbrook, P.J., Troop, M., Bangham, C.R., and Phillips, R.E. (1997). Positive selection of HIV-1 cytotoxic T lymphocyte escape variants during primary infection. Proc. Natl. Acad. Sci. USA *94*, 1890–1895. https://doi.org/10.1073/pnas.94.5.1890.
24. Geels, M.J., Cornelissen, M., Schuitemaker, H., Anderson, K., Kwa, D., Maas, J., Dekker, J.T., Baan, E., Zorgdrager, F., van den Burg, R., et al. (2003). Identification of Sequential Viral Escape Mutants Associated with Altered T-Cell Responses in a Human Immunodeficiency Virus Type 1-Infected Individual. JVI *77*, 12430–12440. https://doi.org/10.1128/JVI.77.23.12430-12440.2003.
25. Phillips, R.E., Rowland-Jones, S., Nixon, D.F., Gotch, F.M., Edwards, J.P., Ogunlesi, A.O., Elvin, J.G., Rothbard, J.A., Bangham, C.R., Rizza, C.R., et al. (1991). Human immunodeficiency virus genetic variation that can escape cytotoxic T cell recognition. Nature *354*, 453–459. https://doi.org/10.1038/354453a0.
26. Lin, Y.-Y., Liu, C., Chien, W.-H., Wu, L.-L., Tao, Y., Wu, D., Lu, X., Hsieh, C.-H., Chen, P.-J., Wang, H.-Y., et al. (2015). New Insights into the Evolutionary Rate of Hepatitis B Virus at Different Biological Scales. J. Virol. *89*, 3512–3522. https://doi.org/10.1128/JVI.03131-14.
27. Zhang, A.-Y., Lai, C.-L., Huang, F.-Y., Seto, W.-K., Fung, J., Wong, D.K.-H., and Yuen, M.-F. (2017). Deep sequencing analysis of quasispecies in the HBV pre-S region and its association with hepatocellular carcinoma. J. Gastroenterol. *52*, 1064–1074. https://doi.org/10.1007/s00535-017-1334-1.
28. Lumley, S.F., McNaughton, A.L., Klenerman, P., Lythgoe, K.A., and Matthews, P.C. (2018). Hepatitis B Virus Adaptation to the CD8+ T Cell Response: Consequences for Host and Pathogen. Front. Immunol. *9*, 1561. https://doi.org/10.3389/fimmu.2018.01561.
29. Kefalakes, H., Budeus, B., Walker, A., Jochum, C., Hilgard, G., Heinold, A., Heinemann, F.M., Gerken, G., Hoffmann, D., and Timm, J. (2015). Adaptation of the hepatitis B virus core protein to CD8 $^{+}$ T-cell selection pressure: VIRAL HEPATITIS. Hepatology *62*, 47–56. https://doi.org/10.1002/hep.27771.
30. Abbott, W.G.H., Tsai, P., Leung, E., Trevarton, A., Ofanoa, M., Hornell, J., Gane, E.J., Munn, S.R., and Rodrigo, A.G. (2010). Associations between HLA Class I Alleles and Escape Mutations in the Hepatitis B Virus Core Gene in New Zealand-Resident Tongans. JVI *84*, 621–629. https://doi.org/10.1128/JVI.01471-09.
31. Khakoo, S.I., Ling, R., Scott, I., Dodi, A.I., Harrison, T.J., Dusheiko, G.M., and Madrigal, J.A. (2000). Cytotoxic T lymphocyte responses and CTL epitope escape mutation in HBsAg, anti-HBe positive individuals. Gut *47*, 137–143. https://doi.org/10.1136/gut.47.1.137.
32. Fellay, J., and Pedergnana, V. (2020). Exploring the interactions between the human and viral genomes. Hum. Genet. *139*, 777–781. https://doi.org/10.1007/s00439-019-02089-3.
33. STOP-HCV Consortium, Ansari, M.A., Pedergnana, V., L C Ip, C., Magri, A., Von Delft, A., Bonsall, D., Chaturvedi, N., Bartha, I., Smith, D., Nicholson, G., et al. (2017). Genome-to-genome analysis highlights the effect of the human innate and adaptive immune systems on the hepatitis C virus. Nat. Genet. *49*, 666–673. https://doi.org/10.1038/ng.3835.
34. Bartha, I., Carlson, J.M., Brumme, C.J., McLaren, P.J., Brumme, Z.L., John, M., Haas, D.W., Martinez-Picado, J., Dalmau, J., López-Galíndez, C., et al. (2013). A genome-to-genome analysis of associations between human genetic variation, HIV-1 sequence diversity, and viral control. Elife *2*, e01123. https://doi.org/10.7554/eLife.01123.
35. Agarwal, K., Ahn, S.H., Elkhashab, M., Lau, A.H., Gaggar, A., Bulusu, A., Tian, X., Cathcart, A.L., Woo, J., Subramanian, G.M., et al. (2018). Safety and efficacy of vesatolimod (GS-9620) in patients with chronic hepatitis B who are not currently on antiviral treatment. J. Viral Hepat. *25*, 1331–1340. https://doi.org/10.1111/jvh.12942.
36. Buti, M., Gane, E., Seto, W.K., Chan, H.L.Y., Chuang, W.-L., Stepanova, T., Hui, A.-J., Lim, Y.-S., Mehta, R., Janssen, H.L.A., et al. (2016). Tenofovir alafenamide versus tenofovir disoproxil fumarate for the treatment of patients with HBeAg-negative chronic hepatitis B virus infection: a randomised, double-blind, phase 3, non-inferiority trial. Lancet. Gastroenterol. Hepatol. *1*, 196–206. https://doi.org/10.1016/S2468-1253(16)30107-8.
37. Chan, H.L.Y., Fung, S., Seto, W.K., Chuang, W.-L., Chen, C.-Y., Kim, H.J., Hui, A.J., Janssen, H.L.A., Chowdhury, A., Tsang, T.Y.O., et al. (2016). Tenofovir alafenamide versus tenofovir disoproxil fumarate for the treatment of HBeAg-positive chronic

hepatitis B virus infection: a randomised, double-blind, phase 3, non-inferiority trial. Lancet. Gastroenterol. Hepatol. *1*, 185–195. https://doi.org/10.1016/S2468-1253(16)30024-3.

38. Boni, C., Janssen, H.L.A., Rossi, M., Yoon, S.K., Vecchi, A., Barili, V., Yoshida, E.M., Trinh, H., Rodell, T.C., Laccabue, D., et al. (2019). Combined GS-4774 and Tenofovir Therapy Can Improve HBV-Specific T-Cell Responses in Patients With Chronic Hepatitis. Gastroenterology *157*, 227–241.e7. https://doi.org/10.1053/j.gastro.2019.03.044.
39. Auwera, G.A., Carneiro, M.O., Hartl, C., Poplin, R., del Angel, G., Levy-Moonshine, A., Jordan, T., Shakir, K., Roazen, D., Thibault, J., et al. (2013). From FastQ Data to High-Confidence Variant Calls: The Genome Analysis Toolkit Best Practices Pipeline. Current Protocols in Bioinformatics *43*. https://doi.org/10.1002/0471250953.bi1110s43.
40. Chang, C.C., Chow, C.C., Tellier, L.C., Vattikuti, S., Purcell, S.M., and Lee, J.J. (2015). Second-generation PLINK: rising to the challenge of larger and richer datasets. GigaScience *4*, 7. https://doi.org/10.1186/s13742-015-0047-8.
41. Manichaikul, A., Mychaleckyj, J.C., Rich, S.S., Daly, K., Sale, M., and Chen, W.-M. (2010). Robust relationship inference in genome-wide association studies. Bioinformatics *26*, 2867–2873. https://doi.org/10.1093/bioinformatics/btq559.
42. 1000 Genomes Project Consortium, Auton, A., Brooks, L.D., Durbin, R.M., Garrison, E.P., Kang, H.M., Korbel, J.O., Marchini, J.L., McCarthy, S., McVean, G.A., and Abecasis, G.R. (2015). A global reference for human genetic variation. Nature *526*, 68–74. https://doi.org/10.1038/nature15393.
43. Jiang, L., Zheng, Z., Qi, T., Kemper, K.E., Wray, N.R., Visscher, P.M., and Yang, J. (2019). A resource-efficient tool for mixed model association analysis of large-scale data. Nat. Genet. *51*, 1749–1755. https://doi.org/10.1038/s41588-019-0530-8.
44. Dilthey, A.T., Mentzer, A.J., Carapito, R., Cutland, C., Cereb, N., Madhi, S.A., Rhie, A., Koren, S., Bahram, S., McVean, G., and Phillippy, A.M. (2019). HLA*LA—HLA typing from linearly projected graph alignments. Bioinformatics *35*, 4394–4396. https://doi.org/10.1093/bioinformatics/btz235.
45. Fan, Y., and Song, Y.-Q. (2017). PyHLA: tests for the association between HLA alleles and diseases. BMC Bioinf. *18*, 90. https://doi.org/10.1186/s12859-017-1496-0.
46. Günther, S., Li, B.C., Miska, S., Krüger, D.H., Meisel, H., and Will, H. (1995). A novel method for efficient amplification of whole hepatitis B virus genomes permits rapid functional analysis and reveals deletion mutants in immunosuppressed patients. J. Virol. *69*, 5437–5444. https://doi.org/10.1128/JVI.69.9.5437-5444.1995.
47. Bolger, A.M., Lohse, M., and Usadel, B. (2014). Trimmomatic: a flexible trimmer for Illumina sequence data. Bioinformatics *30*, 2114–2120. https://doi.org/10.1093/bioinformatics/btu170.
48. Hayer, J., Jadeau, F., Deléage, G., Kay, A., Zoulim, F., and Combet, C. (2013). HBVdb: a knowledge database for Hepatitis B Virus. Nucleic Acids Res. *41*, D566–D570. https://doi.org/10.1093/nar/gks1022.
49. Minh, B.Q., Schmidt, H.A., Chernomor, O., Schrempf, D., Woodhams, M.D., von Haeseler, A., and Lanfear, R. (2020). IQ-TREE 2: New Models and Efficient Methods for Phylogenetic Inference in the Genomic Era. Mol. Biol. Evol. *37*, 1530–1534. https://doi.org/10.1093/molbev/msaa015.
50. Hoang, D.T., Chernomor, O., von Haeseler, A., Minh, B.Q., and Vinh, L.S. (2018). UFBoot2: Improving the Ultrafast Bootstrap Approximation. Mol. Biol. Evol. *35*, 518–522. https://doi.org/10.1093/molbev/msx281.
51. McNaughton, A.L., Revill, P.A., Littlejohn, M., Matthews, P.C., and Ansari, M.A. (2020). Analysis of genomic-length HBV sequences to determine genotype and subgenotype reference sequences. J. Gen. Virol. *101*, 271–283. https://doi.org/10.1099/jgv.0.001387.
52. Jombart, T., Pavoine, S., Devillard, S., and Pontier, D. (2010). Putting phylogeny into the analysis of biological traits: A methodological approach. J. Theor. Biol. *264*, 693–701. https://doi.org/10.1016/j.jtbi.2010.03.038.
53. Zhou, W., Nielsen, J.B., Fritsche, L.G., Dey, R., Gabrielsen, M.E., Wolford, B.N., LeFaive, J., VandeHaar, P., Gagliano, S.A., Gifford, A., et al. (2018). Efficiently controlling for case-control imbalance and sample relatedness in large-scale genetic association studies. Nat. Genet. *50*, 1335–1341. https://doi.org/10.1038/s41588-018-0184-y.
54. Mirdita, M., Schütze, K., Moriwaki, Y., Heo, L., Ovchinnikov, S., and Steinegger, M. (2022). ColabFold: making protein folding accessible to all. Nat. Methods *19*, 679–682. https://doi.org/10.1038/s41592-022-01488-1.
55. Evans, R., O'Neill, M., Pritzel, A., Antropova, N., Senior, A., Green, T., Žídek, A., Bates, R., Blackwell, S., Yim, J., et al. (2022). Protein complex prediction with AlphaFold-Multimer. Preprint at bioRxiv. https://doi.org/10.1101/2021.10.04.463034.
56. Asami, J., Kimura, K.T., Fujita-Fujiharu, Y., Ishida, H., Zhang, Z., Nomura, Y., Liu, K., Uemura, T., Sato, Y., Ono, M., et al. (2022). Structure of the bile acid transporter and HBV receptor NTCP. Nature *606*, 1021–1026. https://doi.org/10.1038/s41586-022-04845-4.
57. Pettersen, E.F., Goddard, T.D., Huang, C.C., Couch, G.S., Greenblatt, D.M., Meng, E.C., and Ferrin, T.E. (2004). UCSF Chimera?A visualization system for exploratory research and analysis. J. Comput. Chem. *25*, 1605–1612. https://doi.org/10.1002/jcc.20084.
58. Zagordi, O., Bhattacharya, A., Eriksson, N., and Beerenwinkel, N. (2011). ShoRAH: estimating the genetic diversity of a mixed sample from next-generation sequencing data. BMC Bioinf. *12*, 119. https://doi.org/10.1186/1471-2105-12-119.
59. Charif, D., and Lobry, J.R. (2007). SeqinR 1.0-2: A Contributed Package to the R Project for Statistical Computing Devoted to Biological Sequences Retrieval and Analysis. In Structural Approaches to Sequence Evolution: Molecules, Networks, Populations, U. Bastolla, M. Porto, H.E. Roman, and M. Vendruscolo, eds. (Springer Berlin Heidelberg), pp. 207–232. https://doi.org/10.1007/978-3-540-35306-5_10.
60. Wagih, O. (2017). ggseqlogo: a versatile R package for drawing sequence logos. Bioinformatics *33*, 3645–3647. https://doi.org/10.1093/bioinformatics/btx469.
61. Nelson, C.W., and Hughes, A.L. (2015). Within-host nucleotide diversity of virus populations: Insights from next-generation sequencing. Infect. Genet. Evol. *30*, 1–7. https://doi.org/10.1016/j.meegid.2014.11.026.
62. Nelson, C.W., Ardern, Z., and Wei, X. (2020). OLGenie: Estimating Natural Selection to Predict Functional Overlapping Genes. Mol. Biol. Evol. *37*, 2440–2449. https://doi.org/10.1093/molbev/msaa087.
63. Jurtz, V., Paul, S., Andreatta, M., Marcatili, P., Peters, B., and Nielsen, M. (2017). NetMHCpan-4.0: Improved Peptide–MHC Class I Interaction Predictions Integrating Eluted Ligand and Peptide Binding Affinity Data. J. ICE *199*, 3360–3368. https://doi.org/10.4049/jimmunol.1700893.
64. Gfeller, D., Schmidt, J., Croce, G., Guillaume, P., Bobisse, S., Genolet, R., Queiroz, L., Cesbron, J., Racle, J., and Harari, A.

(2023). Improved predictions of antigen presentation and TCR recognition with MixMHCpred2.2 and PRIME2.0 reveal potent SARS-CoV-2 CD8+ T-cell epitopes. Cell Syst. *14*, 72–83.e5. https://doi.org/10.1016/j.cels.2022.12.002.

65. Yan, H., Zhong, G., Xu, G., He, W., Jing, Z., Gao, Z., Huang, Y., Qi, Y., Peng, B., Wang, H., et al. (2012). Sodium taurocholate cotransporting polypeptide is a functional receptor for human hepatitis B and D virus. Elife *1*, e00049. https://doi.org/10.7554/eLife.00049.
66. Karczewski, K.J., Francioli, L.C., Tiao, G., Cummings, B.B., Alföldi, J., Alföldi, J., Collins, R.L., Laricchia, K.M., Ganna, A., Birnbaum, D.P., et al. (2020). The mutational constraint spectrum quantified from variation in 141,456 humans. Nature *581*, 434–443. https://doi.org/10.1038/s41586-020-2308-7.
67. Yan, H., Peng, B., He, W., Zhong, G., Qi, Y., Ren, B., Gao, Z., Jing, Z., Song, M., Xu, G., et al. (2013). Molecular Determinants of Hepatitis B and D Virus Entry Restriction in Mouse Sodium Taurocholate Cotransporting Polypeptide. J. Virol. *87*, 7977–7991. https://doi.org/10.1128/JVI.03540-12.
68. Yan, H., Peng, B., Liu, Y., Xu, G., He, W., Ren, B., Jing, Z., Sui, J., and Li, W. (2014). Viral Entry of Hepatitis B and D Viruses and Bile Salts Transportation Share Common Molecular Determinants on Sodium Taurocholate Cotransporting Polypeptide. J. Virol. *88*, 3273–3284. https://doi.org/10.1128/JVI.03478-13.
69. Ni, Y., Lempp, F.A., Mehrle, S., Nkongolo, S., Kaufman, C., Fälth, M., Stindt, J., Königer, C., Nassal, M., Kubitz, R., et al. (2014). Hepatitis B and D Viruses Exploit Sodium Taurocholate Co-transporting Polypeptide for Species-Specific Entry into Hepatocytes. Gastroenterology *146*, 1070–1083. https://doi.org/10.1053/j.gastro.2013.12.024.
70. Glebe, D., Urban, S., Knoop, E.V., Çağ, N., Krass, P., Grün, S., Bulavaite, A., Sasnauskas, K., and Gerlich, W.H. (2005). Mapping of the Hepatitis B Virus Attachment Site by Use of Infection-Inhibiting preS1 Lipopeptides and Tupaia Hepatocytes. Gastroenterology *129*, 234–245. https://doi.org/10.1053/j.gastro.2005.03.090.
71. Volz, T., Allweiss, L., ḾBarek, M.B., Warlich, M., Lohse, A.W., Pollok, J.M., Alexandrov, A., Urban, S., Petersen, J., Lütgehetmann, M., and Dandri, M. (2013). The entry inhibitor Myrcludex-B efficiently blocks intrahepatic virus spreading in humanized mice previously infected with hepatitis B virus. J. Hepatol. *58*, 861–867. https://doi.org/10.1016/j.jhep.2012.12.008.
72. Schulze, A., Schieck, A., Ni, Y., Mier, W., and Urban, S. (2010). Fine Mapping of Pre-S Sequence Requirements for Hepatitis B Virus Large Envelope Protein-Mediated Receptor Interaction. J. Virol. *84*, 1989–2000. https://doi.org/10.1128/JVI.01902-09.
73. Donkers, J.M., Appelman, M.D., and van de Graaf, S.F.J. (2019). Mechanistic insights into the inhibition of NTCP by myrcludex B. JHEP Rep. *1*, 278–285. https://doi.org/10.1016/j.jhepr.2019.07.006.
74. Uchida, T., Park, S.B., Inuzuka, T., Zhang, M., Allen, J.N., Chayama, K., and Liang, T.J. (2021). Genetically edited hepatic cells expressing the NTCP-S267F variant are resistant to hepatitis B virus infection. Mol. Ther. Methods Clin. Dev. *23*, 597–605. https://doi.org/10.1016/j.omtm.2021.11.002.
75. Liu, C., Xu, G., Gao, Z., Zhou, Z., Guo, G., Li, D., Jing, Z., Sui, J., and Li, W. (2018). The p.Ser267Phe variant of sodium taurocholate cotransporting polypeptide (NTCP) supports HBV infection with a low efficiency. Virology *522*, 168–176. https://doi.org/10.1016/j.virol.2018.07.006.
76. Fukano, K., Oshima, M., Tsukuda, S., Aizaki, H., Ohki, M., Park, S.-Y., Wakita, T., Wakae, K., Watashi, K., and Muramatsu, M. (2021). NTCP Oligomerization Occurs Downstream of the NTCP-EGFR Interaction during Hepatitis B Virus Internalization. J. Virol. *95*, e00938-21. https://doi.org/10.1128/JVI.00938-21.
77. Fukano, K., Tsukuda, S., Oshima, M., Suzuki, R., Aizaki, H., Ohki, M., Park, S.-Y., Muramatsu, M., Wakita, T., Sureau, C., et al. (2018). Troglitazone Impedes the Oligomerization of Sodium Taurocholate Cotransporting Polypeptide and Entry of Hepatitis B Virus Into Hepatocytes. Front. Microbiol. *9*, 3257. https://doi.org/10.3389/fmicb.2018.03257.
78. Qin, T., Wang, Y., Nie, J., Yu, L., and Zeng, S. (2022). Oligomerization of the HBV/HDV functional receptor NTCP expressed in Sf9 insect cell. Biochim. Biophys. Acta Gen. Subj. *1866*, 130224. https://doi.org/10.1016/j.bbagen.2022.130224.
79. Ho, R.H., Leake, B.F., Roberts, R.L., Lee, W., and Kim, R.B. (2004). Ethnicity-dependent Polymorphism in Na+-taurocholate Cotransporting Polypeptide (SLC10A1) Reveals a Domain Critical for Bile Acid Substrate Recognition. J. Biol. Chem. *279*, 7213–7222. https://doi.org/10.1074/jbc.M305782200.
80. Liu, R., Chen, C., Xia, X., Liao, Q., Wang, Q., Newcombe, P.J., Xu, S., Chen, M., Ding, Y., Li, X., et al. (2017). Homozygous p.Ser267Phe in SLC10A1 is associated with a new type of hypercholanemia and implications for personalized medicine. Sci. Rep. *7*, 9214. https://doi.org/10.1038/s41598-017-07012-2.
81. Deng, M., Mao, M., Guo, L., Chen, F.-P., Wen, W.-R., and Song, Y.-Z. (2016). Clinical and molecular study of a pediatric patient with sodium taurocholate cotransporting polypeptide deficiency. Exp. Ther. Med. *12*, 3294–3300. https://doi.org/10.3892/etm.2016.3752.
82. Wang, Z., Zhu, Q., Liu, Y., Chen, S., Zhang, Y., Ma, Q., Chen, X., Liu, C., Lei, H., Chen, H., et al. (2021). Genome-wide association study of metabolites in patients with coronary artery disease identified novel metabolite quantitative trait loci. Clin. Transl. Med. *11*, e290. https://doi.org/10.1002/ctm2.290.
83. Chiang, J.Y.L. (2017). Bile acid metabolism and signaling in liver disease and therapy. Liver Res. *1*, 3–9. https://doi.org/10.1016/j.livres.2017.05.001.
84. Klenerman, P., and McMichael, A. (2007). AIDS/HIV: Finding Footprints Among the Trees. Science *315*, 1505–1507. https://doi.org/10.1126/science.1140768.
85. Kawashima, Y., Pfafferott, K., Frater, J., Matthews, P., Payne, R., Addo, M., Gatanaga, H., Fujiwara, M., Hachiya, A., Koizumi, H., et al. (2009). Adaptation of HIV-1 to human leukocyte antigen class I. Nature *458*, 641–645. https://doi.org/10.1038/nature07746.
86. Erdmann, N., Du, V.Y., Carlson, J., Schaefer, M., Jureka, A., Sterrett, S., Yue, L., Dilernia, D., Lakhi, S., Tang, J., et al. (2015). HLA Class-II Associated HIV Polymorphisms Predict Escape from CD4+ T Cell Responses. PLoS Pathog. *11*, e1005111. https://doi.org/10.1371/journal.ppat.1005111.
87. Ruhl, M., Knuschke, T., Schewior, K., Glavinic, L., Neumann-Haefelin, C., Chang, D.-I., Klein, M., Heinemann, F.M., Tenckhoff, H., Wiese, M., et al. (2011). CD8+ T-Cell Response Promotes Evolution of Hepatitis C Virus Nonstructural Proteins. Gastroenterology *140*, 2064–2073. https://doi.org/10.1053/j.gastro.2011.02.060.
88. Rauch, A., James, I., Pfafferott, K., Nolan, D., Klenerman, P., Cheng, W., Mollison, L., McCaughan, G., Shackel, N., Jeffrey, G.P., et al. (2009). Divergent adaptation of hepatitis C virus

genotypes 1 and 3 to human leukocyte antigen-restricted immune pressure. Hepatology *50*, 1017–1029. https://doi.org/10.1002/hep.23101.

89. Gaudieri, S., Rauch, A., Park, L.P., Freitas, E., Herrmann, S., Jeffrey, G., Cheng, W., Pfafferott, K., Naidoo, K., Chapman, R., et al. (2006). Evidence of Viral Adaptation to HLA Class I-Restricted Immune Pressure in Chronic Hepatitis C Virus Infection. JVI *80*, 11094–11104. https://doi.org/10.1128/JVI.00912-06.

90. Fitzmaurice, K., Petrovic, D., Ramamurthy, N., Simmons, R., Merani, S., Gaudieri, S., Sims, S., Dempsey, E., Freitas, E., Lea, S., et al. (2011). Molecular footprints reveal the impact of the protective HLA-A*03 allele in hepatitis C virus infection. Gut *60*, 1563–1571. https://doi.org/10.1136/gut.2010.228403.

91. Cao, L., Wu, C., Shi, H., Gong, Z., Zhang, E., Wang, H., Zhao, K., Liu, S., Li, S., Gao, X., et al. (2014). Coexistence of Hepatitis B Virus Quasispecies Enhances Viral Replication and the Ability To Induce Host Antibody and Cellular Immune Responses. J. Virol. *88*, 8656–8666. https://doi.org/10.1128/JVI.01123-14.

The American Journal of Human Genetics, Volume *111*

Supplemental information

Joint host-pathogen genomic analysis identifies hepatitis B virus mutations associated with human *NTCP* and HLA class I variation

Zhi Ming Xu, Gnimah Eva Gnouamozi, Sina Rüeger, Patrick R. Shea, Maria Buti, Henry LY. Chan, Patrick Marcellin, Dylan Lawless, Olivier Naret, Matthias Zeller, Arne Schneuing, Andreas Scheck, Thomas Junier, Darius Moradpour, Ondrej Podlaha, Vithika Suri, Anuj Gaggar, Mani Subramanian, Bruno Correia, David Gfeller, Stephan Urban, and Jacques Fellay

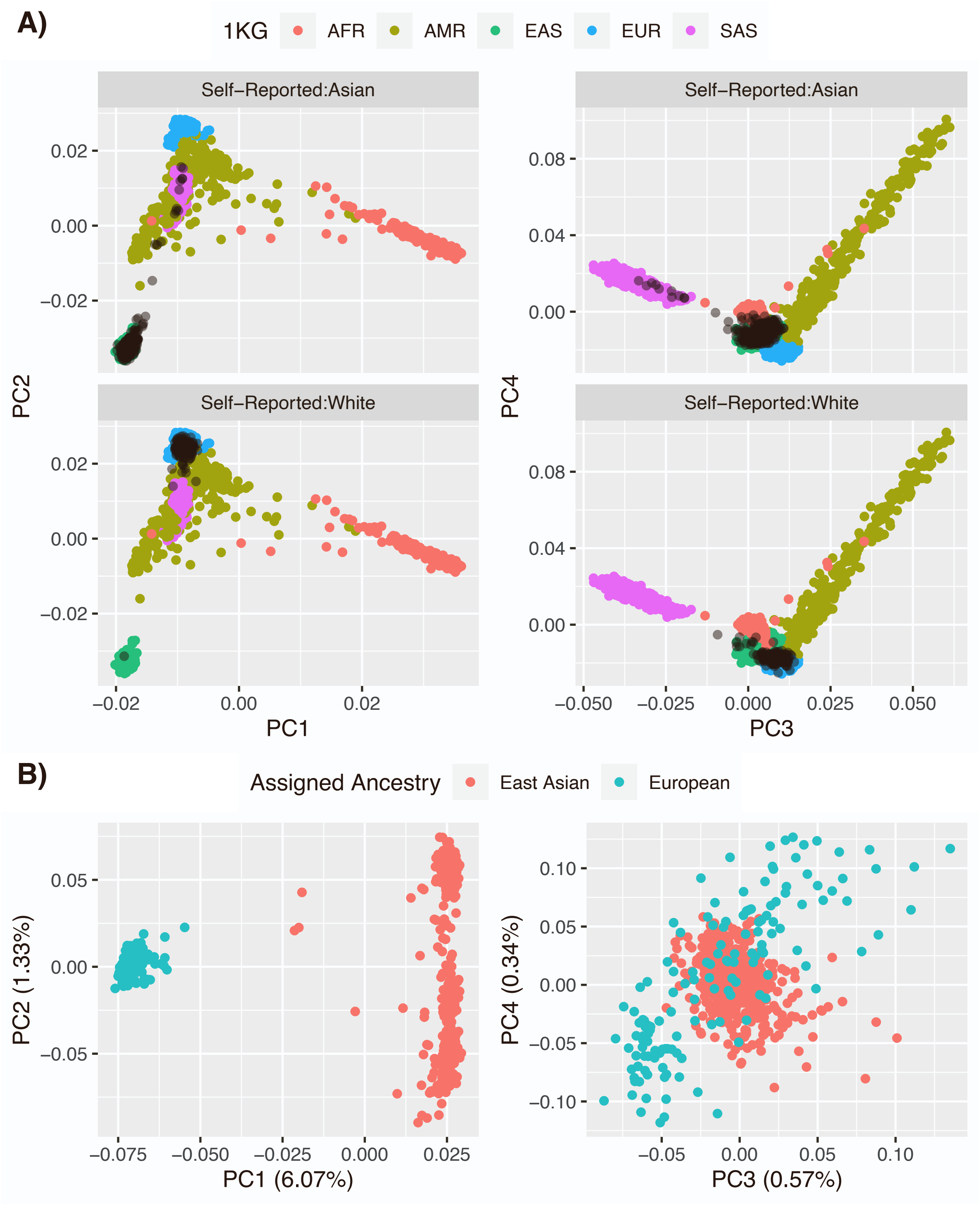

Figure S1: PCA representing human ancestry. a) Principal components based on 1000 Genomes (1KG) samples. Study participants were overlaid in the same principal component space using loadings derived from 1KG. Coloured dots represent 1KG populations and black dots represent study participants. Facet grids represent the self-reported race of study participants. **b)** Principal components re-calculated based on only retained study participants within the East Asian and European ancestry groups. Colors indicate assigned ancestry based on hierarchical clustering applied to 1KG based principal components.

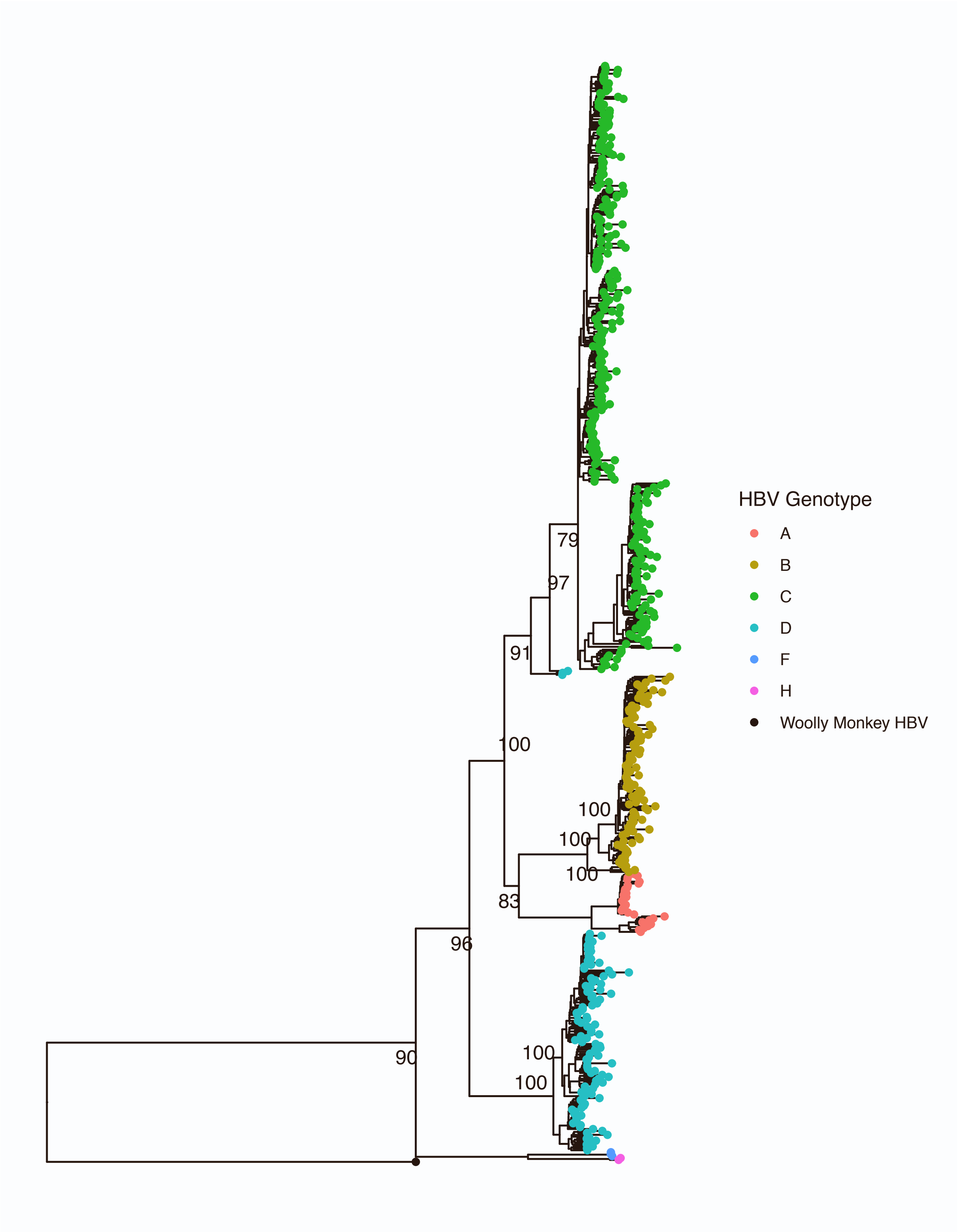

Figure S2: Phylogenetic tree of HBV sequences. Phylogenetic tree constructed using a maximum likelihood-based algorithm (IQ-TREE) from multiple sequence alignment of HBV sequences with Woolly Monkey HBV as the outgroup. Colours indicate HBV genotype. Bootstrap support values were calculated using UFBoot with 1000 replicates.

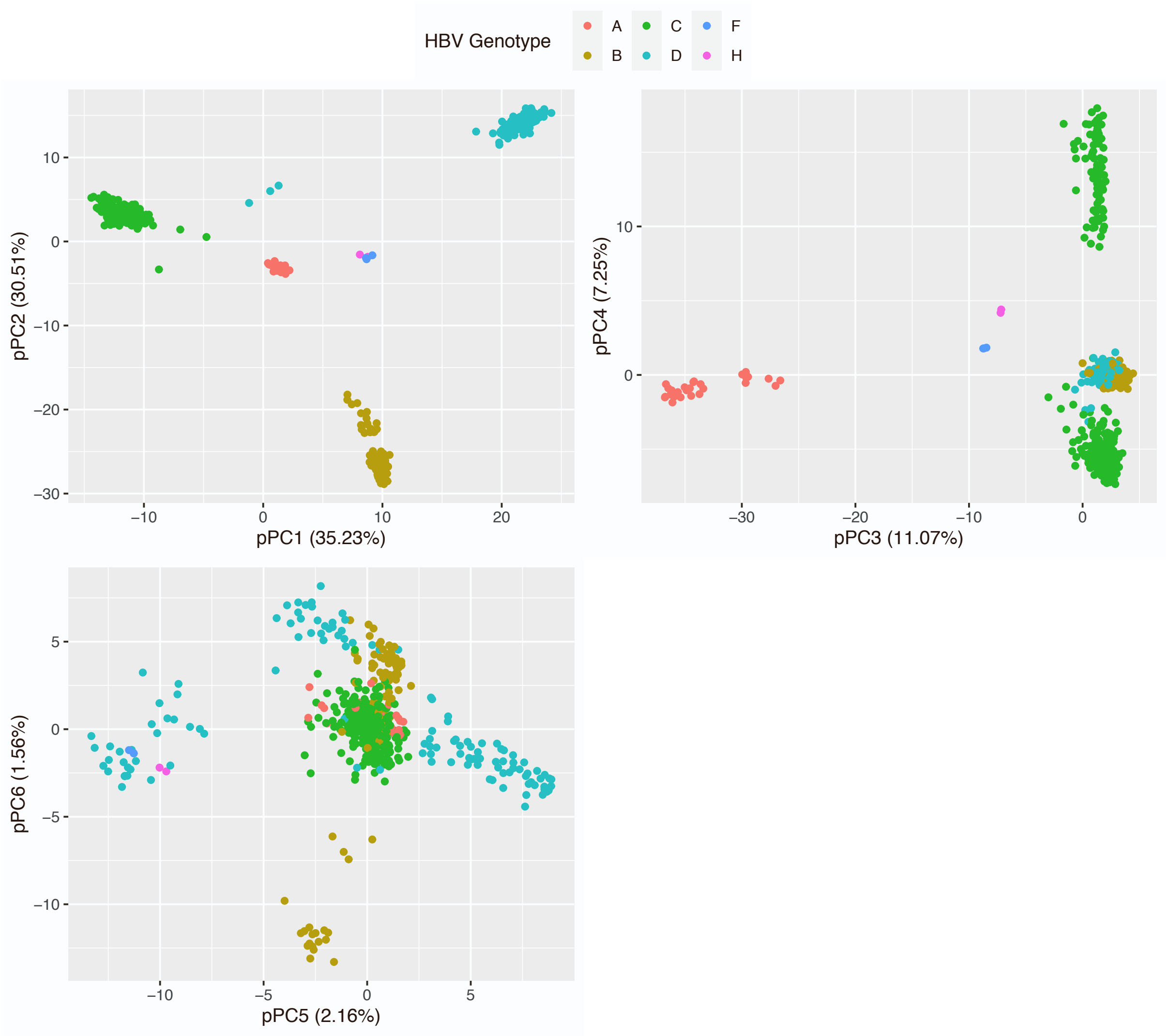

Figure S3: Phylogenetic principal component. Phylogenetic principal components (pPCs) derived from HBV amino acid variants and maximum-likelihood phylogenetic tree. Colors represent HBV genotypes and the percent of variance explained are indicated in brackets for each pPC.

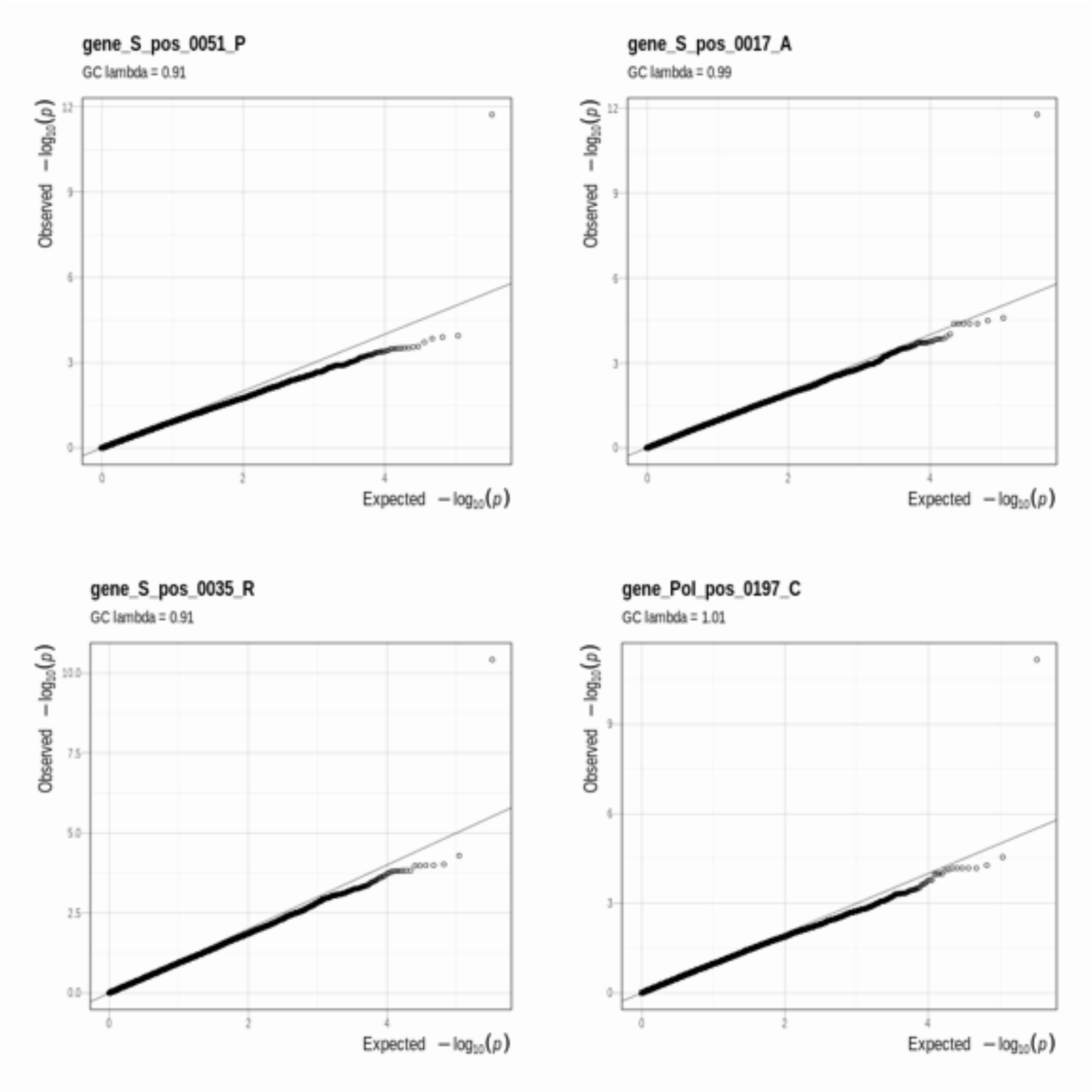

Figure S4: QQ plots for GWASs conducted on HBV variants which are associated with rs2296651. Observed p-values against expected p-values under the null chi-squared distribution.

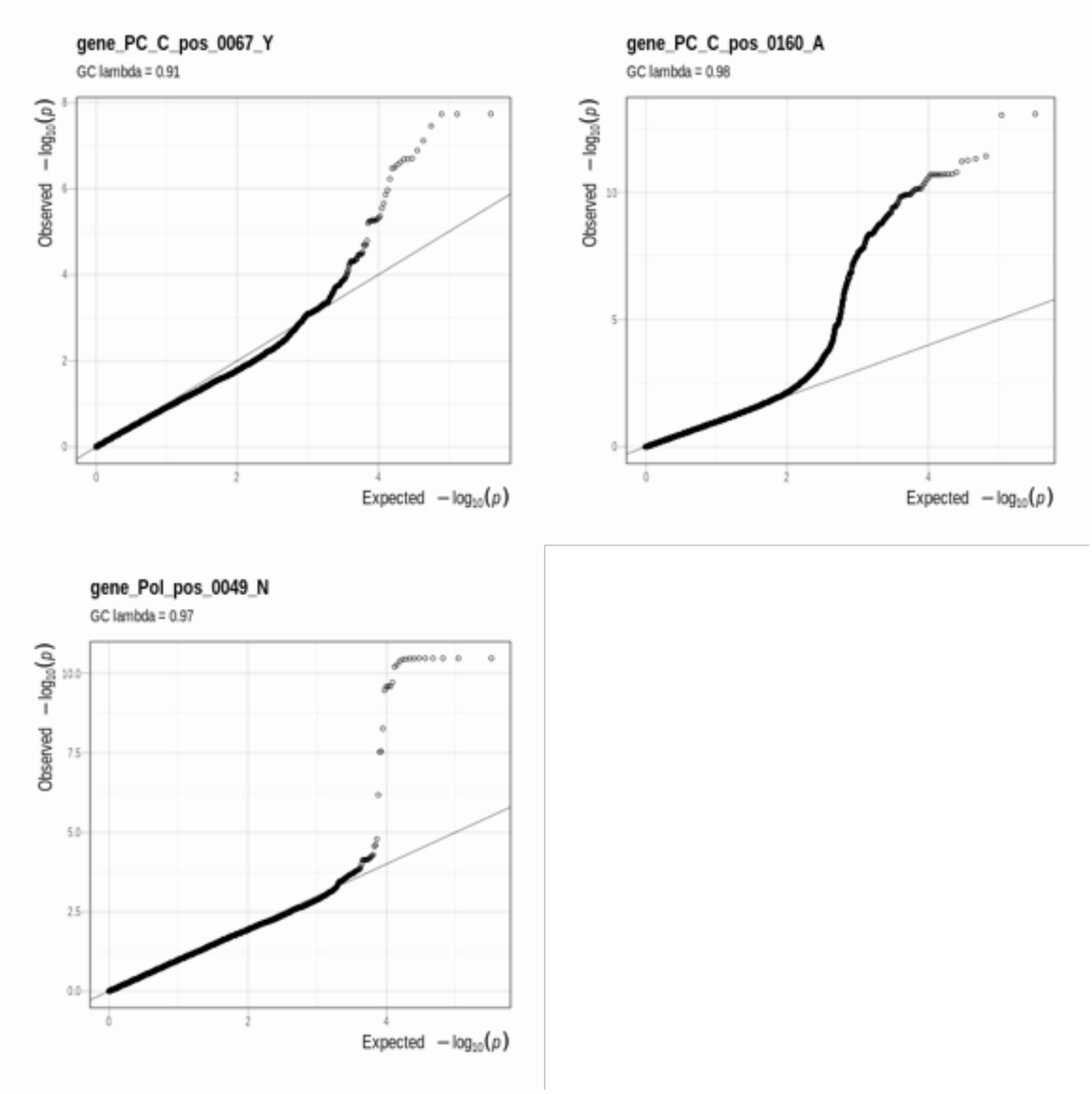

Figure S5: QQ plots for GWASs conducted on HBV variants that are associated with HLA Class I variants. Observed p-values against expected p-values under the null chi-squared distribution.

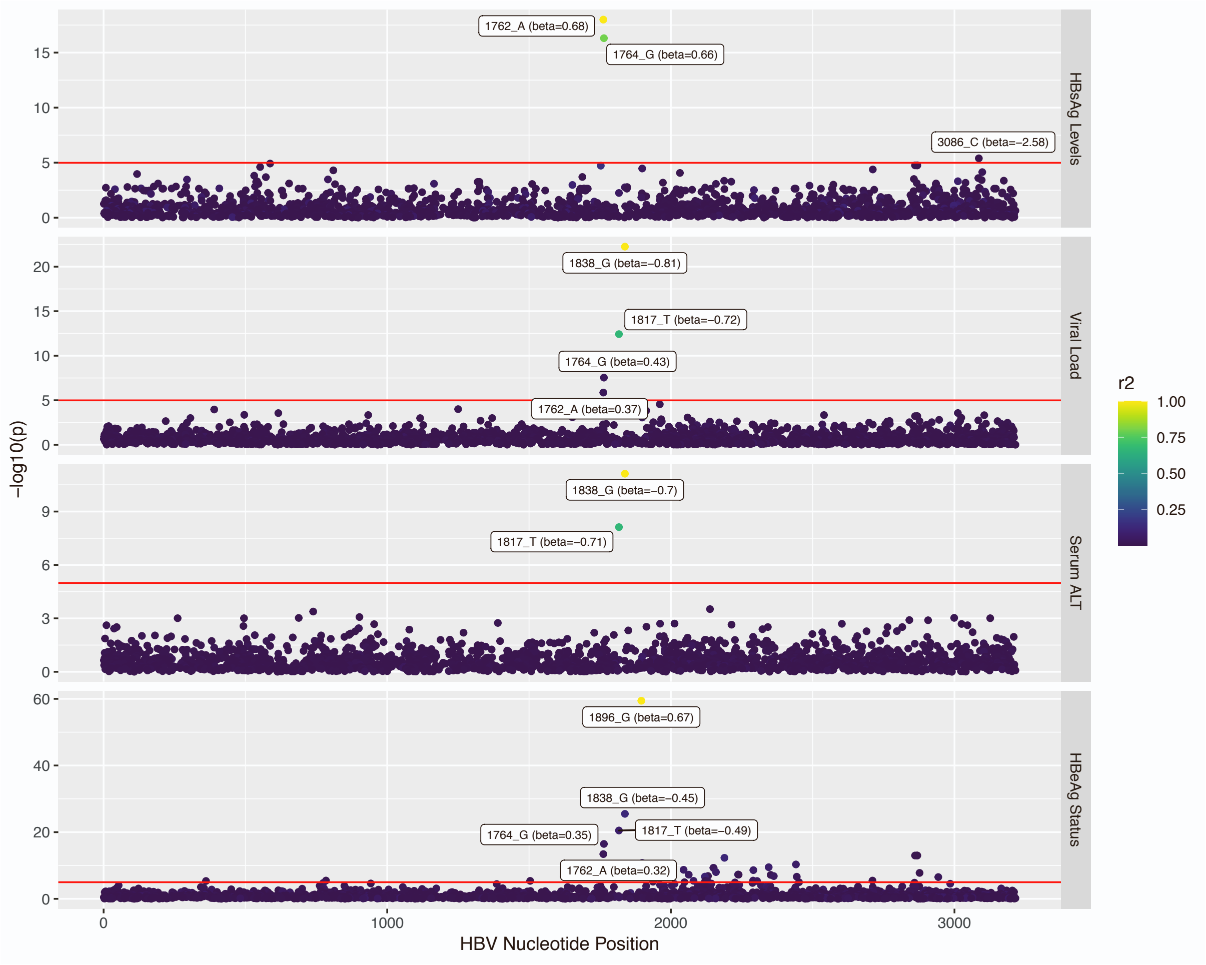

Figure S6: HBV nucleotide variants associated with markers of HBV infection. Variants were considered present if detected above 15% within the intra-host viral population. Quantitative markers of HBV infection were inverse-normal transformed and regressed against nucleotide variants using linear regression adjusting for age, sex, HBeAg status, top 4 human principal components, and top 6 phylogenetic principal components. HBeAg was regressed against nucleotide variants using logistic regression adjusting for age, sex, top 4 human principal components, and top 6 phylogenetic principal components. Red line shows Bonferroni corrected significance threshold of 1.03×10^{-5} (4858 tested nucleotide variants). Color represents the strength of linkage disequilibrium with the strongest association.

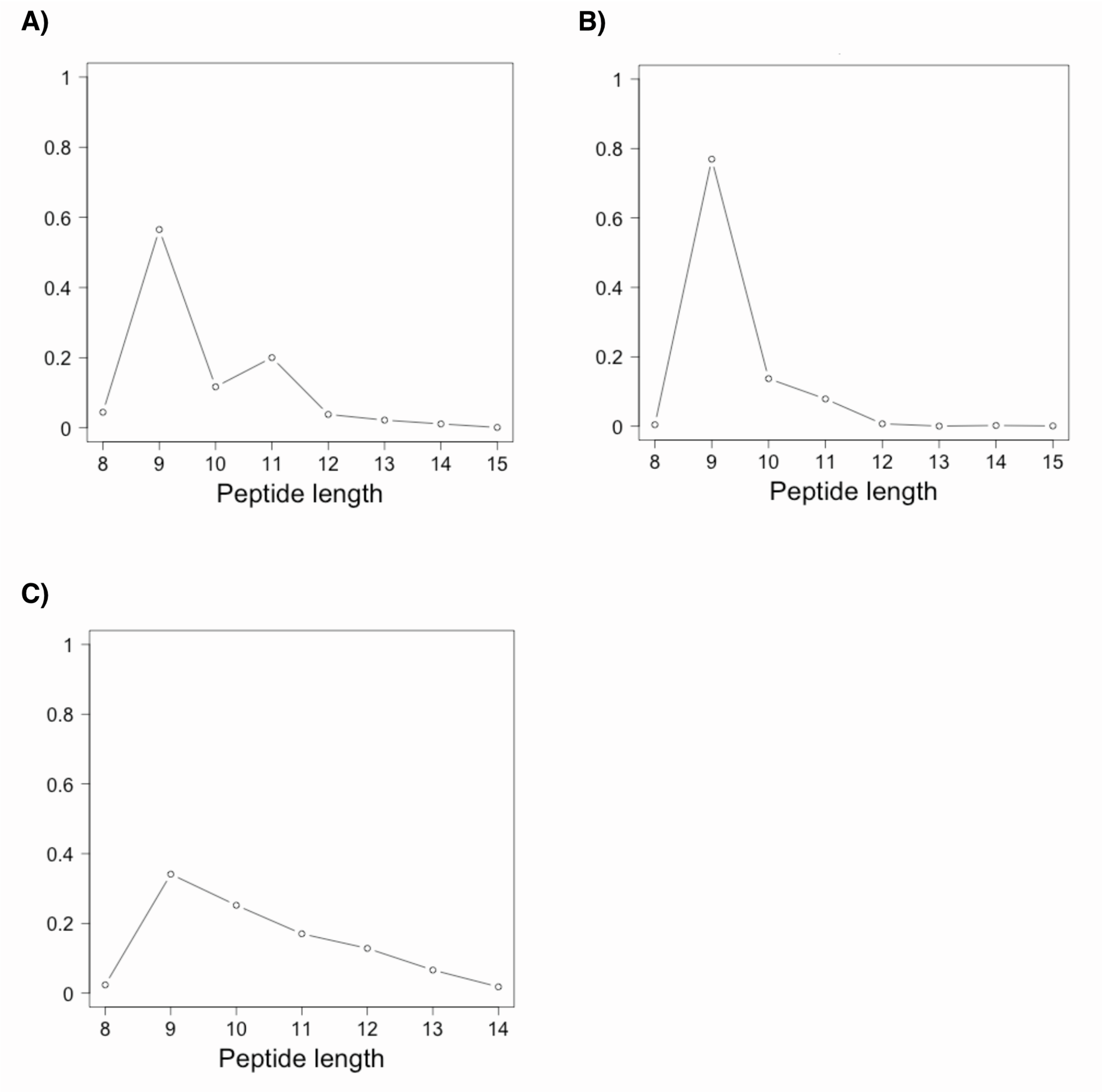

Figure S7: Peptide length distribution for HLA-A alleles. A) HLA-A*33:03 **B)** HLA-A*02:06 **C)** HLA-A*01:01

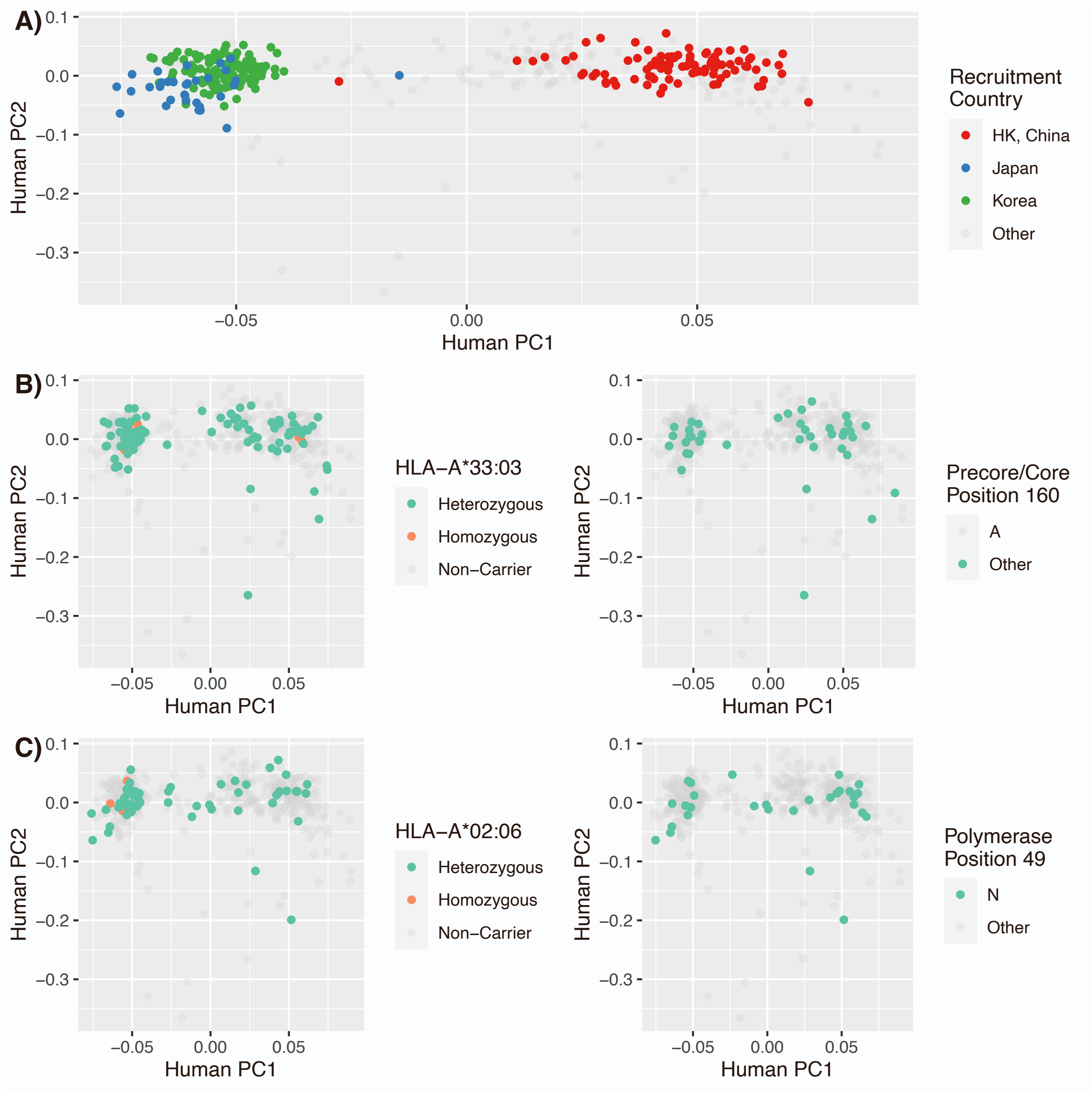

Figure S8: Prevalence of HLA-alleles and associated HBV mutations within the East Asian cohort. A) Human genetic principal components (PCs) capture genetic differences of individuals recruited from different East Asian countries, revealing ancestry subgroups. **B)** Both HLA-A*33:03 and the associated HBV mutation (Precore/Core position 160) are present across multiple recruitment countries and ancestry subgroups. **C)** Both HLA-A*02:06 and the associated HBV mutation (Polymerase position 49) are present across multiple recruitment countries and ancestry subgroups.

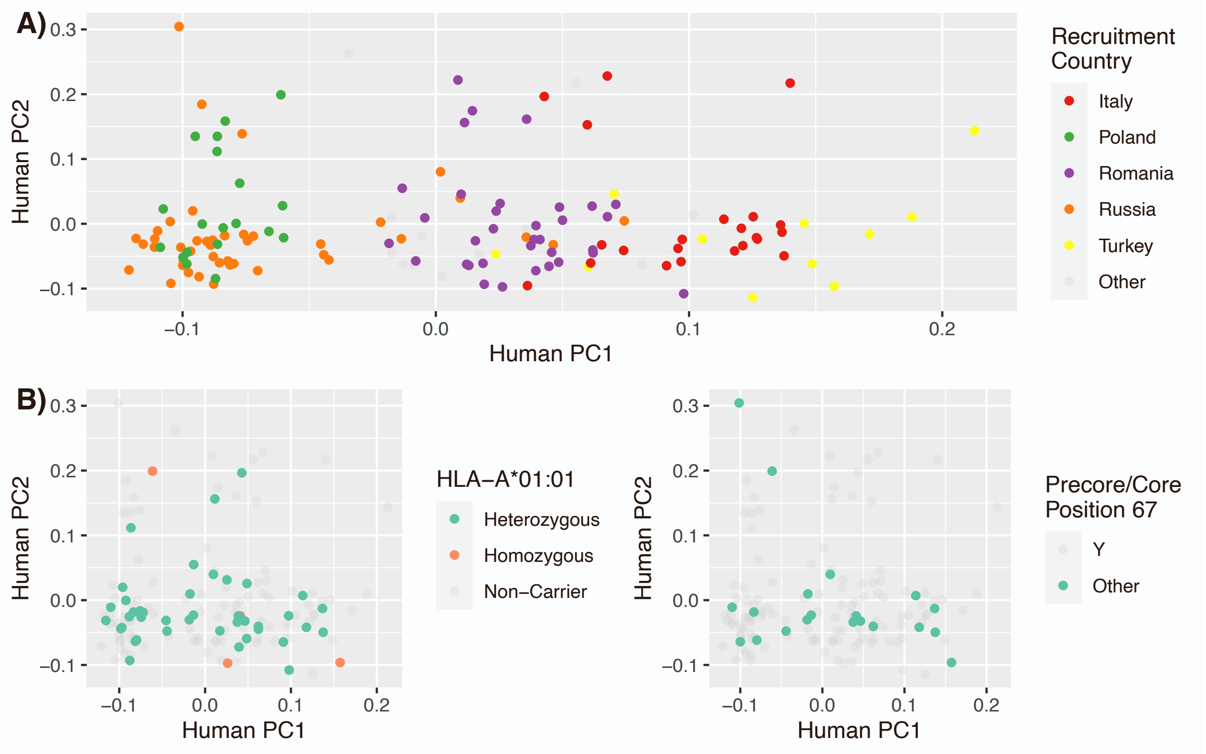

Figure S9: Prevalence of HLA-alleles and associated HBV mutations within the European cohort. **A)** Human genetic principal components (PCs) capture genetic differences of individuals recruited from different European countries, revealing ancestry subgroups. **B)** Both HLA-A*01:01 and the associated HBV mutation (Precore/Core position 67) are present across multiple recruitment countries and ancestry subgroups.

	Human_PC1	Human_PC2	Human_PC3	Human_PC4	phylo_PC1	phylo_PC2	phylo_PC3	phylo_PC4	phylo_PC5	phylo_PC6
HLA–A*33:03	0.01	0	0	0	0.01	0	0	0	0.01	0.01
HLA–A*02:06		0	0	0	0.01	0.01	0	0.01	0	0
Precore/Core Pos 160 (AA:A)			0	0	0	0	0	0.02	0.02	0.02
Polymerase Pos 49 (AA:N)				0	0	0.01	0	0	0	0

	Human_PC1	Human_PC2	Human_PC3	Human_PC4	phylo_PC1	phylo_PC2	phylo_PC3	phylo_PC4	phylo_PC5	phylo_PC6
HLA–A*01:01	0	0.01	0.01	0.01	0	0	0	0.01	0	0.01
Precore/Core Pos 67 (AA:Y)		0	0.01	0	0.02	0	0	0.02	0	0.05

Figure S10: Correlation between HLA-A alleles or association HBV mutations and human or phylogenetic principal components. Top panel refers to associations identified in the East Asian cohort, and bottom panel associations identified in the European cohort. Values indicate coefficient of determination (r^2) calculated using Point-Biserial correlation.

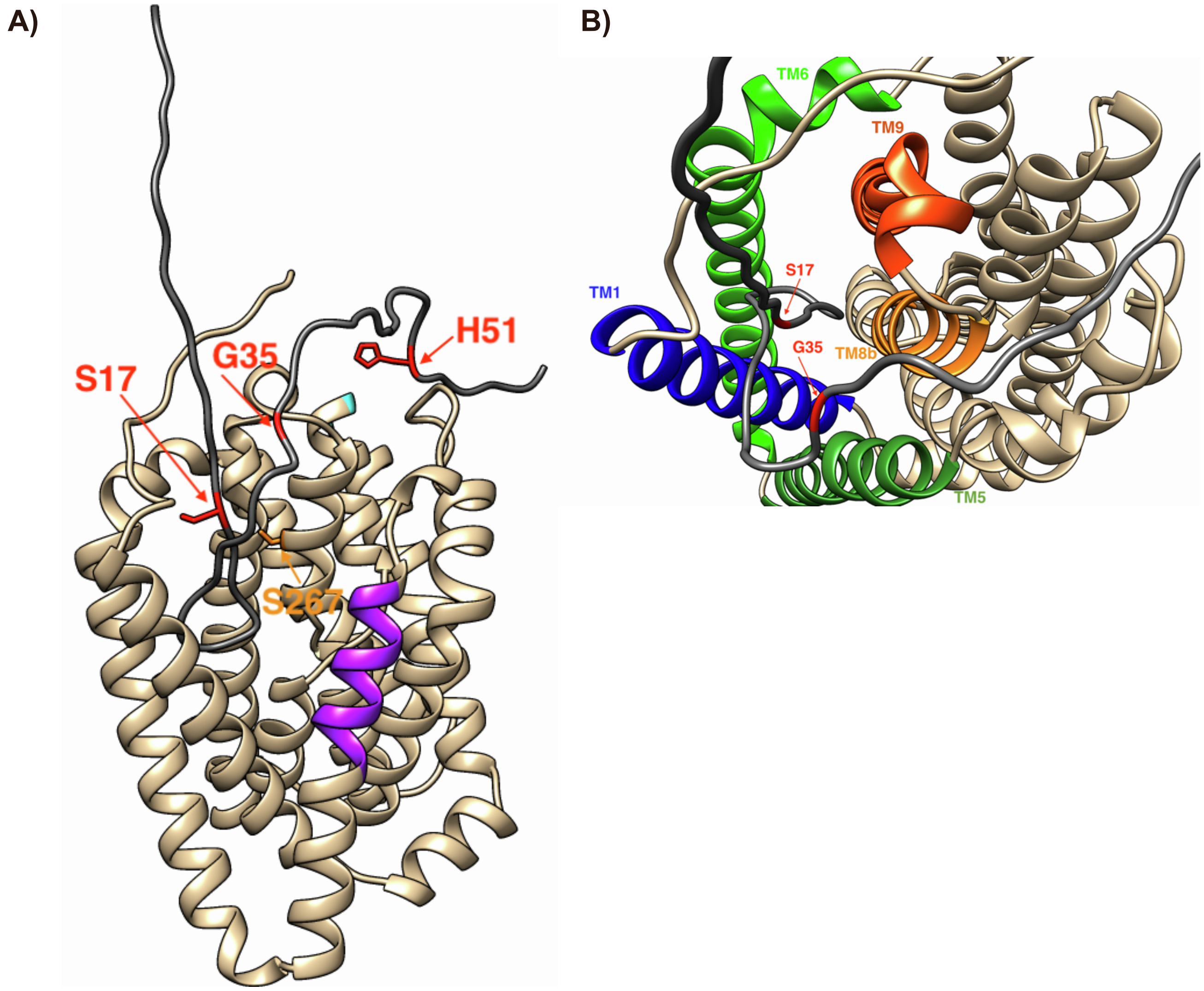

Figure S11: Top ranking model of preS1-NTCP binding based on AlphaFold-Multimer. The wildtype NTCP sequence (in tan) and a peptide (in grey) derived from HBV preS1 (aa 2-60) based on the Genotype C consensus sequence was used to construct the model. **A)** Position of the human variant (NTCP S267F) is labelled in orange, and positions of associated preS1 residues (position S17, G35, and H51) are labelled in red. Patch 1 (aa 84-87, in cyan) and patch 2 (aa 157-167, in purple) of NTCP are regions outside of the NTCP tunnel that were previously reported to be also important for preS1 binding. **B)** Zoom-in view representing extracellular view of the NTCP heterozygous, with domains color coded according to the published EM-structure (Asami et al, 2022).

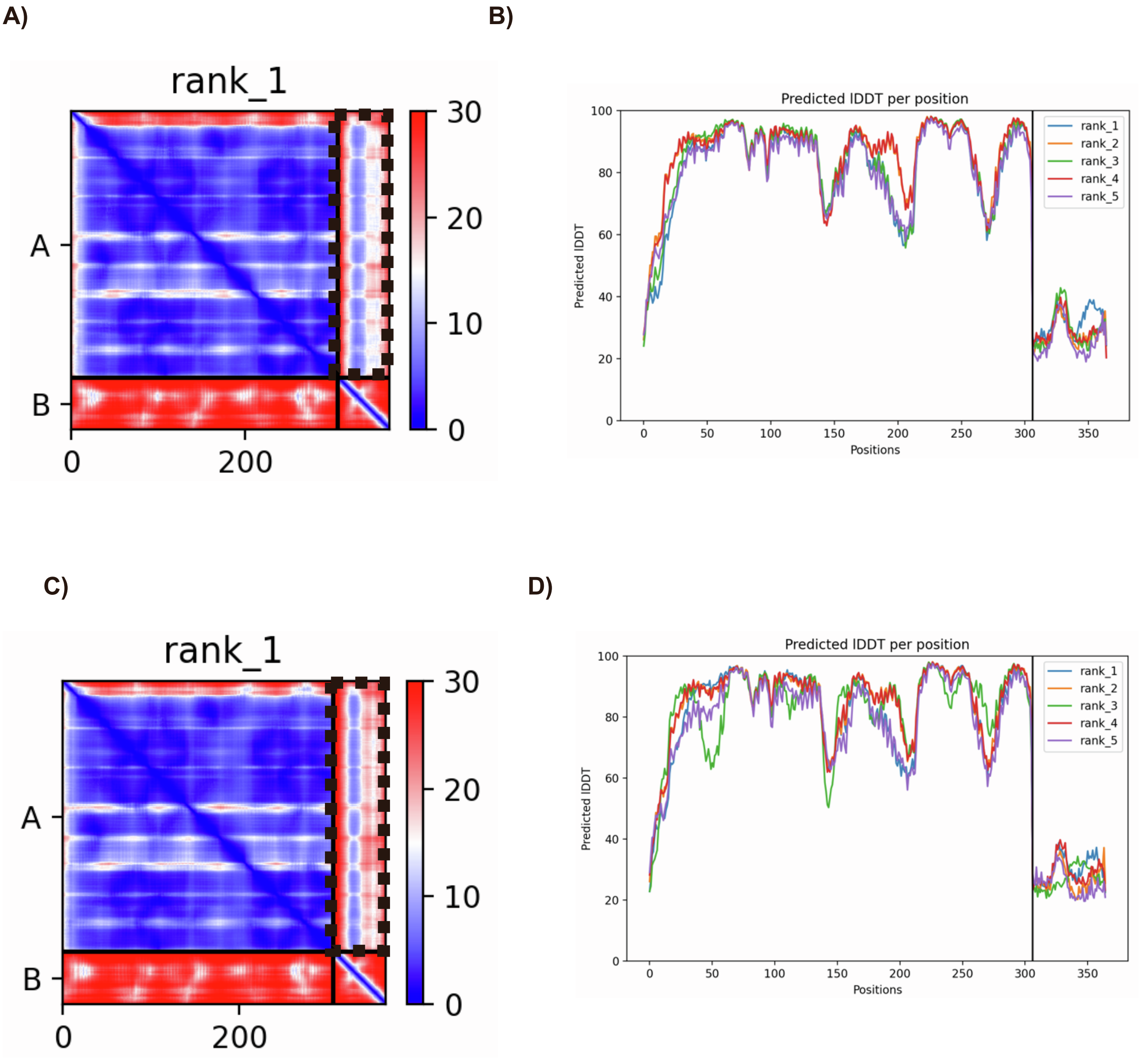

Figure S12: Predicted aligned error (PAE) score matrices and predicted local distance difference test (pLDDT) scores of AlphaFold-Multimer preS1-NTCP models. The PAE score is a per-residue accuracy metric between pairs of residues (x,y). The dotted box refers to residues within the matrix that were used to calculate the average PAE for the preS1-NTCP interface. The pLDDT is a per-residue accuracy metric of the local confidence of the structure. The NTCP residues are left of the vertical line and the HBV preS1 residues are right of the vertical line. **A)** PAE of top-ranking model based on NTCP WT and preS1 WT **B)** pLDDT of models based on NTCP WT and preS1 WT **C)** PAE of top-ranking model based on NTCP S267F and preS1 WT **D)** pLDDT of models based on NTCP S267F and preS1 WT.

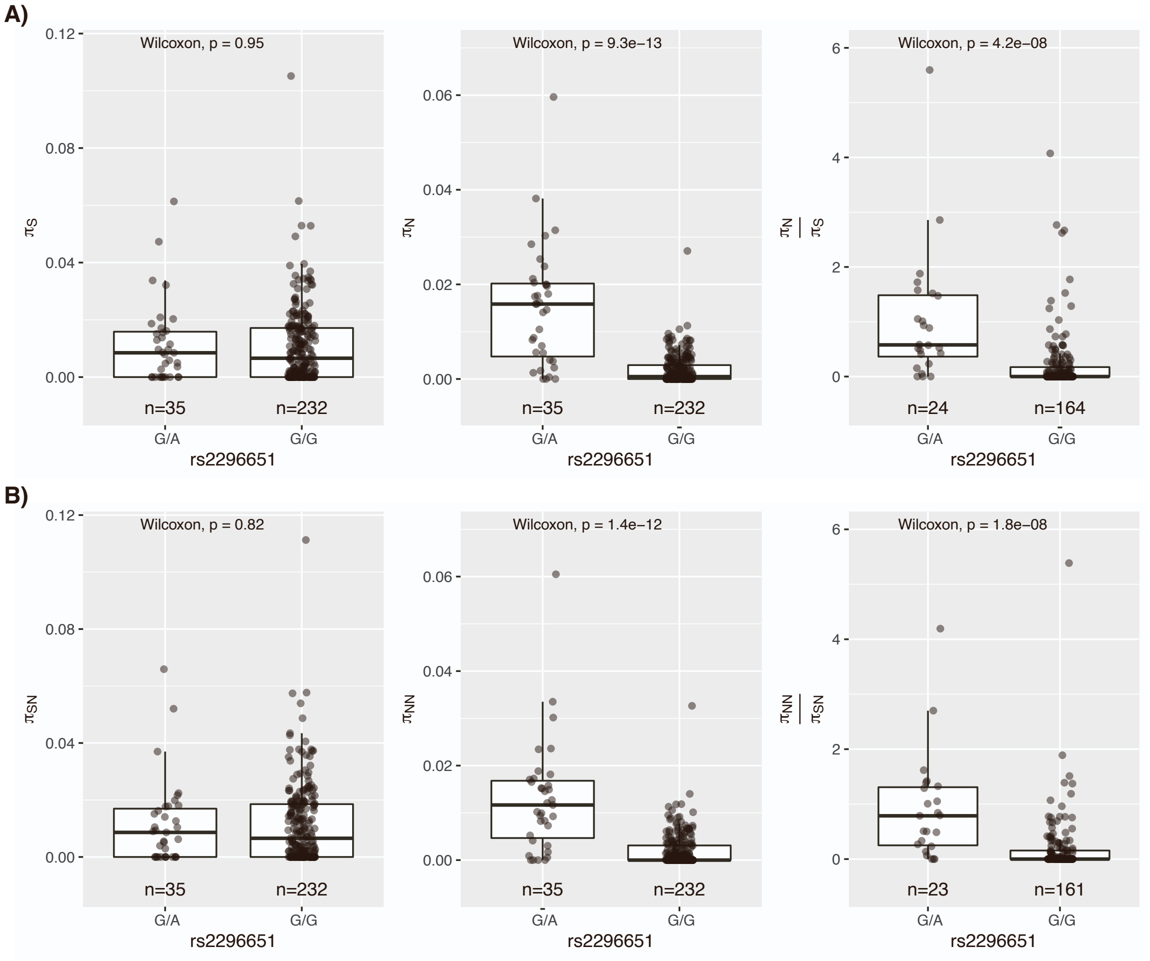

Figure S13: Intra-host non-synonymous and synonymous nucleotide diversity within the preS1 receptor binding region in heterozygous NTCP S267F carriers (rs2296551-G/A) and homozygous NTCP WT carriers (rs2296551-G/G) A) Substitutions and sites are included irrespective of consequence in the polymerase reading frame. π_N represents non-synonymous and π_s represents synonymous nucleotide diversity. **B)** Substitutions and sites are restricted to those that are non-synonymous in the polymerase reading frame.

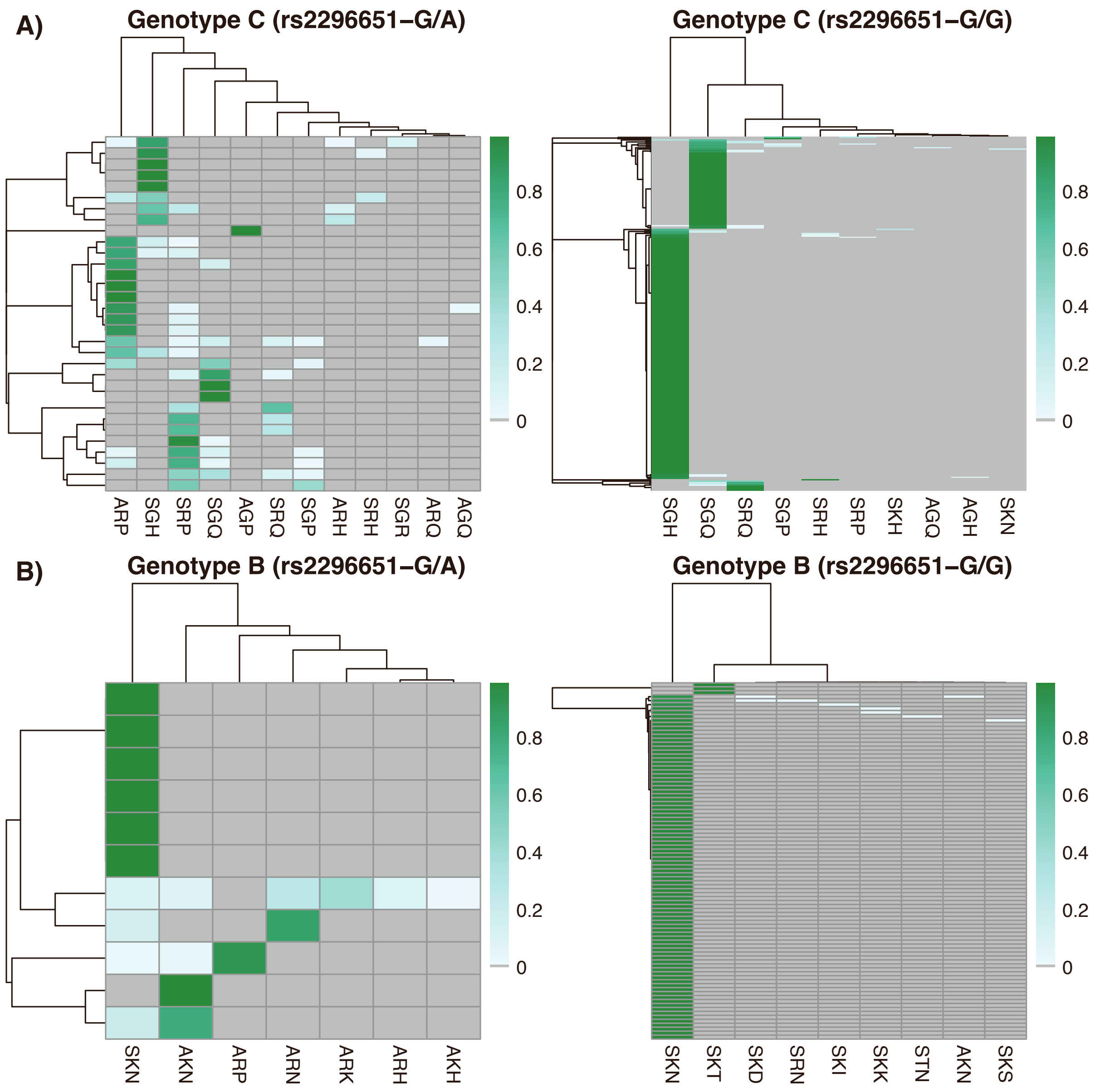

Figure S14: Heatmap of the intra-host composition of HBV preS1 receptor-binding region haplotypes. Haplotypes are grouped according to residues at position 17, 31, and 51 of preS1. Colors indicate the proportion of reads supporting each haplotype within each individual. An entry is grey if the haplotype is absent in the individual.

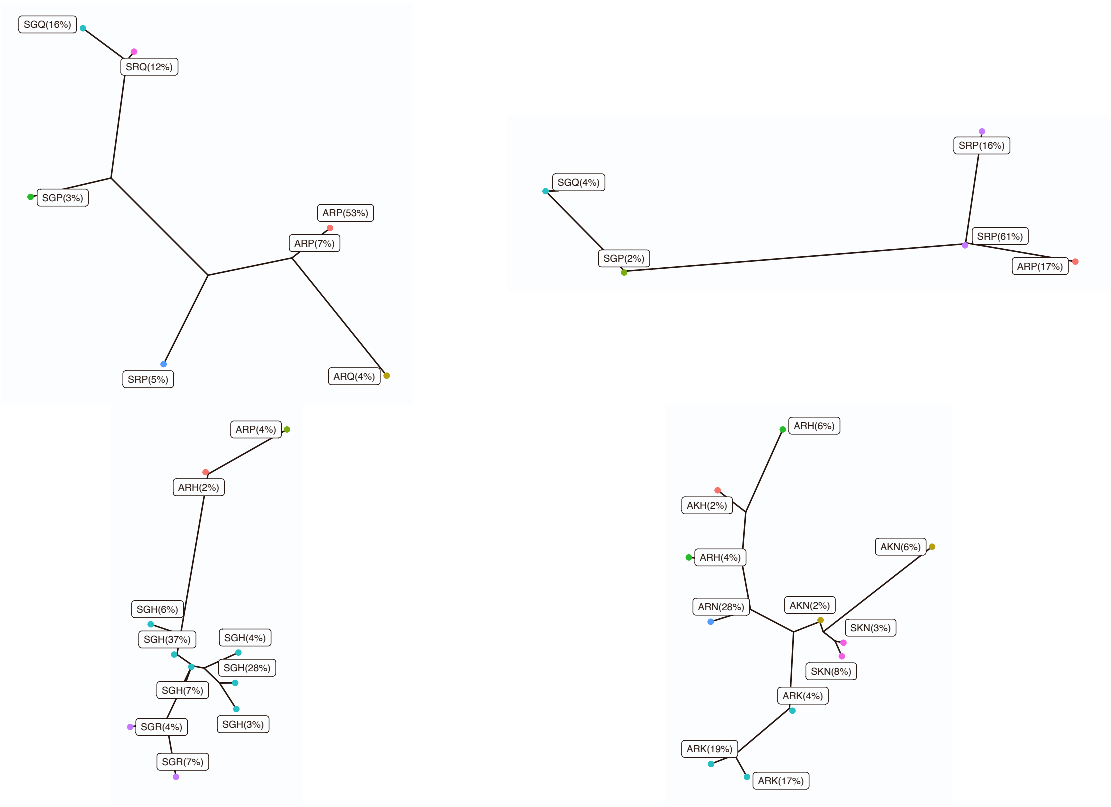

Figure S15: Examples of intra-host evolutionary trajectories. Unrooted tree constructed based on preS1 binding domain nucleotide sequences using a neighbour-joining algorithm. Labels indicate haplotypes based on residues at positions 17,35,51 of preS1. The proportion of intra-host reads supporting each haplotype is indicated in brackets.

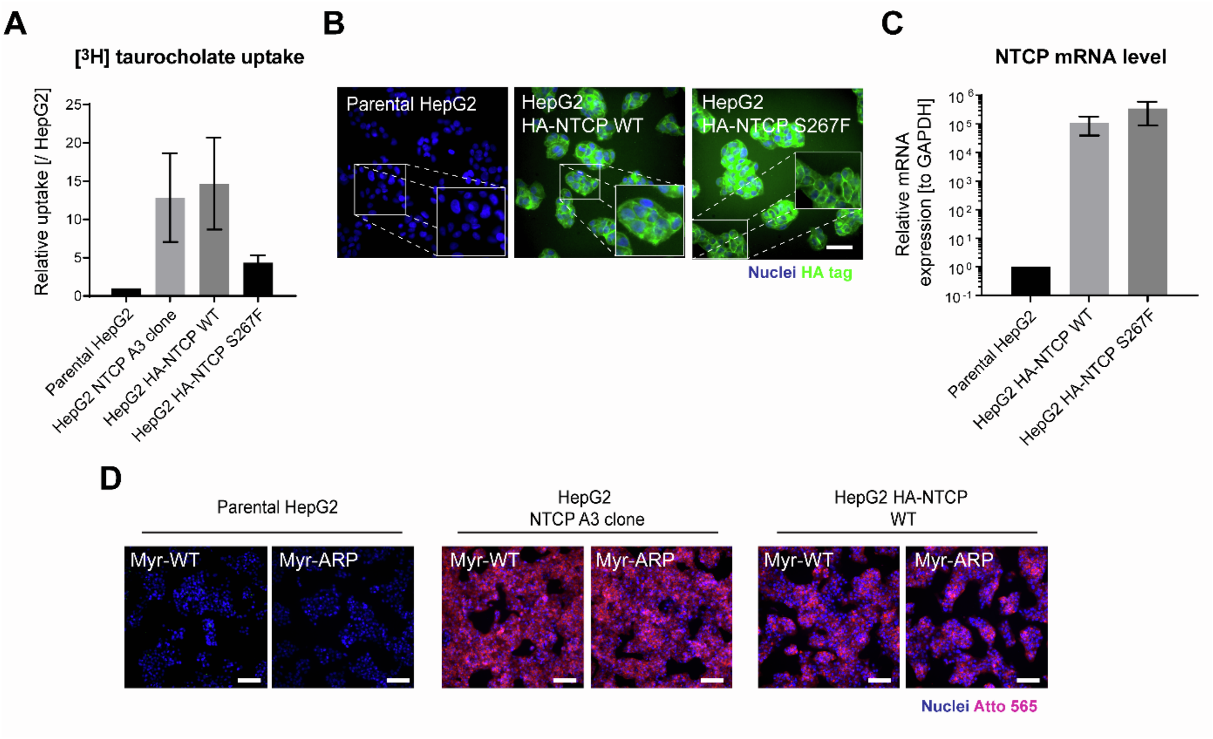

Figure S16: Validation and characterization of NTCP expression in HepG2 HA-NTCP and HA-NTCP S267F stable cell lines and synthesis of myristoylated peptides for affinity binding studies. A) After antibiotic selection, the functionality of NTCP in stable expressing cell lines was evaluated via taurocholate uptake assay comparing parental HepG2 (negative control), HepG2 NTCP A3 clone (positive control), HepG2 NTCP-WT or HepG2 NTCP-S267F cells. **B)** HA- NTCP expressing cell lines, were stained with mAb anti-HA tag and with Alexa 488-conjugated goat anti-mouse IgG, scale bar: 100µm **C**) Intracellular NTCP mRNA expression was measured by RT- qPCR. NTCP expression was calculated relative to housekeeping gene GAPDH and normalized on the expression in parental HepG2 cells. **D**) The binding capacity of the synthetized peptides was evaluated performing binding assay using parental HepG2, HepG2 NTCP A3 clone and HepG2 HA- NTCP cells scale bar: 100µm.

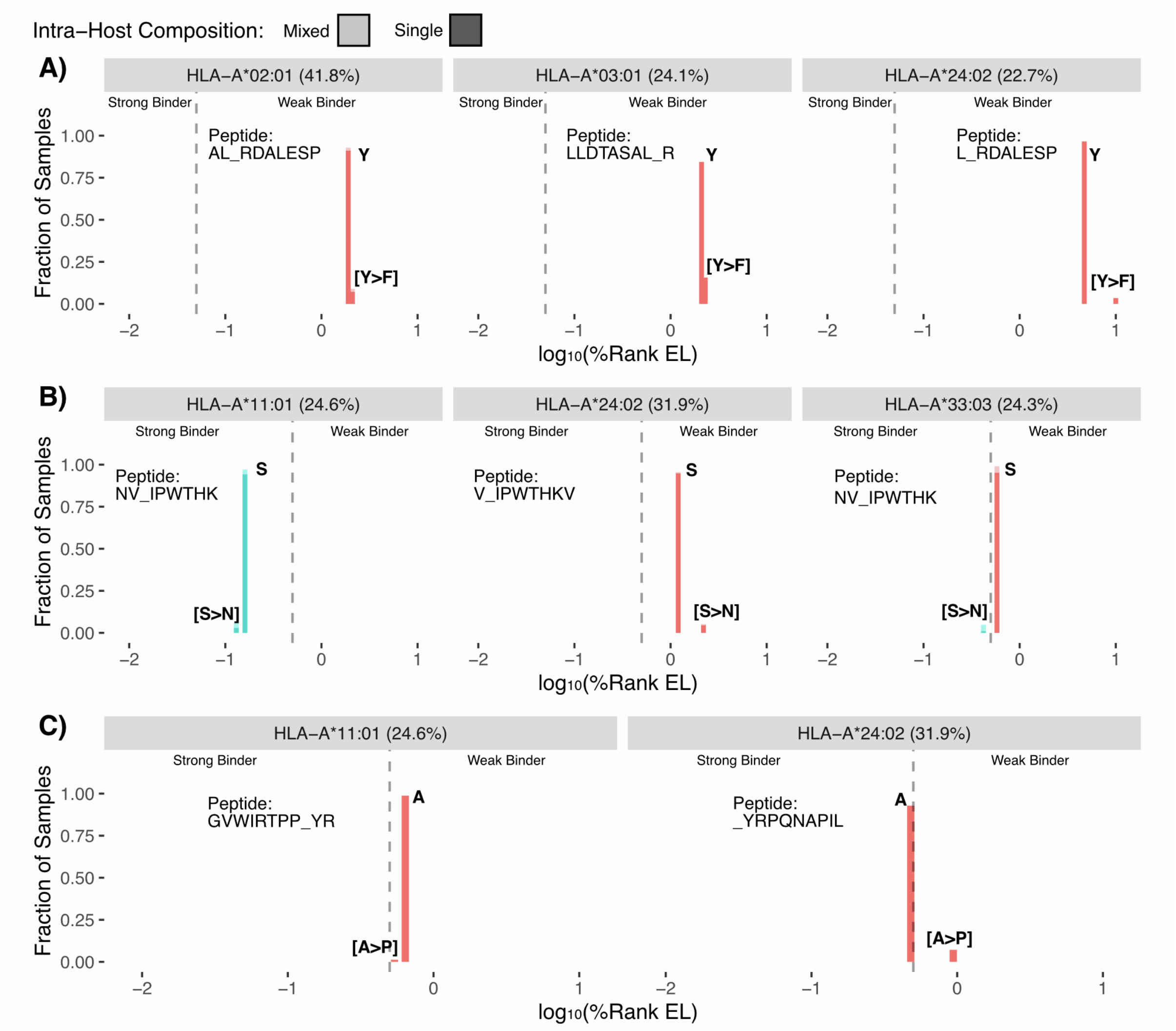

Figure S17: Impact of HBV amino acid mutations on binding affinity to other prevalent HLA-alleles. Each panel refers to the binding affinities of peptides to HLA-A alleles that are prevalent in the cohort (> 20% frequency) but not associated with the HBV position. Peptide amino acid sequence is shown above each bar and the relative position of the HLA-associated HBV position is indicated in bold. X-axis represents the binding affinity of each peptide, and y-axis represents the fraction of samples within carriers of the specific HLA-A allele for which the peptide is present. Shading represents whether the peptide is present as part of a mixture in the intra-host level (multiple peptides in the intra-host viral population) or not. The dotted lines indicate the threshold of a strong binder, defined as elution percentile rank less than 0.5% **(A)** Referring to position 67 of HBV precore/core protein in the European cohort. **(B)** Referring to position 49 of HBV polymerase protein in the East Asian cohort. **(C)** Referring to position 160 of HBV precore/core protein in the East Asian cohort.

Table S1: Summary of demographic characteristics of trial participants included in this study.

Clinical Trial ID	Time of Study	Key Inclusion Criteria	Key Exclusion Criteria
NCT02579382	Nov 2015 – Aug 2017	- Aged 18-65 years<br>- Chronic hepatitis B (HBsAg positivity of >6 months)<br>- HBV DNA ≥2000 IU/ml	- Extensive bridging fibrosis or cirrhosis<br>- Oral antiviral treatment within 3 months of screening<br>- Coinfections with HCV, HIV, or HDV
NCT01940341	Sept 2013 – Oct 2014	- Aged at least 18 years<br>- Chronic hepatitis B<br>- HBeAg negative and HBeAb positive<br>- HBV DNA ≥2000 IU/ml<br>- Serum alanine aminotransferase concentrations of > 60 U/L in men or >38 U/L in women and <10x the upper limit of the normal range.<br>- Treatment-naive or treatment-experienced, provided they meet entry criteria	- Evidence of hepatocellular carcinoma or decompensation.<br>- Treatment with interferon within 6 months<br>- Coinfections with HCV, HIV, or HDV
NCT01940341	Sept 2013 – Dec 2014	- Aged at least 18 years<br>- Chronic hepatitis B<br>- HBeAg positive<br>- Serum alanine aminotransferase concentrations of > 60 U/L in men or >38 U/L in women and <10x the upper limit of the normal range.<br>- Treatment-naive or treatment-experienced, provided they meet entry criteria	- Evidence of hepatocellular carcinoma or decompensation.<br>- Treatment with interferon within 6 months<br>- Coinfections with HCV, HIV, or HDV
NCT02174276	July 2014 – Aug 2016	- Aged at least 18 years<br>- Documented evidence of chronic hepatitis B virus (HBV) infection<br>- HBV DNA ≥2000 IU/ml	- Cirrhosis, inadequate liver function, or hepatocellular carcinoma.<br>- Therapy with immunomodulators, biologics, or investigational agents within 3 months of screening.<br>- Coinfections with HCV, HIV, or HDV

Table S2: Association between HLA-A alleles and HBV amino acid variants in the East Asian cohort. Top association annotated in bold.

		Precore/core: Position 160 (Associated AA: A)			Polymerase: Position 49 (Associated AA: N)		
**HLA Allele**	**Frequency**	**P**	**OR**	**SE**	**P**	**OR**	**SE**
HLA-A*24:02	0.3190	1.85e-01	1.6300	0.367	5.17e-01	0.784	0.376
HLA-A*11:01	0.2460	1.47e-03	4.2200	0.453	6.36e-01	0.800	0.471
HLA-A*33:03	0.2430	**2.10e-10**	**0.0775**	**0.402**	4.63e-01	0.735	0.419
HLA-A*02:01	0.1730	1.17e-01	2.1500	0.487	7.02e-01	0.817	0.529
HLA-A*02:07	0.1470	4.29e-01	1.5200	0.527	1.82e-01	0.475	0.558
HLA-A*02:06	0.1390	1.17e-01	2.1600	0.490	**1.20e-12**	**84.200**	**0.624**
HLA-A*02:03	0.0898	1.96e-01	2.1700	0.598	3.30e-02	0.276	0.604
HLA-A*31:01	0.0827	7.92e-02	0.3290	0.634	4.11e-01	0.533	0.767
HLA-A*11:50	0.0757	3.86e-02	4.7100	0.749	9.52e-02	0.274	0.776
HLA-A*26:01	0.0709	7.69e-01	1.2500	0.755	2.95e-01	0.460	0.740
HLA-A*11:32	0.0449	3.65e-01	2.3800	0.957	5.13e-01	1.880	0.964
HLA-A*11:02	0.0307	5.12e-01	0.4940	1.080	8.39e-01	1.270	1.190
HLA-A*30:01	0.0260	3.91e-01	0.3910	1.100	3.67e-01	0.309	1.300

Table S3: Association between HLA-A alleles and HBV amino acid variants in the European cohort. Top association annotated in bold.

		Precore/core: Position 67 (Associated AA: Y)		
**HLA Allele**	**Frequency**	**P**	**OR**	**SE**
HLA-A*02:01	0.4180	6.13e-02	2.5900	0.510
HLA-A*01:01	0.3260	**9.74e-08**	**0.0676**	**0.505**
HLA-A*03:01	0.2410	7.59e-01	1.1800	0.534
HLA-A*24:02	0.2270	2.18e-01	2.3400	0.691
HLA-A*32:01	0.0709	8.30e-01	1.2800	1.140
HLA-A*26:01	0.0638	2.73e-01	3.9000	1.240
HLA-A*30:01	0.0567	2.28e-01	3.7100	1.090
HLA-A*31:01	0.0567	3.52e-01	3.5900	1.380
HLA-A*68:01	0.0496	2.64e-01	3.8000	1.200
HLA-A*23:01	0.0496	7.24e-01	1.5500	1.230
HLA-A*11:32	0.0426	3.75e-01	3.6500	1.460
HLA-A*11:01	0.0355	2.15e-01	0.0828	2.010
HLA-A*11:50	0.0355	8.62e-01	0.7880	1.370

Table S4: Association between HLA-A amino acid positions and HBV amino acid variants in the East Asian cohort. P-values based on the omnibus test. The conditional p-value represents the association conditioned on the top association (in bold).

		Precore/core position 160 (Associated AA: A)		Pol position 49 (Associated AA: N)	
**HLA-A Position**	**Possible AA**	**P**	**P (Conditional)**	**P**	**P (Conditional)**
10	L,V	1.27e-05	4.00e-01	2.18e-02	4.49e-02
14	L,S	4.66e-12	7.83e-01	2.39e-01	4.94e-01
35	T,S,F,Y	6.20e-12	6.02e-01	**5.76e-05**	NA
82	R,G	1.29e-02	6.73e-01	5.12e-02	7.93e-02
88	R,E,Q,G	1.07e-07	6.24e-01	1.60e-03	3.28e-03
89	N,E	1.27e-08	2.72e-01	2.15e-02	4.20e-02
91	G,R	2.72e-01	6.06e-01	4.80e-01	3.13e-01
92	N,K	8.87e-05	5.81e-01	5.13e-03	1.03e-02
96	Q,H	2.02e-02	8.64e-01	1.17e-02	3.47e-03
99	I,T	**2.90e-13**	NA	1.52e-02	NA
100	H,D	1.93e-03	2.65e-01	1.04e-04	2.28e-04
102	A,E,V	4.43e-01	4.32e-01	1.32e-01	7.97e-02
103	N,D	3.48e-01	3.77e-01	9.19e-02	4.79e-02
105	R,G	1.26e-01	2.39e-01	2.16e-01	1.32e-01
106	I,T	1.26e-01	2.39e-01	2.16e-01	1.32e-01
107	A,L	1.26e-01	2.39e-01	2.16e-01	1.32e-01
108	L,R	1.26e-01	2.39e-01	2.16e-01	1.32e-01
109	R,G	1.26e-01	2.39e-01	2.16e-01	1.32e-01
116	D,A	1.48e-03	4.93e-01	1.69e-01	7.59e-02
121	L,V,I	2.31e-04	4.52e-01	6.85e-04	1.36e-03
123	I,R,M	2.00e-06	9.76e-01	7.50e-04	1.57e-03
125	C,F,Y	1.94e-01	4.81e-01	2.77e-01	1.68e-01
131	P,S	1.22e-03	2.01e-01	1.24e-01	4.91e-02
133	W,G	3.21e-03	3.01e-01	2.28e-04	5.07e-04
140	R,Q,H	3.86e-11	1.12e-01	9.45e-03	1.89e-02
142	D,Y	7.96e-05	1.04e-01	2.65e-03	5.82e-03
153	N,K	6.87e-05	2.87e-01	6.28e-03	1.35e-02
168	T,I	2.51e-03	3.00e-01	2.89e-04	6.41e-04
170	Q,K	2.27e-11	9.42e-02	5.88e-02	9.70e-02
171	H,R	2.51e-03	3.00e-01	2.89e-04	6.41e-04
175	T,A	1.79e-01	2.95e-01	6.22e-02	4.32e-02
177	R,H	1.17e-11	2.63e-01	1.54e-02	NA
178	E,A,V	5.68e-03	5.09e-01	6.66e-03	1.45e-03
196	W,Q,L	7.66e-05	8.45e-01	1.25e-02	7.58e-04
203	R,T	2.41e-03	7.35e-01	5.75e-02	2.38e-02
206	D,E	2.94e-01	4.85e-01	4.06e-01	2.75e-01
207	G,W	2.94e-01	4.85e-01	4.06e-01	2.75e-01
227	A,P	2.10e-03	3.42e-01	2.29e-03	5.06e-03
236	P,A	5.79e-03	6.67e-01	2.37e-02	2.35e-03
237	I,V	5.78e-03	6.67e-01	2.42e-02	2.39e-03
250	G,S	5.79e-03	6.67e-01	2.37e-02	2.35e-03
289	S,A	1.24e-09	5.32e-01	8.00e-02	1.70e-01
296	E,Q	5.66e-03	7.32e-01	2.34e-02	2.33e-03
319	L,P	4.86e-02	6.74e-01	6.38e-02	2.42e-02
325	V,I	3.06e-01	3.57e-01	2.56e-01	1.58e-01
337	L,F	5.04e-03	6.32e-01	2.23e-02	2.63e-03
341	F,I	3.00e-12	6.73e-01	7.60e-02	1.58e-01
342	A,T	5.51e-11	6.50e-01	1.03e-01	2.20e-01
350	R,M	3.00e-12	6.73e-01	7.60e-02	1.58e-01
354	N,K	3.06e-01	3.57e-01	2.56e-01	1.58e-01
364	T,S	2.59e-02	3.88e-01	1.33e-01	5.40e-02
377	M,V	3.13e-11	3.65e-01	1.97e-01	4.16e-01

Table S5: Association between HLA-A amino acid positions and HBV amino acid variants in the European cohort. P-values based on the omnibus test. The conditional p-value represents the association conditioned on the top association (in bold).

		Precore/core position 67 (Associated AA: Y)	
HLA-A Position	Possible AA	P	P (Conditional)
10	L,V	6.62e-10	4.21e-08
14	L,S	2.31e-08	5.94e-07
35	T,Y,S,F	1.70e-09	1.05e-07
91	G,R	6.37e-09	9.99e-08
92	K,N	4.41e-10	1.85e-08
96	Q,H	7.45e-09	1.82e-07
100	H,D	1.04e-10	4.19e-09
102	A,E,V	1.81e-08	1.41e-07
103	N,D	3.89e-08	3.98e-08
105	R,G	1.57e-09	1.30e-08
106	I,T	6.69e-10	1.20e-08
107	A,L	6.69e-10	1.20e-08
108	L,R	6.69e-10	1.20e-08
109	R,G	6.69e-10	1.20e-08
116	D,A	1.66e-10	2.03e-10
121	L,V,I	2.67e-10	1.06e-08
123	I,M,R	2.66e-10	1.28e-08
125	F,Y	1.26e-09	1.45e-08
131	P,S	1.18e-09	2.88e-09
133	W,G	1.25e-10	4.79e-09
140	Q,R,H	3.46e-07	1.51e-07
142	D,Y	1.24e-07	5.86e-08
153	N,K	6.66e-11	2.53e-09
168	T,I	1.42e-10	4.91e-09
170	Q,K	**5.86e-11**	NA
171	H,R	1.42e-10	4.91e-09
177	R,H	2.02e-09	6.42e-09
178	E,V	1.21e-07	5.86e-07
201	D,E	7.41e-10	2.32e-08
203	R,T	1.29e-08	1.45e-07
206	D,E	7.83e-09	1.28e-07
207	G,W	7.83e-09	1.28e-07
227	A,P	1.65e-09	1.25e-07
236	P,A	4.27e-09	2.44e-07
237	I,V	2.73e-09	1.37e-07
250	G,S	5.28e-09	2.76e-07
289	S,A	1.42e-08	1.17e-06
296	E,Q	3.26e-09	1.95e-07
319	L,P	3.82e-05	8.83e-04
325	V,I	6.87e-05	1.53e-03
337	L,F	4.25e-05	9.18e-04
341	F,I	8.56e-05	1.42e-03
342	A,T	8.45e-05	1.15e-03
350	R,M	8.56e-05	1.42e-03
354	N,K	6.87e-05	1.53e-03
364	T,S	3.82e-05	8.83e-04
377	M,V	8.15e-05	1.71e-03

Table S6: Interface template-modelling (ipTM) score and average interface predicted aligned error (PAE) scores of top-ranking models. Models were constructed based on wildtype (WT) NTCP or NTCP S267F, along with a peptide derived from the HBV genotype C wildtype (WT) preS1 sequence or with the introduction of associated preS1 mutations. Average interface PAE was calculated based on the mean across pairs of NTCP and preS1 residues (upper right box of PAE matrices, **Figure S12A and S12C**).

	**preS1 WT**	**preS1 S17A, G35R, H51P**	**preS1 S17A**	**preS1 G35R**	**preS1 H51P**
**NTCP WT**	ipTM: 0.34<br>PAE: 16.84	ipTM: 0.34<br>PAE: 17.25	ipTM: 0.27<br>PAE: 20.10	ipTM: 0.33<br>PAE: 20.16	ipTM: 0.34<br>PAE: 16.95
**NTCP S267F**	ipTM: 0.31<br>PAE: 18.87	ipTM: 0.31<br>PAE: 18.64	ipTM: 0.22<br>PAE: 21.12	ipTM: 0.3<br>PAE: 18.89	ipTM: 0.32<br>PAE: 18.38

Table S7: Population frequency of residues at position 17 of HBV preS1 (normalized to Genotype C) according to HBVdb. Asterisks indicate that the study the mutation (S17A) was observed included individuals with Asian ancestry in a country outside of Asia.

	**Genotype A**		**Genotype B**		**Genotype C**		**Genotype D**	
	**A**	**S**	**A**	**S**	**A**	**S**	**A**	**S**
**Australia and New Zealand**	0	1	**1***	24	**1***	29	0	34
**Central Asia**	0	4	0	0	0	2	0	18
**East Asia**	0	71	**24**	1901	**11**	1941	0	80
**Europe**	0	181	0	8	0	13	0	433
**Central and South America**	0	124	0	9	**1***	8	0	100
**Middle East and North Africa**	0	1	0	0	0	0	0	240
**North America**	0	470	**1**	315	0	523	0	137
**Polynesia and Micronesia**	0	0	0	0	0	9	0	5
**South Asia**	0	41	0	0	0	44	0	255
**South-East Asia**	**1**	10	**2**	373	**3**	293	0	7
**Sub-Saharan Africa**	0	263	0	0	0	2	0	89

Table S8: Predicted epitopes overlapping HBV amino acid positions. HLA-associated HBV amino acid positions are annotated in bold within the epitope sequences. Binding affinities are based on two algorithms (MixMHCpred and NetMHCpan). Experimental assay results are based on epitope sequence with exact match on IEDB (accessed June 2021).

Peptide Sequence	MixMHCpred EL Percentile Rank (%)	NetMHCPan IC50 (nM)	NetMHCpan EL Percentile Rank (%)	IEDB MHC Ligand Assays (# Positive / All)	IEDB T-Cell Assays (# Positive / All)
**Position 67 precore/core (G2G Association: HLA-A*01:01)**					
LLDTASAL**Y** (IEDB Epitope ID: 37181)	0.01	7.62	0.01	HLA-A*01:01 (1/1)<br>HLA-A*02:01 (0/1)	Qualitative binding (2/2)<br>IFNg release (1/1)
**Position 49 polymerase (G2G Association: HLA-A*02:06)**					
V**S**IPWTHKV	0.13	36.59	0.06	None	None
**Position 160 precore/core (G2G Association: HLA-A*33:03)**					
WIRTPP**A**YR (IEDB Epitope ID: 72649)	0.26	16.13	0.05	HLA-A*02:01 (0/1)	None

Supplementary Methods 1

Using AlphaFold-Multimer, a model was constructed for each combination: presence or absence of the NTCP S267F variant and the associated preS1 mutations. To quantify each model's support of preS1-NTCP binding, we focused on two metrics: the predicted interface template-modeling (ipTM) score and the predicted aligned-error (PAE) score. The ipTM score is a global accuracy measure of interface residues (between NTCP and preS1), with a higher value indicating higher accuracy and thus higher support of NTCP-preS1 binding. The PAE score is a per-residue accuracy metric between pairs of residues (x,y). The score refers to the expected position error at residue x if the predicted and true structures were aligned on residue y. We calculated the average PAE of the interface by including all residue pairs within the upper right box of the PAE matrices (**Figure S12A and S12C)**. Lower average PAE values within the interface indicate higher support of NTCP-preS1 binding. We did not include the lower left box of the PAE matrices to calculate the average PAE of the interface. This is because the pLDDT scores of preS1 residues, referring to the per-residue local confidence of the preS1 structure itself, were low (**Figure S12B and S12D)** and thus cannot be reliably aligned on.

First, we tested the independent effect of the NTCP S267F variant. Similar to the NTCP WT model, it is important to note that the prediction confidence for both the preS1-NTCP interface residues (**Figure S12C**) and the preS1 structure itself (**Figure S12D**) was also relatively low here. We compared the model of NTCP WT against the model of NTCP S267F, both in the presence of preS1 WT. By focusing on average PAE of the interface (dotted upper right box of **Figure S12A and S12C)**, we observed lower PAE in the NTCP WT model compared to the NTCP S267F model (**Table S6)**. A higher ipTM was also observed (**Table S6).** This corresponds to previous studies that establish the effect of NTCP S267F on inhibiting preS1 binding. Next, we tested the effects of associated preS1 mutations, either in the presence or absence of NTCP S267F. Focusing on models with NTCP S267F, the difference in PAE and ipTM in the presence or absence of all three associated preS1 mutations was minimal (**Table S6).** Minimal difference was also observed between models with NTCP WT. Given the relatively low overall confidence of the interface and preS1 structure, AlphaFold may not be sensitive enough to detect any changes in binding affinity caused by the associated preS1 mutations.

Supplementary Methods 2

Cell lines and maintenance

HepG2 and HEK293T cells were maintained in Dulbecco's Modified Eagle Medium (DMEM), supplemented with 10% FBS, 2 mM L-glutamine, 50 U/mL penicillin, 50 µg/mL streptomycin. For lentivirus production HEK293T cells were transfected using LT1 Transfection reagent with plasmid harboring WT NTCP and NTCP S267F variant both with a HA tag at the N terminus. HEK293T supernatant was collected from 24h to 48 h post transfection and ultracentrifuged for 2 hours at 20000 rpm. The lentivirus pellet was resuspended in DMEM medium and HepG2 cell transduced using 100 µl. NTCP expressing HepG2 were maintained under puromycin selection.
Taurocholate Uptake Assay

To measure the functionality of the stable expressed HA-NTCP a taurocholate uptake assay was performed. 24 hours after seeding, HepG2, HepG2 NTCP A3 clone (used as positive control), HepG2 HA-NTCP WT and HepG2 HA-NTCP S267F cells were incubated with taurocholate (150 mM containing 450 cpm/fmol [3H] taurocholate). Transport was stopped after 5 minutes by removing the medium and washing 3x with ice-cold PBS. Cells were lysed (0.2 M NaOH and 0.05% sodium dodecyl sulfate). Radioactivity of cell lysates was measured in a liquid scintillation counter (Packard Instruments, Frankfurt, Germany) using Ultima Gold liquid scintillation solution (Perkin Elmer, Rodgau, Germany).

Western Blot

Western blot analysis was performed on HepG2, HepG2 NTCP WT and HepG2 NTCP S267F cell lysates to quantify the expression level of NTCP. NTCP signals were detected using HA tag antibody (BioLegend Cat. 901502), quantified using Image Studio and normalized on the actin values.

Immunofluorescence

To assess NTCP cellular localization and expression after lentivirus transduction, HepG2, HepG2 NTCP WT and HepG2 NTCP S267F cells were fixed with 4% PFA and permeabilized at RT for 10 min. The cells were incubated with HA tag antibody (BioLegend Cat. 901502) in 2% BSA/PBS at RT for 1 hour. After washing with PBS, cells were incubated with 1:1000 diluted secondary antibody (goat anti rabbit Alexa Fluor 488) and 2 µg/mL Hoechst in the same solution as the secondary antibody for 1 hour at RT, protected from light. Imagines were acquired on fluorescence microscopy.

Synthesis of myristoylated peptides

WT and ARP myristoylated peptides were synthesized by solid phase peptide synthesis employing the Fmoc/tBu strategy with HBTU/DIPEA activation in an Applied Biosystems 433A peptide synthesizer. Atto565-maleimide was linked to the lysine (K) at position 59. The identity of the peptides was controlled by mass spectrometry.
